# *Staphylococcus aureus* isolates from Eurasian Beavers (*Castor fiber*) carry a novel phage-borne bicomponent leukocidin related to the Panton-Valentine leukocidin

**DOI:** 10.1038/s41598-021-03823-6

**Published:** 2021-12-22

**Authors:** Stefan Monecke, Andrea T. Feßler, Sindy Burgold-Voigt, Henrike Krüger, Kristin Mühldorfer, Gudrun Wibbelt, Elisabeth M. Liebler-Tenorio, Martin Reinicke, Sascha D. Braun, Dennis Hanke, Celia Diezel, Elke Müller, Igor Loncaric, Stefan Schwarz, Ralf Ehricht

**Affiliations:** 1 Leibniz Institute of Photonic Technology (Leibniz—IPHT), Jena, Germany; 2 InfectoGnostics Research Campus, Jena, Germany; 3 Institute for Medical Microbiology and Virology, Dresden University Hospital, Dresden, Germany; 4 Institute of Microbiology and Epizootics, Freie Universität Berlin, Berlin, Germany; 5 Department of Wildlife Diseases, Leibniz Institute for Zoo and Wildlife Research, Berlin, Germany; 6 Friedrich-Loeffler-Institute (Federal Research Institute for Animal Health), Institute of Molecular Pathogenesis, Jena, Germany; 7 Institute of Microbiology, University of Veterinary Medicine, Vienna, Austria; 8 Institute of Physical Chemistry, Friedrich-Schiller University, Jena, Germany

**Keywords:** Ecology, Microbiology, Molecular biology, Zoology

## Abstract

*Staphylococcus aureus* can be a harmless coloniser, but it can also cause severe infections in humans, livestock and wildlife. Regarding the latter, only few studies have been performed and knowledge on virulence factors is insufficient. The aim of the present study was to study *S. aureus* isolates from deceased wild beavers (*Castor fiber*). Seventeen isolates from eleven beavers, found in Germany and Austria, were investigated. Antimicrobial and biocide susceptibility tests were performed. Isolates were characterised using *S. aureus*-specific DNA microarrays, *spa* typing and whole-genome sequencing. From two isolates, prophages were induced by mitomycin C and studied by transmission electron microscopy. Four isolates belonged to clonal complex (CC) 8, CC12, and CC398. Twelve isolates belonged to CC1956 and one isolate was CC49. The CC49 and CC1956 isolates carried distinct *lukF/S* genes related to the Panton-Valentine leukocidin (PVL) from human isolates of *S. aureus*. These genes were located on related, but not identical, *Siphovirus* prophages. The beavers, from which those isolates originated, suffered from abscesses, purulent organ lesions and necrotising pneumonia, i.e., clinical manifestations resembling symptoms of severe PVL-associated disease in humans. It might thus be assumed that the “Beaver Leukocidin (BVL, *lukF/S*-BV)”-positive strains are beaver-specific pathogens, and further studies on their clinical role as well as on a possible transmissibility to other species, including humans, are warranted.

## Introduction

*Staphylococcus aureus* is a widespread and extremely versatile bacterium that colonises about 20–30% of any human population. Beyond asymptomatic, usually nasal, colonisation, it is also able to cause clinical infections ranging from superficial skin and soft tissue infections (SSTI) to life-threatening conditions such as necrotizing fasciitis, septicaemia, endocarditis or pneumonia. An important public health issue is the presence of antimicrobial resistance genes on mobile genetic elements spreading across different *Staphylococcus* species including *S. aureus*. The most notable examples are *mec* genes on horizontally transmissible mobile genetic elements, so-called Staphylococcal Cassette Chromosome *mec* (SCC*mec*) elements, that gave rise to genetically diverse methicillin-resistant *S. aureus* (MRSA).

*S. aureus* carriage, or infection, is not restricted to humans. *S. aureus* has been found in a wide variety of wildlife and domestic animals (for details and references, see Supplemental File 1). In general, domestic animals or animals in zoos or wildlife rehabilitation centres might have acquired *S. aureus* directly from humans or can be involved into chains of transmissions of very specific clones such as livestock-associated MRSA. Wild animals usually carry *S. aureus* belonging to poorly known lineages that are usually only identified in conspicuous outbreaks^1,2^ or if they cause concern related to human health and antimicrobial resistance, such as in the case of *mecC*. Few systematic studies have yet been performed in wildlife so evidence is anecdotal and knowledge on wildlife lineages of *S. aureus* is still sparse. It can be assumed that *S. aureus* causes similar conditions in animals as in humans. Many animals might be asymptomatically colonised by *S. aureus* while in some cases, purulent SSTI such as abscesses as well as pneumonia or sepsis might be observed. This raises the question whether the multitude of virulence factors known from *S. aureus* might be related to a host specificity of the strains they reside in, i.e., if an apparent redundancy of virulence factors serves as adaption to diverse host organisms.

One major class of *S. aureus* virulence factors are bicomponent leukocidins that consist of two components aggregating to polymeric pores in host leukocyte membranes leading to cell death. They are encoded by pairs of co-localised and co-expressed genes^3,4^. Some can be essentially found in every *S. aureus* isolate, regardless of host specificity and clonal complex (CC) affiliation. These include *lukA/B* (with *lukG/H* and *lukX/Y* being synonyms^5–8^) and *lukF/S* from the haemolysin gamma gene locus which could be regarded as species markers, similarly as *lukF/S*-int in *Staphylococcus intermedius*/*pseudintermedius*.

Moreover, there are *lukD/E* genes which are located on a genomic island that is absent from some *S. aureus* lineages (CC9, CC22, CC30, CC45, CC59, CC93, CC182, CC398, CC509 and CC772) as well as from most of the CCs recently re-categorised as *Staphylococcus argenteus*. Generally, there is no variation between isolates and strains belonging to the same lineage although few isolates might show random deletions of *lukD/E* and neighbouring genes.

Finally, there are different phage-borne genes *lukF/S-PV, lukM/lukF-P83* and *lukP/Q* distributed across different lineages. The presence of Panton-Valentine leukocidin (*lukF/S-PV*, PVL) is strongly associated with SSTI in humans, such as abscesses and carbuncles, but it can also be identified in rapidly progressing, life-threatening necrotising pneumonia. It has first been described nearly a century ago, and was associated with worldwide outbreaks in the 1940s and 1960s as well as with a recent global emergence of virulent, community-associated MRSA^9–14^. *LukM/lukF-P83*-positive isolates originate from cattle, small ruminants and, rarely, from pigs or rodents^1,11,15–18^ and *lukP/Q* was recently observed in horse isolates^19^. The molecular epidemiology of these leukocidins strongly suggests host specificity, which was also supported by results of an experimental study indicating that PVL is effective in killing neutrophils of humans and rabbits, but not of macaques and mice^20^. As genes of these bicomponent leukocidins are located on prophages, one might speculate that the infection of *S. aureus* by bacteriophages predetermines both, host specificity of a given *S. aureus* strain as well as clinical manifestation in the vertebrate host of origin.

In this study, we present additional evidence for host specificity by describing a novel phage-borne bicomponent leukocidin related to PVL from diseased Eurasian (or European) beavers (*Castor fiber*).

## Results

### Isolates and typing

**Figure 1:**
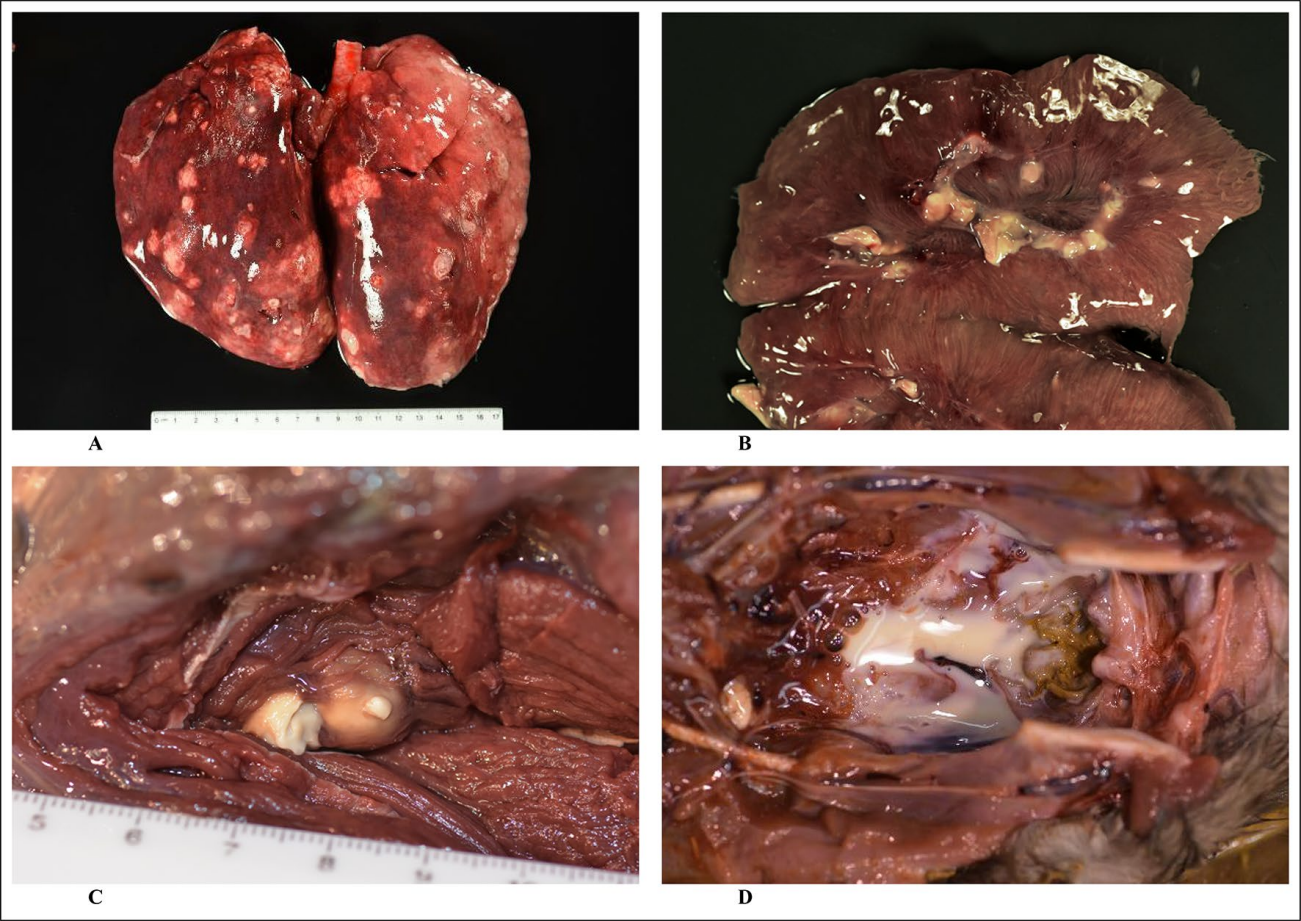
Pathological lesions of Eurasian beavers (C*. fiber*) infected with BVL-positive *S. aureus*. (**A**) Severe suppurative necrotizing pneumonia (animal B); (**B**) severe suppurative pyelonephritis (animal G); (**C**) caseous lymphadenitis, popliteal lymph node (animal E); (**D**) urinary bladder with pyuria (animal C).

The isolates characterised as well as strain affiliations, geographic origins and clinical presentations are summarised in Table 1. Autopsy images showing typical aspects of putrid infections in some animals are shown in Fig. 1. The complete microarray hybridisation patterns are provided as Supplemental file 2 and some relevant features will be discussed in the descriptions of the respective strains. While all German isolates yielded hybridisation signals for *lukF/S-*PV, frequently only weak positive or ambiguous results for the *lukS-*PV probe were observed. This prompted further investigations, including the detection of PVL by lateral flow assay^21^ (Table 1) and whole genome sequencing (see below).

**Table 1:** Details of animals, isolates and strains.

Animal	Geographic origin	Isolate	Pathology	MLST	*spa*	Strain ID according to array	PVL by lateral flow
**A**	Berlin	WT19	Multifocal moderate to severe suppurative necrotising pneumonia and suppurative pyelonephritis. Found dead.	ST4614^a^	t3058	CC1956-MSSA (*lukF*-PV + /*lukS*-PV?)^b^	Positive
**B**	Berlin	WT63	Severe suppurative necrotising pneumonia and multiple small abscesses in spleen, kidney, caecum, mesenteric lymph nodes and intercostal muscles. Found dead.	ST4614	t3058	CC1956-MSSA (*lukF*-PV + /*lukS*-PV?)	Positive
		WT64		ST4614	t3058	CC1956-MSSA (*lukF*-PV + /*lukS*-PV?)	Positive
**C (“Tiffy”)**	Bavaria	WT65	Severe abscessing mandibular lymphadenitis, severe suppurative cystitis. Died in a wildlife rehabilitation centre.	ST49	t208	CC49-MSSA (*lukF*-PV + /*lukS*-PV?)	Positive
**D**	Berlin	WT66	Severe fibrinous-purulent myocarditis, suppurative pyelonephritis (right kidney only) and prostatitis. Found dead.	ST4614	t3058	CC1956-MSSA (*lukF*-PV + /*lukS*-PV?)	Positive
		WT67a		ST4614	t3058	CC1956-MSSA (*lukF*-PV + /*lukS*-PV?)	Positive
		WT67b		ST4614	t3058	CC1956-MSSA (*lukF*-PV + /*lukS*-PV?)	Positive
		WT68		ST4614	t3058	CC1956-MSSA (*lukF*-PV + /*lukS*-PV?)	Positive
		WT69		ST4614	t3058	CC1956-MSSA (*lukF*-PV + /*lukS*-PV?)	Positive
**E**	Berlin	WT70	Suppurative necrotising pneumonia, suppurative pyelonephritis and lymphadenitis (popliteal lymph nodes), multiple abscesses in caecum wall. Found dead.	ST4614	t3058	CC1956-MSSA (*lukF*-PV + /*lukS*-PV?)	Positive
**F**	Berlin	WT71	Abscesses subcutaneous (chest wall/axilla) and in popliteal lymph nodes. Euthanised due to poor physical condition.	ST4614	t3058	CC1956-MSSA (*lukF*-PV + /*lukS*-PV?)	Positive
**G**	Berlin	WT110	Suppurative necrotising pneumonia, enlarged thyroid gland containing small yellowish abscesses, suppurative pyelonephritis, splenomegaly, multiple abscesses in caecum and colon walls as well as in skin. Cachexia. Found dead.	ST4614	t3058	CC1956-MSSA (*lukF*-PV + /*lukS*-PV?) ^c^	Positive
		WT111		ST4614	t3058	CC1956-MSSA (*lukF*-PV + /*lukS*-PV?) ^c^	Negative
**H**	Marchfeld Channel, Austria	B1	Nasal swab. Good physical condition except for gunshot traumata including parts of a bullet in the right hindleg with surrounding acute necrotizing inflammation. All other organs unremarkable.	N/A	t085	CC398-MSSA	N/A
**I**	Lower Austria	B2	Abscessing lymph node, knee. Shot dead.	N/A	t008	CC8-MSSA	N/A
**J**	Eisenstadt region, Austria	B3	Pneumonia (*Streptococcus pyogenes, Morganella morganii*). Splenomegaly. *S. aureus* originated from enrichment culture of lung tissue. Found dead.	N/A	t394	CC8-MSSA (*sed/j/*r +)	N/A
**K**	Lower Austria	B4	Nasal Swab. Healthy animal shot dead.	N/A	t156	CC12-MSSA (*sep* +)	N/A

^a^ST4614 is a single locus variant of ST1956; *arcC*-6, *aroE*-291, *glpF*-6, *gmk*-2, *pta*-7, *tpi*-225, *yqiL*-585.

^b^Clearly positive for *lukF-*PV, weekly positive or ambiguous signal for *lukS-*PV.

^c^WT110 and WT111 differed in haemolysis on Columbia blood agar and were thus handled separately although array analysis eventually revealed identical strain affiliations.

### Phenotypic and genotypic resistance properties of the *S. aureus* isolates

Antimicrobial susceptibility testing revealed that all beaver isolates from Germany were susceptible to all antimicrobial agents tested. The distribution of minimal inhibitory concentration (MIC) values and test ranges are displayed in Supplemental File 3a. The phenotypic data corresponded well with microarray data, since none of the corresponding resistance genes was identified. In contrast, two of the Austrian isolates showed macrolide resistance with one of them also being lincosamide resistant. One isolate also exhibited tetracycline resistance. These phenotypes corresponded with the detection of genes *erm*(A), *erm*(C) and *tet*(M), respectively (Supplemental file 2 and 3b).

The chromosomal variant of the metallothiol transferase gene *fosB* was present in all CC1956 isolates. Sequence analysis revealed a frame shift at position 108 creating a stop codon at positions (pos.) 146.0.148 compared to the reference sequence (N315, GenBank BA000018.3 [2,389,328.0.2,389,747]). This resulted in a truncated protein of 48 amino acids (aa) rather than 139 aa as for the original *fosB* gene product. The mutation was present in all available sequences (i.e., Oxford Nanopore and Illumina of WT19 as well as Illumina of WT63, WT64, WT66, WT67a, WT67b, WT68, WT69, WT70, WT71, WT110 and WT111). While *fosB* was originally implicated in fosfomycin resistance, it appears to be linked to certain CCs. Indeed, it was also present in the CC8 and CC12 beaver isolates (B2, B3, B4) as well as in the reference sequences of the respective CCs (Supplemental File 2). The *fosB* gene was absent from the CC49 isolate WT65 and from the CC49 reference sequence of Tager 104, GenBank CP012409.1, as well as from the CC398 isolate B1. Moreover, all sequenced isolates (from animals A to G) harboured a gene designated *tet*(38), encoding a major facilitator superfamily permease. While this gene was implicated in low-level tetracycline resistance when overexpressed^22^, its mere presence certainly is not associated with phenotypic tetracycline resistance as it can be found in virtually every *S. aureus* genome.

Biocide susceptibility testing of the CC49/1956 isolates revealed unimodal MIC distributions (Supplemental File 3b), with ranges encompassing not more than three to four dilution steps for each of the biocides (benzalkonium chloride, 0.00003–0.00025%; polyhexanide, 0.000125–0.0005%; chlorhexidine, 0.00006–0.00025% and octenidine, 0.00006–0.00025% with percentages given as mass per volume). The four remaining isolates showed MIC values of 0.0000125–0.00025% for benzalkonium chloride, 0.0005–0.001% for polyhexanide, 0.00006–0.000125% for chlorhexidine, and 0.000125–0.00025% for octenidine.

The chromosomal heavy metal resistance markers *arsB/R* and *czrB* were detected by hybridisation in all four CC1956 isolates tested as well as in the CC49 isolate. This was confirmed by sequencing. There was no evidence for plasmid- or SCC-borne heavy metal resistance markers.

### The sequence of the phage-borne leukocidin genes in WT19 and WT65

**Figure 2:**
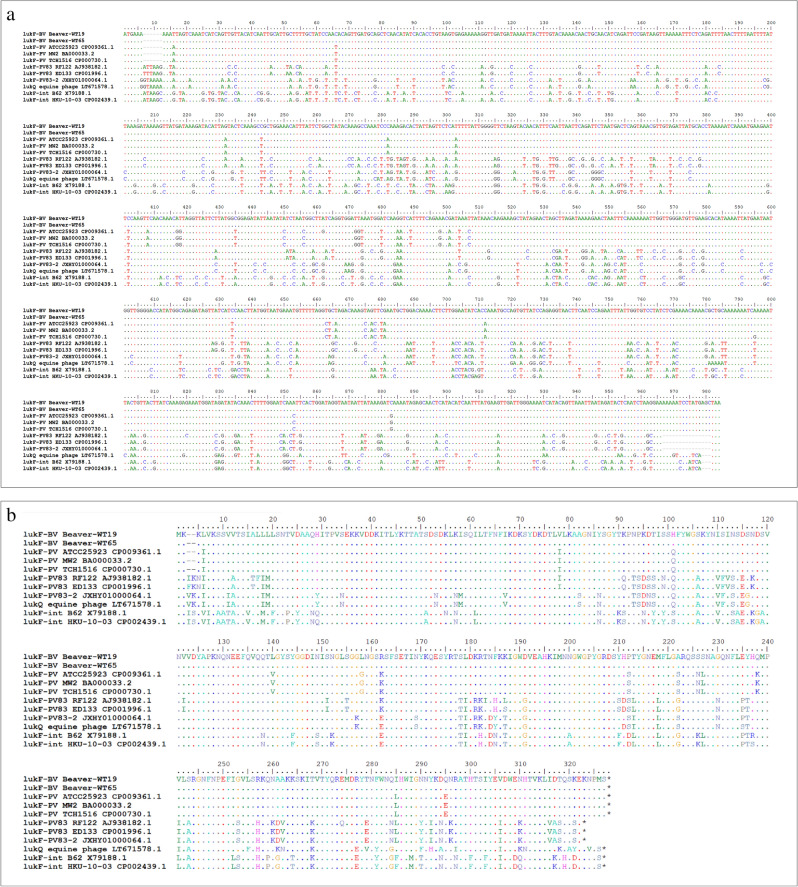
(**a**) Alignment of the *lukF-*BV sequences, of other phage-borne leukocidin F component sequences from *S. aureus* and of *lukF*-int from *S. intermedius/pseudintermedius*. (**b**) Alignment of the amino acid sequences of the corresponding *lukF* gene products.

As mentioned above, CC49/CC1956 beaver isolates yielded occasionally ambiguous hybridisation intensities for *lukS*-PV probes prompting further investigation assuming that the specifically designed oligonucleotides were not able to bind optimally at the target due to mismatches, i.e., allelic variants. Sequencing revealed the presence of distinct alleles of phage-borne leukocidin genes (Figs. 2a/b and 3a/b). The sequences from the two sequenced beaver isolates were identical to each other despite their origin from different prophages in different CCs. In general, the beaver alleles, hitherto referred to as “Beaver Leukocidin” or BVL, *lukF/S*-BV, appeared to be closer related to the PVL genes from human strains of *S. aureus* than to those from ruminants and horses (see Figs. 2a/b and 3a/b and the percentages of homologies as provided in Supplemental File 4). There was no evidence for recombination/chimerism in *lukF*-BV and *lukS*-BV as mismatches compared to other sequences were evenly distributed across the entire sequences. Sequences of *lukF-*BV and *lukS*-BV were also related but clearly distinct from core genomic *lukF/S*-int of *S. intermedius/pseudintermedius.*

**Figure 3:**
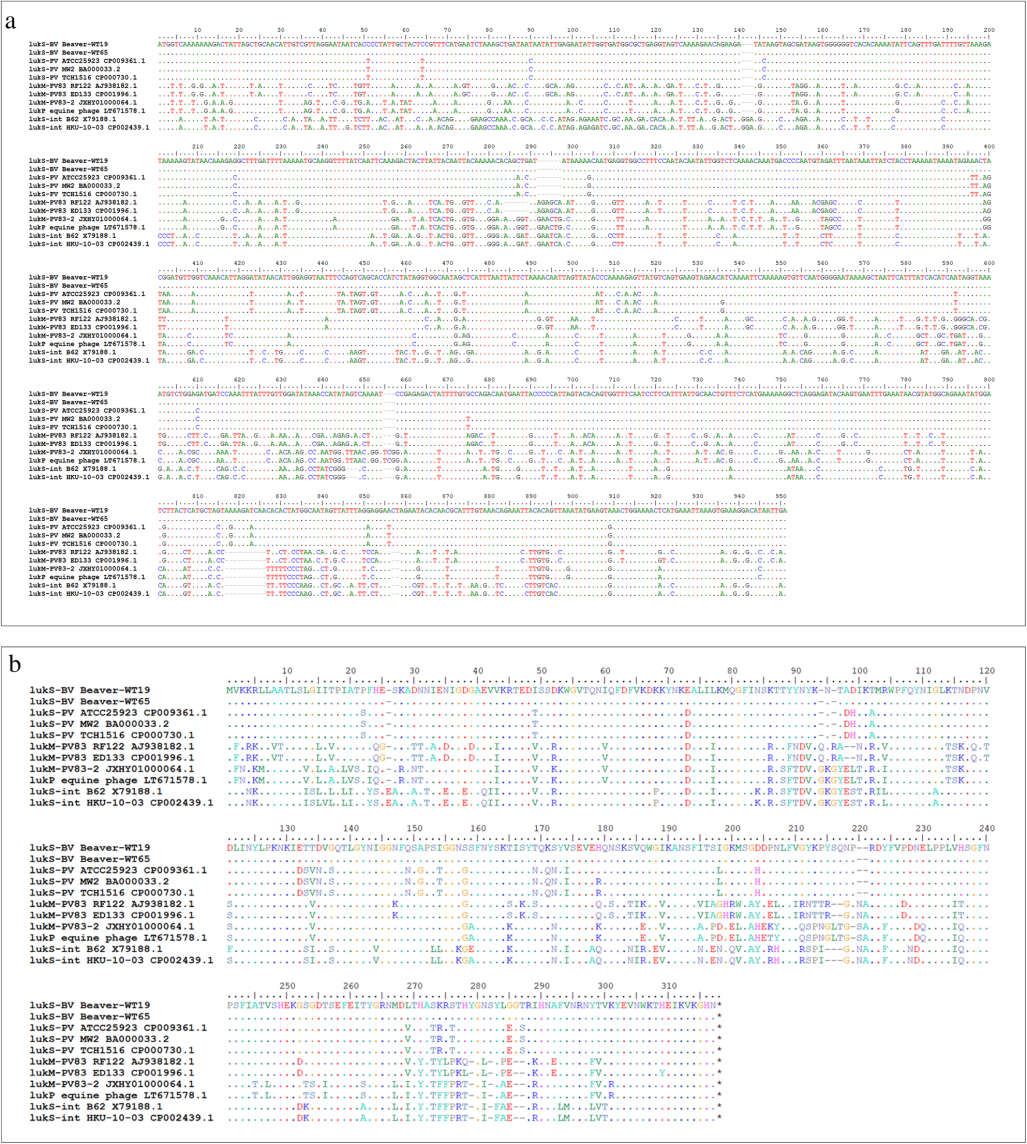
(**a**) Alignment of the *lukS-*BV sequences, of other phage-borne leukocidin S component sequences from *S. aureus* and of *lukS*-int from *S. intermedius/pseudintermedius.* (**b**) Alignment of the amino acid sequences of the corresponding *lukS* gene products.

### *lukF/S*-BV and the *agr* locus

Two isolates from one animal, WT110 and WT111 (Table 1), differed in hemolysis on Columbia blood agar and were thus handled separately although array analysis eventually revealed the same strain affiliations. They also differed in BVL production as shown by lateral flow tests. Sequencing using both, Illumina and Oxford nanopore technologies, revealed a substitution from A to T in position 706 of the *agrA* gene that results in a premature stop codon at position 236 of the *agrA* gene product (Supplemental File 5) suggesting that *agr* played a role in the observed phenotype and the regulation of BVL.

### Core genome and genomic islands of the CC1956 isolate WT19

As revealed by array experiments (Supplemental File 1) and confirmed by genome sequencing of WT19, CC1956 isolates presented with *agr* IV alleles and capsule type 5. They were positive for *cna*, but they lacked *seh* and *egc* enterotoxin genes, ORF CM14 as well as *sasG*. Leukocidin genes *lukX/Y*, *lukD/E* and *lukF/S-hlg* were present. This is also in accordance with previously sequenced BVL-negative CC1959 isolates (SAMEA3251370, SAMEA3251372, SAMEA3251377, SAMEA3251376, SAMEA3251380; Supplemental File 2).

The WT19 genome (Supplemental Files 6a and 6b) harboured two uncharacterised enterotoxin genes (pos. 1,940,148..1,940,900 and pos. 1,939,378..1,940,121). Both were also found in DAR4145 (CC772) where they also formed a genomic island at approximately the same position within the genome (GenBank CP010526.1: RU53_RS09775, pos. 1,968,336..1,969,061 and RU53_RS09780, pos. 1,969,088..1,969,840). One of these two genes (“*seu*2” = RU53_RS09780) was covered by the second array-based assay^23^ and it was found in all four isolates tested with this array.

### Mobile genetic elements in the CC1956 isolate WT19

**Figure 4:**
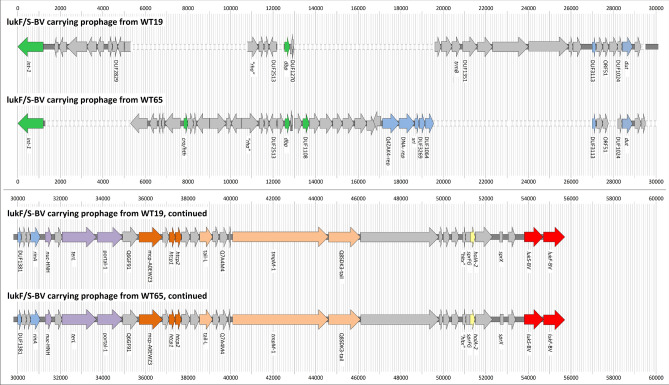
Schematic representation of the aligned sequences of the *lukF/S*-BV prophages from WT19 and WT65.

The *lukF/S*-BV prophage was integrated into the lipase 2 gene (*lip*2, “*geh*”, “*sal*3”, “*salip*35”, GenBank CP000253.1 [314,326..316,398]), and spanned pos. 322,629 to 365,636. Besides leukocidin genes, it also included genes associated with the different modules of a typical *Siphoviridae* genome (lysogeny, DNA metabolism, packaging and capsid morphogenesis, tail morphogenesis, host cell lysis^24,25^; see Supplemental File 7/Fig. 4).

Furthermore, there was a small pathogenicity island at pos. 869,706 to 884,748 that included *pif* encoding a phage interference protein, a gene for a small terminase subunit, genes for “putative proteins” as well as a gene (*scn*2) coding for a paralog of a complement inhibitor SCIN family protein and a gene for a variant of the von Willebrand factor binding protein Vwb (*vwb*3). Thus, it is considered a staphylococcal pathogenicity island (SaPI) related to the one in S0385, GenBank AM990992.1.

Another prophage integrated between *rpmF* and *isdB,* pos. 1,107,447 to 1,146,132. A third prophage was located between a truncated *nikB* and Q5HG37, pos. 1,425,279 to 1,481,870. Finally, there was a forth prophage between Q5HDU4 and *sarV* (actually interrupting an MFS transporter between those genes), pos. 2,340,832 to 2,386,591. This prophage sequence corresponded to the phage that was detected by nanopore sequencing after induction by Mitomycin C (see below and Supplemental File 8).

### Phage morphology and sequencing of phages from the CC1956 isolate WT19

In three separate preparations, large numbers of phages were observed that were well contrasted with uranyl acetate and with phosphotungstic acid. Phages had elongated capsids. The non-contractile thin tails were straight or slightly curved and ended in a bulb-shaped base plate. Based on these characteristics, they were assigned to the order *Caudovirales*, family *Siphoviridae.*

**Figure 5:**
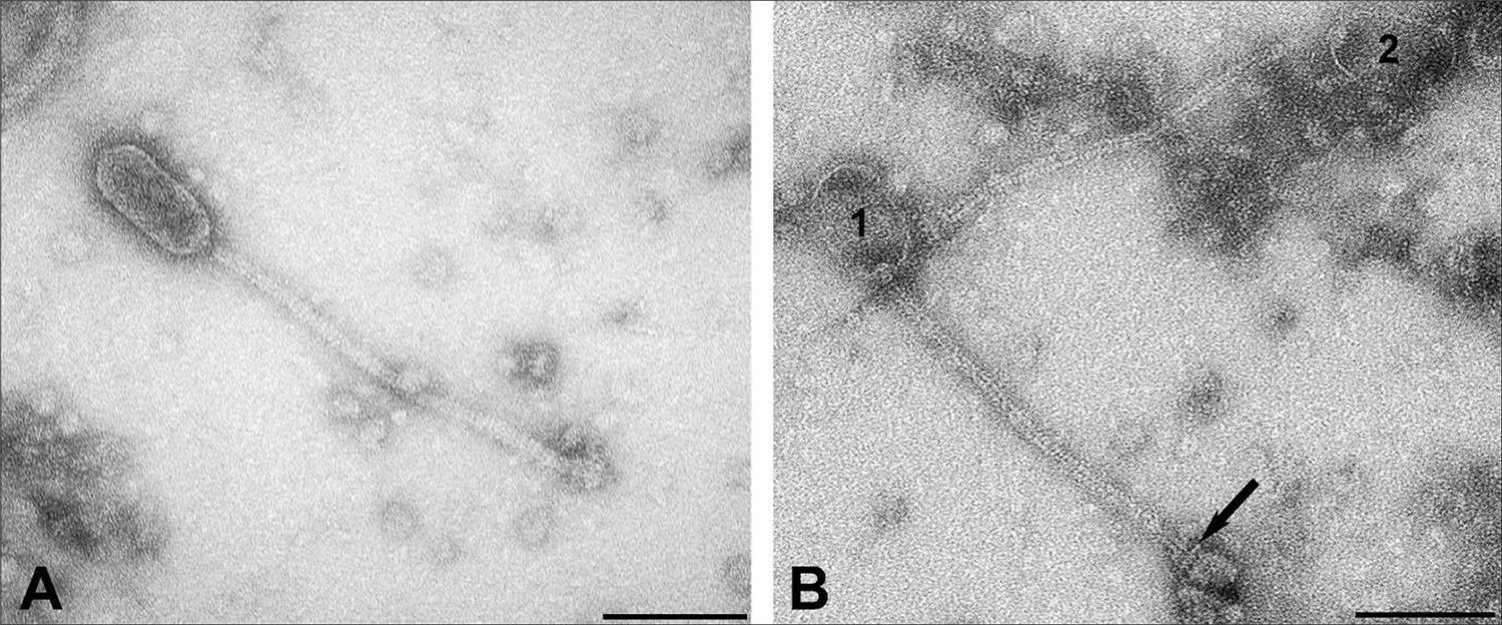
Transmission electron micrograph of two distinct prolate phages resulting from Mitomycin C treatment of *S. aureus* CC1956 isolate WT19. **A**, Phage particle with pentagonal 38 nm in diameter capsid and a 12 nm thick tail with stacked disc appearance; **B**, Two phage particles (1, 2) with oval capsids of 55 nm in diameter and 9 nm thick tails with rail-road-track morphology. The base plate is separated from the tail by a transversal disc (arrow). Negative contrast preparation with uranyl acetate. Bars = 100 nm.

Capsids were measured in 40 phages, tails in 34 and base plates in 33 phages. Based on these measurements, two distinct populations could be differentiated (Fig. 5). In one (Fig. 5A), the prolate, distinctly pentagonal capsids averaged 39 ± 5 nm (range 32–46 nm) in diameter and 92 ± 8 nm (range 80–104 nm) in length. Tails were 276 ± 20 nm (range 243–310 nm) long, had a diameter of 11 ± 1 nm (range 10–12 nm) and had a stacked discs appearance. Their baseplates were 16 nm (range 16–31 nm) by 27 nm (range 19–33 nm). The other population (Fig. 5B) had elongated oval capsids with a maximal diameter of 55 ± 2 nm (range 51–60 nm) diameter and 93 ± 5 nm (range 85–100 nm) length. Their tails measured 287 ± 12 nm (range 275–313 nm) in length and 9 ± 1 nm (8–10) in diameter and had a rail-road-track morphology. Dimensions of baseplates were 25 nm (range 21–30 nm) by 29 nm (range 23–39 nm).

Oxford Nanopore sequencing of one of these phage preparations (Supplemental File 8) yielded just one circular contig with a coverage of 724. Its sequence was identical to that of the forth prophage, between Q5HDU4 and *sarV,* except for a loss of a single triplet out of a total length of 46,387 nt*.*

### Core genome and genomic islands of the CC49 isolate WT65

The CC49 isolate carried *agr* group II alleles and capsule type 5. It was positive for *sasG*, but lacked *seh* and *egc* enterotoxin genes, ORF CM14 and the collagen adhesion gene *cna*. A truncated copy of the enterotoxin S gene (GenBank CP000046, pos. 2,203,972.0.2,204,196) was found as well as leukocidin genes *lukG/H* = *lukX/Y, lukD/E* and *lukF/S-hlg*. With regard to presence and alleles of chromosomal markers such as MSCRAMM or *ssl* genes, the genome of WT65 (Supplemental Files 7a and 7b) is closely related to the CC49 reference sequences such as Tager 104, GenBank CP012409.1 (Supplemental File 2).

### Mobile genetic elements in the CC49 isolate WT65

One prophage was integrated into the *lip2* gene spanning pos. 311,401 to 354,724. The prophage included the *lukF/S*-BV genes as well as genes associated with the different modules of a typical *Siphoviridae* genome (Supplemental File 7/Fig. 4). Sequences corresponding to the lysogeny and replication modules were clearly different compared to the *lukF/S*-BV-prophage in the CC1956 isolate WT19 while approximately the second half of the two respective prophage sequences (the lower part of the alignment in Fig. 4) were virtually identical in gene content, order and orientation.

Other mobile genetic elements (Supplemental File 9a/b) included a small pathogenicity island, pos. 402,133 to 416,237 (between *rpsR* encoding 30S ribosomal protein S18 and its terminator), that included hypothetical proteins, a gene of a terminase small subunit, *vwb*3 (encoding a “von Willebrand factor” binding protein) and the *scn*2 gene (putative paralog of complement inhibitor). Between the genes *ktrB* and *groL,* pos. 2,029,208 to 2,042,866, another SaPI was identified that contained additional, slightly different copies of *vwb*3 and *scn*2 genes as well as terminase small subunit, integrase and excisionase (*xis*-AIO21657) genes. Finally, five genes between pos.1,334,169 and 1,339,503 were annotated as phage capsid genes although no other phage-related genes were found in this region.

### Phage morphology and sequencing of phages from the CC49 isolate WT65

Four separate phage preparations were examined. In one of them, few phage-like structures were detected. These findings could not be confirmed in the following preparations. Thus, they were interpreted as artefacts, also given that it was not possible to induce a sufficient amount of phages for Oxford Nanopore sequencing.

## Discussion

There are only few reports on *S. aureus* in beavers. One paper, published in 1969, described the presence of *S. aureus* in several mammal species, including beavers, but results obtained by serological typing methods utilised then cannot be translated into CC affiliations^26^. A recent paper described a rare presence of *S. aureus* in minced beaver meat without providing further details^27^. While we are not aware of published *S. aureus* typing data from beavers (except for one MLST profile, see below), we observed CC8, CC12, CC49, CC398 and CC1956 in beavers from Germany and Austria. Isolates from two CCs, CC49 and CC1956, were conspicuous in yielding evidence for the presence of a distinct, phage-borne bicomponent leukotoxin.

CC49 is known to be associated with rodents, such as European red squirrels (*Sciurus vulgaris*)^1,2^, brown rats (*Rattus norvegicus,* see below), yellow-necked mice (*Apodemus flavicollis*) and bank voles (*Myodes glareolus*)^28^, but also with other mammals such as Australian wallabies^29^ and European wildcats (*Felis silvestris*)^30^. It has been reported from humans, causing orthopaedic infections^31^ or SSTI^32^. A livestock-associated CC49 MRSA strain with a SCC*mec* VT element has been reported from domestic pigs in Switzerland^33^ as well as *mecC-*MRSA from humans^34^ and from brown rats^35^ in Belgium. While *lukM/lukF*-P83 was reported in CC49 isolates being implicated in an outbreak of exudative dermatitis in red squirrels^1^, to the best of our knowledge no PVL genes or variants thereof have been reported from CC49 so far.

CC1956 is another rodent-associated lineage. It has been found in field voles (*Microtus agrestis*), common voles (*Microtus arvalis*) and bank voles from Germany^28,30^ as well as in common voles and wood mice (*Apodemus sylvaticus*) from Spain^36^. The MLST database^37^ provides an example of ST1956 from an English red squirrel in respiratory distress (https://pubmlst.org/bigsdb?page=info&db=pubmlst_saureus_isolates&id=3900). A single locus variant, ST1959, was observed in a beaver (*C. fiber*) from Iowa (https://pubmlst.org/bigsdb?page=info&db=pubmlst_saureus_isolates&id=3903) Other variants came from a human from Poland (ST1960, https://pubmlst.org/bigsdb?page=info&db=pubmlst_saureus_isolates&id=3904) and from an unnamed animal from Spain (ST2766, https://pubmlst.org/bigsdb?page=info&db=pubmlst_saureus_isolates&id=5333). In addition, there are a couple of sequences in the NCBI BioSample database/Sequence Read Archive (SRA; SAMEA3251370, SAMEA3251372, SAMEA3251377, SAMEA3251376, SAMEA3251380) but since they contain no additional host or geographic metadata beside a description of a “small mammal” origin, this information is essentially useless. The German vole isolates and the SRA sequences did not contain *lukF/S-*PV or *lukM/F*-P83. The ST1959 isolate from Iowa, however, was described as PVL-positive, suggesting it to be an unrecognised specimen of BVL-positive CC1956.

As both, CC49 and CC1956, are rodent-associated lineages, it can be speculated that the *S. aureus* strains belong to the native microbiome of beavers, or alternatively originate from other wild mammals not yet sufficiently studied. However, none of the yet described rodent strains harboured *lukF/S*-BV, whereas they carried either no phage-borne leukocidins, or *lukM/F*-P83. This raises the question of the origin or source of *S. aureus* infections in beavers. One might speculate that beavers were infected with human or livestock-associated strains of *S. aureus* through contact to human or domestic animal´s offal, hospital or agricultural wastewater or sewage. This could have been the case for the BVL-negative Austrian CC8, CC12 and CC398 isolates as these lineages are common, widespread and promiscuous with regard to host species^30,31,38–44^. For the CC49 and CC1956 isolates, however, the distinct and unique sequence of *lukF/S*-BV as well as the presence of identical *lukF/S*-BV alleles in different strains and on different prophages indicate a beaver-specific origin rather than accidental or interspecies infections.

All animals involved were subjected to pathological and bacteriological diagnostics and those with BVL-positive *S. aureus* presented with severe/fatal disease (Fig. 1). These observations suggest that *lukF/S-*BV in beaver strains of *S. aureus* induces essentially the same pathology as *lukF/S-*PV in human strains and that it might be associated with mortality. In the BVL-negative cases from Austria, *S. aureus* could be regarded as coloniser (B1, B3, B4) or as cause of a localised, non-fatal infection (B2). One might pointedly say that animals with BVL-negative strains died *with S. aureus* while those with BVL-positive strains died *from S. aureus* infection, but case numbers are too low yet for a definite assessment. There are no systematic data neither on *S. aureus* morbidity and mortality in beavers, nor on a possible asymptomatic carriage. Further investigations on host specificity and clinical significance would require animal experiments which are clearly beyond the scope of the present study. In the absence of such experiments, further diagnostic studies on sick or dead beavers are warranted. Investigations of asymptomatic carriage of *S. aureus* in beavers might target captive beavers as well as road-killed or shot animals which could serve as surrogate for a healthy control group, presenting at least with a non-staphylococcal cause of death as this was the case for the Austrian beavers described herein.

The geographic distribution of *lukF/S-*BV-positive *S. aureus* still needs to be studied. It was interesting that all Berlin isolates were assigned to CC1956 and the one from Bavaria to CC49 while the Austrian ones belonged to other CCs, but the number of animals studied does not yet allow to draw conclusions on the geographic range of these strains and their phages. Similarly, the prevalence of BVL-positive strains among beavers and their possible impact on wild beaver populations that just recover after a century at the brink of extinction warrant further investigation. A possible occurrence among related rodent species, such as North American beaver (*Castor canadensis*) or among those that co-exist in the same habitat, such as coypus (*Myocastor coypus*), muskrat (*Ondatra zibethicus*) or water vole (*Arvicola spec.*), as well as in other CCs of *S. aureus* also still need to be studied.

Our observations also emphasise the need for different technologies for toxin or toxin gene detection. Assays that rely on short primers and probes, such as array hybridisation or PCR, might fail to recognise deviant alleles of target genes. Protein-/antibody-based assays might be more forgiving (as it was here the case when using the PVL assay), but are, of course, less easily available. Genome sequencing is an “open” approach that can be employed to find unknown alleles or unknown genes. However, the costs in terms of both, labour and expenses, are still too high for routine applications. This restricts the use of sequencing technologies, especially if costs are an issue. Unfortunately, this is the case in veterinary settings, but also in medical settings in most parts of the world.

Finally, the question of phylogeny of leukocidin genes remains. The beaver alleles are more closely related to the PVL sequences known from human isolates of *S. aureus* and their prophages than to the *lukM/F-*P83 leukocidin sequences from ungulates or to *lukF/S-*int from canines (Figs. 2 and 3). It is tempting to assume that this might indicate a spill-over to humans from beavers hunted for and consumed as food, as recently speculated for *Yersinia* species^45^. Indeed, beavers have been hunted close to extinction, for fur as well as for meat that was allowed to be consumed during lent. Manipulation of dead animals or preparation of meat might have facilitated a transmission of *S. aureus*, and/or staphylococcal phages, but this of course cannot be proven. A possible counter argument might be the high prevalence and diversity of PVL-positive *S. aureus* in humans outside the natural range of beavers, *i.e.,* in Africa, the Indian subcontinent and the Middle East. A related issue is a potential infection risk for humans, given that human and beaver leukocidin genes are similar. Handling of dead or live beavers, for research or food^27^, should thus be performed under at least basic biosafety precautions, and the prevalence of BVL phages in beavers and related rodents should be investigated as well as possible SSTI in humans who handled beavers or beaver carcasses.

In summary, all CC49 and CC1956 isolates from fatally diseased beavers harboured the novel genes, *lukF*-BV and *lukS*-BV, which appeared to be closely related to PVL genes. Autopsy findings of affected beavers suggested significant virulence and resembled the pathology associated with PVL-positive *S. aureus* in humans. Further studies are needed to investigate the prevalence and distribution of *lukF*-BV and *lukS*-BV and its association with clinical symptoms.

## Material and methods

### Animals and isolates

Thirteen *S. aureus* isolates from seven wild beavers from Germany were investigated (Table 1). This included six adult beavers, found dead (n = 5) or moribund (n = 1) in Berlin, and one juvenile that died in captivity in Bavaria. Furthermore, four *S. aureus* isolates originated from beavers shot (n = 3) or found dead (n = 1) in Austria. Animals were subjected to post-mortem and bacteriological examinations. *S. aureus* isolates were identified by coagulase testing (BD BBL Rabbit Coagulase Plasma, Becton Dickinson, New Jersey, USA), hyaluronidase production using the *Streptococcus equi* decapsulation test^46^ and MALDI-TOF MS. Of three beavers, more than one isolate was investigated. These two (animals B, G) or five isolates (animal D) originated from different organs and/or displayed different phenotypes during primary culture, but were found to be indistinguishable upon genotyping.

### Susceptibility testing

Antimicrobial susceptibility testing was performed for 28 antimicrobial agents (Supplemental File 3a) and three combinations of antimicrobial agents by broth microdilution according to Clinical and Laboratory Standards Institute (CLSI) standards^47,48^. Biocide susceptibility testing was performed for benzalkonium chloride, chlorhexidine, octenidine, and polyhexanide using commercial microtitre plates (sifin diagnostics GmbH, Berlin, Germany) and the protocol from Schug et al.^49^ modified as follows. The inoculum was prepared by adding 30 µL bacterial suspension of a density of 0.5 McFarland to 12 mL single concentrated tryptic soy broth (TSB) and the microtiter plates were inoculated with 100 µL per well according to the manufacturer’s recommendation.

### *Spa* and multi-locus sequence typing

Multi-locus sequence typing (MLST), which is based on sequencing of fragments of *arcC, aroE, glpF, gmk, pta, tpi* and *yqiL*^50^, was performed by deducing MLST sequences and types from the Illumina sequences of all samples of animals A-G (see below). The sequences of the MLST alleles were analysed using the *S. aureus* pubMLST website^37^ (https://pubmlst.org/bigsdb?db=pubmlst_saureus_seqdef&page=sequenceQuery). *Spa* typing was performed according to previously published protocols^51^, confirming results using the Illumina sequences and using the nomenclature on the Ridom website (http://spa.ridom.de/).

### Microarray-based typing

Isolates were characterised using the DNA microarray-based Interarray *S. aureus* kit (fzmb GmbH, Bad Langensalza, Germany). Primer and probe sequences have been previously described in detail^7,8^. Protocols and procedures were in accordance with manufacturer’s instructions. The array covers 336 different targets related to approximately 170 different genes and their allelic variants allowing detection of virulence and resistance factors as well as CC and strain identification based on automated comparison to a database of reference experiments. Briefly, *S. aureus* was cultured on Colombia blood agar. DNA extraction was performed after enzymatic lysis^7,8^. A multiplexed linear amplification was performed using one specific primer per target. During this step, biotin-16-dUTP was randomly incorporated into the amplicons. After incubation and washing, hybridisation was performed to probes immobilised on the array. Hybridisation was detected by streptavidin horseradish peroxidase, generating a localised precipitation of a dye resulting in formation of visible spots. Microarrays were then photographed and analysed with a designated reader (Alere Technologies/Abbott, Jena, Germany) and software. A second microarray (Alere Technologies/Abbott, Jena, Germany^23^) was used on four CC1956 isolates, (WT19, WT63, WT67b, WT68) and one CC49 isolate (WT65) in order to detect additional markers (Supplemental File 2).

### PVL lateral flow test

The expression of PVL was determined by a PVL lateral flow device^21^ (Senova, Weimar, Germany) according to the manufacturer’s instructions.

### Phage induction

Phage preparation was performed as described previously^52^. In summary, cultures were grown in 2×TY medium (prepared using NaCl by Roth, Heidelberg, Germany, as well as tryptone and yeast extract by Becton Dickinson, Le Pont de Claix, France) in an incubator at 30 °C for 18 h overnight. 100 mL of fresh medium was inoculated with 1 mL of the overnight culture and cultured at 37 °C until the middle of the exponential growth phase. mitomycin C (Roche, Basel, Switzerland) was added at a final concentration of 0.5 μg/mL, and cultivation was continued at 30 °C until the OD at 600 nm decreased for the first time (by 0.4 for WT19 and 1.5 for WT65). After centrifugation at 4 °C for 12 min at 3,000 × *g*, 0.1 N NaOH was added to the supernatant until pH = 7.0 was reached. The neutralized supernatant was filtered using a 0.20 µm cellulose acetate (CA) membrane filter (Sartorius, Göttingen, Germany) and the filtrate was stored at 4 °C.

### Phage detection by transmission electron microscopy (TEM)

Suspensions from phage preparations were vortexed briefly. Then, drops of 30 µL were placed on a plate of dental wax. 300 mesh copper grids filmed with formvar, coated with carbon, and hydrophilized by glow discharge (immediately before use) were floated on the drops for 30 min. Then the grids were briefly rinsed in 3 drops of distilled water and the excess liquid was drained on wet filter paper. Finally, one grid of each preparation was contrasted on one drop of 1% phosphotungstic acid and one drop of 1% uranyl acetate for 1 min. The excess contrast medium was drained on wet filter paper. After air drying, the grids were examined by transmission electron microscopy (Tecnai 12, FEI Deutschland GmbH, Dreieich, Germany) at 80 kV. Representative micrographs were taken with a digital camera (TEMCAM FX416, TVIPS, Gauting, Germany).

### Phage DNA Isolation

To sequence pure phage DNA (pDNA), DNA extraction was performed according to the method described previously^53^. The phage filtrate was first treated with 10 µg/mL DNase I (Sigma Aldrich, Steinheim, Germany) and 10 µg/mL RNAse (Qiagen, Hilden, Germany) for 1 h at 37 °C, followed by treatment with 20 mM EDTA, 50 µg/mL proteinase K, and 0.2% SDS and another incubation for 1 h at 65 °C and 300 rpm. A phenol chloroform extraction was performed as described previously^53^. For better separation of the phases, phase lock gel light tubes (Quantabio, Beverly, USA) were used in each step. DNA was concentrated in a SpeedVac vacuum concentrator (Eppendorf, Hamburg, Germany; 25 min at room temperature and 1400 rpm), and the final concentration was measured using the Qubit 4 fluorometer (ThermoFisher Scientific, Waltham, USA).

### Whole-genome sequencing (WGS) by Illumina

All isolates from animals A to G were subjected to whole genome sequencing (WGS) with the Illumina MiSeq platform (Illumina, Inc., San Diego, USA). The DNA was extracted using the QIAamp® DNA Mini Kit (QIAGEN, Hilden, Germany) with adaptations for staphylococci as described previously^54^.

The libraries for WGS were prepared using the Nextera XT DNA Library Preparation Kit (Illumina, Inc., San Diego, USA) according to the manufacturer’s recommendations. The 2 × 300 bp paired-end sequencing in 40-fold multiplexes was performed on the Illumina MiSeq platform (Illumina, Inc., San Diego, USA).

### Oxford Nanopore sequencing

Oxford Nanopore Technology (ONT) sequencing of the isolates WT19, WT65, WT110 and WT111 as well as of the phage DNA prepared from isolate WT19 was performed using two different MinION flow cells (FLO-MIN106D for the isolated bacteria, and two FLO-FLG001 for phage DNA, all containing an R9.4.1 pore). Library preparations were done using the 1D genomic DNA by ligation kit (SQK-LSK109, ONT, Oxford, UK), and the native barcoding expansion kit (EXP-NBD104 and EXP-NBD114, ONT) for the isolated bacteria following manufacturer’s instructions with minor adaptations. In short, prior to the library preparation, an AMPure bead (Agencourt AMPure XP, Beckman Coulter, Krefeld, Germany) clean up step was performed. Potential nicks in DNA and DNA ends were repaired in a combined step using NEBNext FFPE DNA Repair Mix and NEBNext Ultra II End repair/dA-tailing Module (NewEngland Biolabs, Ipswich, USA) by tripling the incubation time. A subsequent second AMPure bead purification was followed by the ligation of sequencing adapters onto prepared ends followed by a clean-up step with AMPure beads. For bacterial DNA, an additional barcoding and clean-up step was performed prior to adapter ligation. Sequencing buffer and loading beads were added to the library. An initial quality check of the flow cells (FLO-MIN106D; ID FAL13739 and FAP37324 as well as FLO-FLG001; ID: AES237) showed around 1289, 509 and 18 active pores, respectively, at the start of sequencing. DNA samples from WT19, WT65, WT110 and WT111 as well as from the WT19 phage preparation were used for loading that comprised a total amount of around 120 ng as measured by Qubit 4. The sequencing ran for 48 h using the MinKNOW software version 20.06.5.

### Sequence assembly and polishing

The 300 bp paired-end reads of all eleven isolates generated by Illumina MiSeq sequencer were de novo assembled into contigs with a minimum size of 200 bp using Unicycler v0.4.7^55^ at default settings.

For all nanopore data sets (bacterial isolates as well as phage DNA from isolate WT19), the guppy basecaller (v4.2.2, ONT) translated and trimmed the MinION raw data (fast5) into quality tagged sequence reads (4,000 reads per fastq-file). To get a smaller and better subset of reads Filtlong (v0.2.0) was used only for bacteria DNA with a median read quality of 14 and a minimum read length of 1,000 bp. The reads of the phage WT19 sequence run were not filtered by Filtlong. The median read quality of 12.5 and a N50 read length of 340 bp was highly suitable for assembly. Flye (v2.8.3) was used to assemble the reads to high quality contigs. Then, a racon-medaka (4-times racon v1.4.3; 1-time medaka v1.2.0) pipeline was applied for polishing. Besides that, sequences were additionally polished by pilon (v1.23) using Illumina sequence data. The NCBI Prokaryotic Genome Annotation Pipeline (PGAP version 2021–01-11.build 5132) was used for annotation of all assembled contigs for the staphylococcal isolates in combination with an in-house database of published staphylococcal sequences.

### Ethics

No animal experiments were performed, and no animal was purposefully killed for this study. Isolates originated from samples which have been submitted to the Leibniz Institute for Zoo and Wildlife Research, Berlin, or to the University of Veterinary Medicine, Vienna, for routine diagnostics and wildlife surveillance.

## Supplementary Information

Supplementary Information.

## Data Availability

Accession numbers are FU-Berlin_WT19, CP084892 and FU-Berlin_WT65, CP084107. The BioProject accession number for the strains sequenced within this project is PRJNA763345. All other data are provided within the manuscript, or as Supplemental Files.
