# Supplementary material for: Staphylococcus aureus isolates from Eurasian Beavers (Castor fiber) carry a novel phage-borne bicomponent leukocidin related to the Panton-Valentine leukocidin
Source: Sci Rep. 2021 Dec 22;11:24394. doi: 10.1038/s41598-021-03823-6 (PMC8695587; doi:10.1038/s41598-021-03823-6)
Supplement: Supplementary file 1 — Supplementary Information. [file 41598_2021_3823_MOESM1_ESM.zip › Supplement 0_Title Page and Legends to Supplemental Files.docx]

# *Staphylococcus aureus* isolates from Eurasian Beavers (*Castor fiber*) carry a novel phage-borne bicomponent leukocidin related to the Panton-Valentine leukocidin

**Stefan Monecke^1,2,3^*, Andrea T. Feßler^4^*, Sindy Burgold-Voigt^1,2^*, Henrike Krüger^4^, Kristin Mühldorfer^5^, Gudrun Wibbelt^5^, Elisabeth M. Liebler-Tenorio^6^, Martin Reinicke^1,2^, Sascha D. Braun^1,2^, Dennis Hanke^4^, Celia Diezel^1,2^, Elke Müller^1,2^, Igor Loncaric^7^, Stefan Schwarz^4#^, Ralf Ehricht^1,2,8#^**

^1^ Leibniz Institute of Photonic Technology (IPHT), Jena, Germany

^2^ InfectoGnostics Research Campus, Jena, Germany

^3^ Institute for Medical Microbiology and Virology, Dresden University Hospital, Dresden, Germany

^4^ Institute of Microbiology and Epizootics, Freie Universität Berlin, Berlin, Germany

^5^ Leibniz Institute for Zoo and Wildlife Research, Department of Wildlife Diseases, Berlin, Germany

^6^ Friedrich-Loeffler-Institut (Federal Research Institute for Animal Health), Institute of Molecular Pathogenesis, Jena, Germany

^7^ Institute of Microbiology, University of Veterinary Medicine, Vienna, Austria.

^8^ Institute of Physical Chemistry, Friedrich-Schiller University, Jena, Germany

Corresponding author: Stefan Monecke, stefan.monecke@leibniz-ipht.de

* These authors have contributed equally to this work and share first authorship.

# These authors share senior authorship.

# Supplementary Material

**Supplemental file 1:** *S. aureus* in different animal species (pdf).

**Supplemental File 2:** Array profiles of study strains and predicted profiles for reference sequences (pdf).

**Supplemental File 3a:** Results of the antimicrobial susceptibility testing of 13 *S. aureus* isolates (animals A-G).

**Supplemental File 3b:** Biocide MICs of 13 *S. aureus* isolates (animals A-G) (pdf).

**Supplemental File 4a:** Percentages of homology between nucleotide sequences of the *lukF* genes and between the amino acid sequences of their gene products of the *S. aureus* and *S. pseudintermedius* strains shown in the alignments in Figure 2.

**Supplemental File 4b:** Percentages of homology between nucleotide sequences of the *lukS* genes and between the amino acid sequences of their gene products of the *S. aureus* and S*. pseudintermedius* strains shown in the alignments in Figure 3.

**Supplemental File 5:** Nucleotide and amino acid sequences of *agrA* of WT19 and WT110 as well as of the BVL- and haemolysis-negative isolate WT111.

**Supplemental File 6a:** Genome sequence of the CC1956 isolate WT19 (fasta).

**Supplemental File 6b**: Genes identified in the genome sequence of the CC1956 isolate WT19 (fasta).

**Supplemental File 7:** Genes located in the *lukF/S*-BV prophages of WT19 and WT65 (pdf).

**Supplemental File 8:** Sequence from the phage preparation from WT19 aligned to the prophage sequence from the isolate´s genome (fasta).

**Supplemental File 9a:** Genome sequence of the CC49 isolate WT65 (fasta).

**Supplemental File 9b:** Genes identified in the genome sequence of the CC49 isolate WT65 (fasta).
