# Supplementary material for: Staphylococcus aureus isolates from Eurasian Beavers (Castor fiber) carry a novel phage-borne bicomponent leukocidin related to the Panton-Valentine leukocidin
Source: Sci Rep. 2021 Dec 22;11:24394. doi: 10.1038/s41598-021-03823-6 (PMC8695587; doi:10.1038/s41598-021-03823-6)
Supplement: Supplementary file 1 — Supplementary Information. [file 41598_2021_3823_MOESM1_ESM.zip › Supplemental File 1_S aureus in different animal species.pdf]

Supplemental file 1: *S. aureus* in different animal species.

S. aureus was also found in apparently any mammalian species yet investigated including cattle and buffalos [1], goats [2-5], ibexes [6], wild [7,8] and domestic sheep [9-11], cervids [8,12-14], domestic swine [15-19] and wild boars [20], camels [21,22], horses [23-26], rhinoceroses [27] and elephants [28,29], domestic rabbits [30], wild rabbits [31] and hares [8,32], beavers [33], ground squirrels [34], red squirrels [35,36], laboratory mice [37], wood mice [38] and voles [8], chinchillas and guinea-pigs [39], hedgehogs [40-43], bats [39,44], domestic dogs and cats [4,26,45-48], foxes [33], mink [49], lynx, wild cats and badgers [8], raccoons [33], bears [50], different species of seals [51-53] and cetaceans [52-57], chimpanzees [58], macaques [59-61], squirrel monkeys [62], wallabies [63] and opossums [64].

S. aureus has been found in a variety of birds that include domestic chicken, turkeys, quails, various species of raptors, storks and ibises, parrots, a species of bustard, wildfowl including ducks and swans, gulls, magpies, crows and rooks and finches [8,13,39,65-85].

It was also detected in the saliva of Comodo dragons [86].

References:

1 El-Ashker, M. *et al.* Staphylococci in cattle and buffaloes with mastitis in Dakahlia Governorate, Egypt. *J Dairy Science* **98**, 7450-7459, doi:10.3168/jds.2015-9432 (2015).

2 Bar-Gal, G. K. *et al.* Host-specificity of *Staphylococcus aureus* causing intramammary infections in dairy animals assessed by genotyping and virulence genes. *Vet Microbiol* **176**, 143-154, doi:10.1016/j.vetmic.2015.01.007 (2015).

3 Eriksson, J., Espinosa-Gongora, C., Stamphøj, I., Larsen, A. R. & Guardabassi, L. Carriage frequency, diversity and methicillin resistance of *Staphylococcus aureus* in Danish small ruminants. *Vet Microbiol* **163**, 110-115, doi:10.1016/j.vetmic.2012.12.006 (2013).

4 Gharsa, H. *et al.* Molecular Characterization of *Staphylococcus aureus* from Nasal Samples of Healthy Farm Animals and Pets in Tunisia. *Vector-Borne Zoonotic Dis* **15**, 109-115, doi:10.1089/vbz.2014.1655 (2015).

5 Rainard, P., Corrales, J. C., Barrio, M. B., Cochard, T. & Poutrel, B. Leucotoxic activities of *Staphylococcus aureus* strains isolated from cows, ewes, and goats with mastitis: importance of LukM/LukF'-PV leukotoxin. *Clin Diagn Lab Immunol* **10**, 272-277, doi:10.1128/cdli.10.2.272-277.2003 (2003).

6 Gonzalez-Candela, M., Cubero-Pablo, M. J., Martin-Atance, P. & Leon-Vizcaino, L. Potential pathogens carried by Spanish ibex (*Ca*pra pyrenaica hispanica) in southern Spain. *J Wildl Dis* **42**, 325-334, doi:10.7589/0090-3558-42.2.325 (2006).

7 Marshall, M. M., Songer, J. G., Chilelli, C. J. & deVos, J. C. Isolations of aerobic bacteria from wild desert bighorn sheep (*Ovis canadensis nelsoni* and *O. c. mexicana*) in Arizona. *J Wildl Dis* **19**, 98-100, doi:10.7589/0090-3558-19.2.98 (1983).

8 Monecke, S. *et al.* Diversity of *Staphylococcus aureus* Isolates in European Wildlife. *PLOS ONE* **11**, e0168433, doi:10.1371/journal.pone.0168433 (2016).

9 Bath, G. F., Janse van Rensburg, A., Pettey, K. P., van Vuuren, M. & Kidanemariam, A. A literature review and investigation of staphylococcal necrotic dermatitis in sheep. *J S Afr Vet Assoc* **82**, 227-231, doi:https://doi.org/10.4102/jsava.v82i4.79 (2011).

10 Elbir, H. *et al.* Ovine clone ST1464: a predominant genotype of *Staphylococcus aureus* subsp. anaerobius isolated from sheep in Sudan. *J Infect Dev Ctries* **4**, 235-238, doi:10.3855/jidc.632 (2010).

11 Gharsa, H. *et al.* Prevalence, antibiotic resistance, virulence traits and genetic lineages of *Staphylococcus aureus* in healthy sheep in Tunisia. *Vet Microbiol* **156**, 367-373, doi:10.1016/j.vetmic.2011.11.009 (2012).

12 Luzzago, C. *et al.* Clonal diversity, virulence-associated genes and antimicrobial resistance profile of *Staphylococcus aureus* isolates from nasal cavities and soft tissue infections in wild ruminants in Italian Alps. *Vet Microbiol* **170**, 157-161, doi:10.1016/j.vetmic.2014.01.016 (2014).

13 Porrero, M. C. *et al.* Carriage of *Staphylococcus aureus* by Free-Living Wild Animals in Spain. *Appl Environ Microbiol* **80**, 4865-4870, doi:10.1128/aem.00647-14 (2014).

14 Porrero, M. C. *et al.* Methicillin resistant *Staphylococcus aureus* (MRSA) carriage in different free-living wild animal species in Spain. *Vet J* **198**, 127-130, doi:10.1016/j.tvjl.2013.06.004 (2013).

15 Neela, V. *et al.* Prevalence of ST9 methicillin-resistant *Staphylococcus aureus* among pigs and pig handlers in Malaysia. *J Clin Microbiol* **47**, 4138-4140, doi:10.1128/JCM.01363-09 (2009).

16 Cui, S. *et al.* Isolation and characterization of methicillin-resistant *Staphylococcus aureus* from swine and workers in China. *J Antimicrob Chemother* **64**, 680-683, doi:10.1093/jac/dkp275 (2009).

17 van Duijkeren, E. *et al.* Transmission of methicillin-resistant *Staphylococcus aureus* strains between different kinds of pig farms. *Vet Microbiol* **126**, 383-389, doi:10.1016/j.vetmic.2007.07.021 (2008).

18 de Neeling, A. J. *et al.* High prevalence of methicillin resistant *Staphylococcus aureus* in pigs. *Vet Microbiol* **122**, 366-372, doi:10.1016/j.vetmic.2007.01.027 (2007).

19 Armand-Lefevre, L., Ruimy, R. & Andremont, A. Clonal comparison of *Staphylococcus aureus* isolates from healthy pig farmers, human controls, and pigs. *Emerg Infect Dis* **11**, 711-714, doi:https://doi.org/10.3201/eid1105.040866 (2005).

20 Meemken, D. *et al.* Genotypic and Phenotypic Characterization of *Staphylococcus aureus* Isolates from Wild Boars. *Appl Environ Microbiol* **79**, 1739-1742, doi:10.1128/AEM.03189-12 (2013).

21 Shuiep, E. S. *et al.* Phenotypic and genotypic characterization of *Staphylococcus aureus* isolated from raw camel milk samples. *Res Vet Sci* **86**, 211-215, doi:10.1016/j.rvsc.2008.07.011 (2009).

22 Monecke, S. *et al.* Microarray-based genotyping of *Staphylococcus aureus* isolates from camels. *Vet Microbiol* **150**, 309-314, doi:10.1016/j.vetmic.2011.02.001 (2011).

23 Weese, J. S. *et al.* Methicillin-resistant *Staphylococcus aureus* in horses and horse personnel, 2000-2002. *Emerg Infect Dis* **11**, 430-435, doi:10.3201/eid1103.040481 (2005).

24 Cuny, C. *et al.* Emergence of MRSA infections in horses in a veterinary hospital: strain characterisation and comparison with MRSA from humans. *Euro Surveill* **11**, 44-47 (2006).

25 Walther, B. *et al.* Comparative molecular analysis substantiates zoonotic potential of equine methicillin-resistant *Staphylococcus aureus. J Clin Microbiol* **47**, 704-710, doi:10.1128/JCM.01626-08 (2009).

26 Abbott, Y., Leggett, B., Rossney, A. S., Leonard, F. C. & Markey, B. K. Isolation rates of meticillin-resistant *Staphylococcus aureus* in dogs, cats and horses in Ireland. *Veterinary Record* **166**, 451-455, doi:10.1136/vr.b4814 (2010).

27 Clausen, B. & Ashford, W. A. Bacteriologic survey of black rhinoceros (*Diceros bicornis*). *J Wildl Dis* **16**, 475-480, doi:10.7589/0090-3558-16.4.475 (1980).

28 Centers for Disease, C. & Prevention. Methicillin-resistant *Staphylococcus aureus* skin infections from an elephant calf--San Diego, California, 2008. *MMWR Morb Mortal Wkly Rep* **58**, 194-198 (2009).

29 Kodikara, D. S., De Silva, N., Makuloluwa, C. A., De Silva, N. & Gunatilake, M. Bacterial and fungal pathogens isolated from corneal ulcerations in domesticated elephants (*Elephas maximus maximus*) in Sri Lanka. *Vet Ophthalmol* **2**, 191-192, doi:10.1046/j.1463-5224.1999.00072.x (1999).

30 Vancraeynest, D. *et al.* International dissemination of a high virulence rabbit *Staphylococcus aureus* clone. *J Vet Med B Infect Dis Vet Public Health* **53**, 418-422, doi:10.1111/j.1439-0450.2006.00977.x (2006).

31 Ruiz-Ripa, L. *et al.* Diversity of *Staphylococcus aureus* clones in wild mammals in Aragon, Spain, with detection of MRSA ST130-*mecC* in wild rabbits. *J Appl Microbiol* **127**, 284-291, doi:10.1111/jam.14301 (2019).

32 Loncaric, I. *et al. mecC*- and *mecA*-positive meticillin-resistant *Staphylococcus aureus* (MRSA) isolated from livestock sharing habitat with wildlife previously tested positive for *mecC*-positive MRSA. *Vet Dermatol* **25**, 147-148, doi:10.1111/vde.12116 (2014).

33 Plommet, M. G. & Wilson, J. B. Serological typing of *Staphylococcus aureus* from wild animals. *J Comp Pathol* **79**, 425-433, doi:10.1016/0021-9975(69)90062-0 (1969).

34 Campbell, G. A., Kosanke, S. D., Toth, D. M. & White, G. L. Disseminated staphylococcal infection in a colony of captive ground squirrels (*Citellus lateralis*). *J Wildl Dis* **17**, 177-181, doi:10.7589/0090-3558-17.2.177 (1981).

35 Simpson, V. R. *et al.* Association of a *lukM*-positive clone of *Staphylococcus aureus* with fatal exudative dermatitis in red squirrels (*Sciurus vulgaris*). *Vet Microbiol* **162**, 987-991, doi:10.1016/j.vetmic.2012.10.025 (2013).

36 Simpson, V. R. *et al.* Mortality in red squirrels (*Sciurus vulgaris*) associated with exudative dermatitis. *Veterinary Record* **167**, 59-62, doi:10.1136/vr.b4887 (2010).

37 Holtfreter, S. *et al.* Characterization of a mouse-adapted *Staphylococcus aureus* strain. *PLoS ONE* **8**, e71142, doi:10.1371/journal.pone.0071142 (2013).

38 Gómez, P. *et al.* Detection of methicillin-resistant *Staphylococcus aureus* (MRSA) carrying the *mecC* gene in wild small mammals in Spain. *J Antimicrob Chemother* **69**, 2061-2064, doi:10.1093/jac/dku100 (2014).

39 Walther, B. *et al.* Methicillin-resistant *Staphylococcus aureus* (MRSA) isolated from small and exotic animals at a university hospital during routine microbiological examinations. *Vet Microbiol* **127**, 171-178, doi:10.1016/j.vetmic.2007.07.018 (2008).

40 Smith, J. M. *Staphylococcus aureus* Strains Associated with the Hedgehog, *Erinaceus Europaeus. J Hyg (Lond)* **63**, 285-291, doi:10.1017/s0022172400045162 (1965).

41 Monecke, S. *et al.* Detection of *mecC*-positive *Staphylococcus aureus* (CC130-MRSA-XI) in diseased European hedgehogs (*Erinaceus europaeus*) in Sweden. *PLoS ONE* **8**, e66166, doi:10.1371/journal.pone.0066166 (2013).

42 Rasmussen, S. L. *et al.* European hedgehogs (*Erinaceus europaeus*) as a natural reservoir of methicillin-resistant *Staphylococcus aureus* carrying *mecC* in Denmark. *PLoS ONE* **14**, e0222031, doi:10.1371/journal.pone.0222031 (2019).

43 Bengtsson, B. *et al.* High occurrence of *mecC*-MRSA in wild hedgehogs (*Erinaceus europaeus*) in Sweden. *Vet Microbiol* **207**, 103-107, doi:10.1016/j.vetmic.2017.06.004 (2017).

44 Akobi, B., Aboderin, O., Sasaki, T. & Shittu, A. Characterization of *Staphylococcus aureus* isolates from faecal samples of the Straw-Coloured Fruit Bat (*Eidolon helvum*) in Obafemi Awolowo University (OAU), Nigeria. *BMC Microbiol* **12**, 279, doi:10.1186/1471-2180-12-279 (2012).

45 Weese, J. S. *et al.* Suspected transmission of methicillin-resistant *Staphylococcus aureus* between domestic pets and humans in veterinary clinics and in the household. *Vet Microbiol* **115**, 148-155, doi:10.1016/j.vetmic.2006.01.004 (2006).

46 Abdel-moein, K. A. & Samir, A. Isolation of enterotoxigenic *Staphylococcus aureus* from pet dogs and cats: a public health implication. *Vector Borne Zoonotic Dis* **11**, 627-629, doi:10.1089/vbz.2010.0272 (2011).

47 Abdel-moein, K. A., El-Hariri, M. & Samir, A. Methicillin-resistant *Staphylococcus aureus*: an emerging pathogen of pets in Egypt with a public health burden. *Transbound Emerg Dis* **59**, 331-335, doi:10.1111/j.1865-1682.2011.01273.x (2012).

48 Sing , A., Tuschak , C. & Hörmansdorfer , S. Methicillin-Resistant *Staphylococcus aureus* in a Family and Its Pet Cat. *New England J Medicine* **358**, 1200-1201, doi:10.1056/NEJMc0706805 (2008).

49 Ryan, M. J., O'Connor, D. J. & Nielsen, S. W. *Staphylococcus aureus* mastitis in nursing mink affected with aleutian disease. *J Wildl Dis* **15**, 533-535, doi:10.7589/0090-3558-15.4.533 (1979).

50 McBurney, S., Veitch, A. M. & Daoust, P. Y. Bacterial valvular endocarditis in a black bear from Labrador. *J Wildl Dis* **36**, 788-791, doi:10.7589/0090-3558-36.4.788 (2000).

51 Thornton, S. M., Nolan, S. & Gulland, F. M. Bacterial isolates from California sea lions (*Zalophus californianus*), harbor seals (*Phoca vitulina*), and northern elephant seals (*Mirounga angustirostris*) admitted to a rehabilitation center along the central California coast, 1994-1995. *J Zoo Wildl Med* **29**, 171-176 (1998).

52 van Elk, C. E., Boelens, H. A. M., van Belkum, A., Foster, G. & Kuiken, T. Indications for both host-specific and introduced genotypes of *Staphylococcus aureus* in marine mammals. *Vet Microbiol* **156**, 343-346, doi:10.1016/j.vetmic.2011.10.034 (2012).

53 Faires, M. C., Gehring, E., Mergl, J. & Weese, J. S. Methicillin-resistant *Staphylococcus aureus* in marine mammals. *Emerg Infect Dis [serial on the Internet]*, DOI: 10.3201/eid1512.090220 (2009).

54 Siebert, U. *et al.* Pyogranulomatous myocarditis due to *Staphylococcus aureus* septicaemia in two harbour porpoises (*Phocoena phocoena*). *Vet Rec* **150**, 273-277, doi:10.1136/vr.150.9.273 (2002).

55 Colgrove, G. S. & Migaki, G. Cerebral abscess associated with stranding in a dolphin. *J Wildl Dis* **12**, 271-274, doi:10.7589/0090-3558-12.2.271 (1976).

56 Power, E. & Murphy, S. *Staphylococcus aureus* septicaemia in a killer whale. *Vet Rec* **150**, 819 (2002).

57 Hower, S. *et al.* Clonally related methicillin-resistant *Staphylococcus aureus* isolated from short-finned pilot whales (*Globicephala macrorhynchus*), human volunteers, and a bayfront cetacean rehabilitation facility. *Microb Ecol* **65**, 1024-1038, doi:10.1007/s00248-013-0178-3 (2013).

58 Schaumburg, F. *et al.* Highly divergent *Staphylococcus aureus* isolates from African non-human primates. *Environ Microbiol Rep* **4**, 141-146, doi:10.1111/j.1758-2229.2011.00316.x (2012).

59 Roberts, M. C. *et al. Staphylococcus aureus* and Methicillin Resistant *S. aureus* in Nepalese Primates: Resistance to Antimicrobials, Virulence, and Genetic Lineages. *Antibiotics (Basel)* **9**, doi:10.3390/antibiotics9100689 (2020).

60 Roberts, M. C. *et al.* The human clone ST22 SCCmec IV methicillin-resistant *Staphylococcus aureus* isolated from swine herds and wild primates in Nepal: is man the common source? *FEMS Microbiol Ecol* **94**, doi:10.1093/femsec/fiy052 (2018).

61 Roberts, M. C. *et al.* Molecular Analysis of Two Different MRSA Clones ST188 and ST3268 From Primates (*Macaca spp.*) in a United States Primate Center. *Front Microbiol* **9**, doi:10.3389/fmicb.2018.02199 (2018).

62 Drougka, E. *et al.* Human *Staphylococcus aureus* lineages among Zoological Park residents in Greece. *Open Veterinary J* **5**, 148-153 (2015).

63 Chen, M. M. S., Monecke, S. & Brown, M. H. Clonal diversity of methicillin-sensitive *Staphylococcus aureus* from South Australian wallabies. *One Health*, doi:10.1016/j.onehlt.2015.12.001 (2016).

64 Siqueira, D. B. *et al. Staphylococcus aureus* Mastitis in a White-Eared Opossum (*Didelphis albiventris*) in the Atlantic Forest of Northeast Brazil. *J Zoo Wildlife Medicine* **41**, 526-529, doi:10.1638/2009-0079.1 (2010).

65 Lowder, B. V. *et al.* Recent human-to-poultry host jump, adaptation, and pandemic spread of *Staphylococcus aureus*. *Proc Natl Acad Sci U S A* **106**, 19545-19550, doi:10.1073/pnas.0909285106 (2009).

66 Feßler, A. T. *et al.* Characterization of methicillin-resistant *Staphylococcus aureus* isolates from food and food products of poultry origin in Germany. *Appl Environ Microbiol* **77**, 7151-7157, doi:10.1128/AEM.00561-11 (2011).

67 Ebner, R., Johler, S., Sihto, H.-M., Stephan, R. & Zweifel, C. Microarray-Based Characterization of *Staphylococcus aureus* Isolates Obtained from Chicken Carcasses. *J Food Protection* **76**, 1471-1474, doi:10.4315/0362-028x.jfp-13-009 (2013).

68 Monecke, S. *et al.* Genotyping of *Staphylococcus aureus* isolates from diseased poultry. *Vet Microbiol* **162**, 806-812, doi:10.1016/j.vetmic.2012.10.018 (2013).

69 Schaumburg, F. *et al.* The risk to import ESBL-producing Enterobacteriaceae and *Staphylococcus aureus* through chicken meat trade in Gabon. *BMC Microbiol* **14**, 286, doi:10.1186/s12866-014-0286-3 (2014).

70 Linares, J. A. & Wigle, W. L. *Staphylococcus aureus* pneumonia in turkey poults with gross lesions resembling aspergillosis. *Avian Dis* **45**, 1068-1072 (2001).

71 Laukova, A., Marounek, M. & Bod'a, K. Characteristics of enterococci and staphylococci isolated from the crop and caecum of Japanese quails exposed to microgravity conditions. *Vet Med (Praha)* **40**, 317-321 (1995).

72 Pyzik, E. & Marek, A. Characterization of bacteria of the genus *Staphylococcus* isolated from the eggs of Japanese quail (*Coturnix coturnix japonica*). *Pol J Vet Sci* **15**, 767-772 (2012).

73 Gómez, P. *et al.* Detection of MRSA ST3061-t843-*mecC* and ST398-t011-*mecA* in white stork nestlings exposed to human residues. *J Antimicrob Chemother* **71**, 53-57, doi:10.1093/jac/dkv314 (2016).

74 Wobeser, G. & Kost, W. Starvation, staphylococcosis, and vitamin A deficiency among mallards overwintering in Saskatchewan. *J Wildl Dis* **28**, 215-222, doi:https://doi.org/10.7589/0090-3558-28.2.215 (1992).

75 Sousa, M. *et al.* Antimicrobial resistance determinants in *Staphylococcus spp.* recovered from birds of prey in Portugal. *Vet Microbiol* **171**, 436-440, doi:10.1016/j.vetmic.2014.02.034 (2014).

76 Silvanose, C. D., Bailey, T. A., Samour, J. H. & Naldo, J. L. Intestinal protozoa and associated bacteria in captive houbara bustards (*Chlamydotis undulata*) in the United Arab Emirates. *Avian Pathol* **28**, 94-97, doi:10.1080/03079459995109 (1999).

77 Silvanose, C. D., Bailey, T. A., Naldo, J. L. & Howlett, J. C. Bacterial flora of the conjunctiva and nasal cavity in normal and diseased captive bustards. *Avian Dis* **45**, 447-451 (2001).

78 Hájek, V. & Balusek, J. Biochemical properties and differentiation of coagulase-positive staphylococci from rooks and gulls. *Res Vet Sci* **44**, 242-246 (1988).

79 Briscoe, J. A., Morris, D. O., Rankin, S. C., Hendrick, M. J. & Rosenthal, K. L. Methicillin-resistant Staphylococcus aureus-associated dermatitis in a Congo African grey parrot (*Psittacus erithacus erithacus*). *J Avian Med Surg* **22**, 336-343, doi:https://doi.org/10.1647/2008-014.1 (2008).

80 Losito, P., Vergara, A., Muscariello l, T. & Ianieri, A. Antimicrobial susceptibility of environmental *Staphylococcus aureus* strains isolated from a pigeon slaughterhouse in Italy. *Poult Sci* **84**, 1802-1807, doi:10.1093/ps/84.11.1802 (2005).

81 Hukkanen, R. R., Richardson, M., Wingfield, J. C., Treuting, P. & Brabb, T. Avipox sp. in a colony of gray-crowned rosy finches (*Leucosticte tephrocotis*). *Comp Med* **53**, 548-552 (2003).

82 Prattis, S. M., Cioffee, C. J., Reinhard, G. & Zaoutis, T. E. A retrospective study of disease and mortality in zebra finches. *Lab Anim Sci* **40**, 402-405 (1990).

83 Paterson, G. K. *et al.* The newly described *mecA* homologue, *mec*ALGA251, is present in methicillin-resistant *Staphylococcus aureus* isolates from a diverse range of host species. *J Antimicrob Chemother* **67**, 2809-2813, doi:10.1093/jac/dks329 (2012).

84 Loncaric, I. *et al.* Comparison of ESBL--and AmpC producing Enterobacteriaceae and methicillin-resistant *Staphylococcus aureus* (MRSA) isolated from migratory and resident population of rooks (*Corvus frugilegus*) in Austria. *PLoS ONE* **8**, e84048, doi:10.1371/journal.pone.0084048 (2013).

85 Ruiz-Ripa, L. *et al.* Detection of MRSA of Lineages CC130-*mecC* and CC398-*mecA* and S*taphylococcus delphini-lnu*(A) in Magpies and Cinereous Vultures in Spain. *Microb Ecol* **78**, 409-415, doi:10.1007/s00248-019-01328-4 (2019).

86 Montgomery, J., Gillespie, D., Sastrawan, P., Fredeking, T. & Stewart, G. Aerobic salivary bacteria in wild and captive Komodo dragons. *J Wildl Dis* **38**, 545-551, doi:https://doi.org/10.7589/0090-3558-38.3.545 (2002).
