# Supplementary material for: Staphylococcus aureus isolates from Eurasian Beavers (Castor fiber) carry a novel phage-borne bicomponent leukocidin related to the Panton-Valentine leukocidin
Source: Sci Rep. 2021 Dec 22;11:24394. doi: 10.1038/s41598-021-03823-6 (PMC8695587; doi:10.1038/s41598-021-03823-6)
Supplement: Supplementary file 1 — Supplementary Information. [file 41598_2021_3823_MOESM1_ESM.zip › Supplemental File 2_Array profiles.pdf]

			TYPING DATA			SPECIES MARKER							STAPHYLOXANTHIN BIOSYNTHESIS OPERON				
													crtM				
STRAIN / ISOLATE	GenBank	SAMEA	RIDOM spa typing SHORT	RIDOM spa typing FULL	MLST	rrnD1	gapA	katA	CoA	nuc1	spa	sbi	crtM-nonST93	crtM-ST93	crtN	crtO	crtP
						Domain 1 of 23S-rRNA	glyceraldehyde 3-phosphate dehydrogenase, locus 1	katalase A	coagulase	thermostable extracellular nuclease	staphylococcal protein A	IgG-binding protein	dehydrosqualene synthase		dehydrosqualene desaturase	staphyloxanthin acyltransferase	diaponeurosporene oxidase
**>CC8-MSSA**																	
Strain NCTC8325: in silico predicted hybridisation pattern	Genbank CP000253.1		t211	11-19-12-12-21-17-34-24-34-22-25	ST0008	POS	POS	POS	POS	POS	POS	POS	POS	NEG	POS	POS	POS
Strain RN4220:VC40: in silico predicted hybridisation pattern	Genbank CP003033.1	SAMN02603393	t211	11-19-12-12-21-17-34-24-34-22-25	ST8	POS	POS	POS	POS	POS	POS	POS	POS	NEG	POS	POS	POS
Strain Newman: in silico predicted hybridisation pattern	Genbank AP009351.1	SAMD00060913	t008	11-19-12-21-17-34-24-34-22-25	ST8	POS	POS	POS	POS	POS	POS	POS	POS	NEG	POS	POS	POS
Beaver I_Austria_B2						POS	POS	POS	POS	POS	POS	POS					
Beaver J_Austria_B3						POS	POS	POS	POS	POS	POS	POS					
**>CC12-MSSA**																	
Strain KLT6: in silico predicted hybridisation pattern	Genbank APFH	SAMN02472075	t160	07-23-21-24-33-22-17	ST0012	POS	POS	POS	POS	POS	POS	POS	POS	NEG	POS	POS	POS
Strain 21266: in silico predicted hybridisation pattern	Genbank AFTT	SAMN00116895	t160	07-23-21-24-33-22-17	ST1461(1MM)	POS	POS	POS	POS	POS	POS	POS	POS	NEG	POS	POS	POS
NP66 chromosome: in silico predicted hybridisation pattern	Genbank CP041037	SAMN12046023			ST12	POS	POS	POS	POS	POS	POS	POS	POS	NEG	POS	POS	POS
Beaver K_Austria_B4						POS	POS	POS	POS	POS	POS	POS					
**>CC49-MSSA**																	
Strain 21262: in silico predicted hybridisation pattern	Genbank AHJW	SAMN00116870	incomplete		ST49	POS	POS	POS	POS	POS	POS	POS	POS	NEG	POS	POS	POS
Strain Tager 104: in silico predicted hybridisation pattern	Genbank AVBR	SAMN02393830	t208	04-20-17-17-31-31-24-17-17-17-17-25	ST49	POS	POS	POS	POS	POS	POS	POS	POS	NEG	POS	POS	POS
**>CC49-MSSA (lukF-P83/lukM+)**																	
Strain 22_M61_07_10: in silico predicted hybridisation pattern		SAMEA1484814			ST49	POS	POS	POS	POS	POS	POS	POS	POS	NEG	POS	POS	POS
Strain 22_M48_10_10: in silico predicted hybridisation pattern		SAMEA1484826			ST1957	POS	POS	POS	POS	POS	POS	POS	POS	NEG	POS	POS	POS
Strain 22_M47_10_10: in silico predicted hybridisation pattern		SAMEA1484828			ST1957	POS	POS	POS	POS	POS	POS	POS	POS	NEG	POS	POS	POS
**>CC49-MSSA (lukF-PV+/lukS-PV?)**																	
Beaver C_Bavaria_WT65: in silico predicted hybridisation pattern			t208	04-20-17-17-31-31-24-17-17-17-17-25	ST49	POS	POS	POS	POS	POS	POS	POS	POS	NEG	POS	POS	POS
Beaver C_Bavaria_WT65			t208	04-20-17-17-31-31-24-17-17-17-17-25	ST49	POS	POS	POS	POS	POS	POS	POS	POS	NEG	POS	POS	POS
**>CC398-MSSA**																	
Strain 21331: in silico predicted hybridisation pattern	Genbank AGTV	SAMN00116844	t571	08-16-02-25-02-25-34-25	ST398	POS	POS	POS	POS	POS	POS	POS	POS	NEG	POS	POS	POS
Strain 71193: in silico predicted hybridisation pattern	Genbank CP003045.1	SAMN02603419	t571	08-16-02-25-02-25-34-25	ST398	POS	POS	POS	POS	POS	POS	POS	POS	NEG	POS	POS	POS
Strain S123: in silico predicted hybridisation pattern	Genbank AUPU	SAMN02469811	t011	08-16-02-25-34-24-25	ST398	POS	POS	POS	POS	POS	POS	POS	POS	NEG	POS	POS	POS
Beaver H_Austria_B1						POS	POS	POS	POS	POS	POS	POS					
**>CC1956-MSSA**																	
Strain C6589: in silico predicted hybridisation pattern		SAMEA3251370			ST1956	POS	POS	POS	POS	POS	POS	POS	POS	NEG	POS	POS	POS
Strain C6591: in silico predicted hybridisation pattern		SAMEA3251372			ST1956	POS	POS	POS	POS	POS	POS	POS	POS	NEG	POS	POS	POS
Strain C6597: in silico predicted hybridisation pattern		SAMEA3251376			ST2766	POS	POS	POS	POS	POS	POS	POS	POS	NEG	POS	POS	POS
Strain C6598: in silico predicted hybridisation pattern		SAMEA3251377			ST1956	POS	POS	POS	POS	POS	POS	POS	POS	NEG	POS	POS	POS
Strain C6601: in silico predicted hybridisation pattern		SAMEA3251380			ST1956	POS	POS	POS	POS	POS	POS	POS	POS	NEG	POS	POS	POS
**>CC1956-MSSA (lukF-PV+/lukS-PV?)**																	
Beaver A_Berlin_WT19: in silico predicted hybridisation pattern			t3058	14-44-34-17-34-50-17	ST4614	POS	POS	POS	POS	POS	POS	POS	POS	NEG	POS	POS	POS
Beaver A_Berlin_WT19			t3058	14-44-34-17-34-50-17	ST4614	POS	POS	POS	POS	POS	POS	POS	POS	NEG	POS	POS	POS
Beaver B_Berlin_WT63			t3058	14-44-34-17-34-50-17	ST4614	POS	POS	POS	POS	POS	POS	POS	POS	NEG	POS	POS	POS
Beaver B_Berlin_WT64			t3058	14-44-34-17-34-50-17	ST4614	POS	POS	POS	POS	POS	POS	POS					
Beaver D_Berlin_WT66			t3058	14-44-34-17-34-50-17	ST4614	POS	POS	POS	POS	POS	POS	POS					
Beaver D_Berlin_WT67A			t3058	14-44-34-17-34-50-17	ST4614	POS	POS	POS	POS	POS	POS	POS					
Beaver D_Berlin_WT67B			t3058	14-44-34-17-34-50-17	ST4614	POS	POS	POS	POS	POS	POS	POS	POS	NEG	POS	POS	POS
Beaver D_Berlin_WT68			t3058	14-44-34-17-34-50-17	ST4614	POS	POS	POS	POS	POS	POS	POS	POS	NEG	POS	POS	POS
Beaver D_Berlin_WT69			t3058	14-44-34-17-34-50-17	ST4614	POS	POS	POS	POS	POS	POS	POS					
Beaver F_Berlin_WT71			t3058	14-44-34-17-34-50-17	ST4614	POS	POS	POS	POS	POS	POS	POS					
Beaver E_Berlin_WT70			t3058	14-44-34-17-34-50-17	ST4614	POS	POS	POS	POS	POS	POS	POS					
Beaver G_Berlin_WT110						POS	POS	POS	POS	POS	POS	POS					
Beaver G_Berlin_WT111						POS	POS	POS	POS	POS	POS	POS					

STRAIN / ISOLATE	REGULATORY GENES																					
	sarA	saeS	vraS	agrI				agrII				agrIII				agrIV			agrV (argenteus)			hld
				agrI (total)	agrB-I	agrC-I	agrD-I	agrII (total)	agrB-II	agrC-II	agrD-II	agrIII (total)	agrB-III	agrC-III	agrD-III	agrIV (total)	agrB-IV	agrC-IV	agrV-ST1850	agrV-ST2198	agrV-other	
	staphylococcal accessory regulator A	histidine protein kinase, sae locus	sensor protein	accessory gene regulator allele I				accessory gene regulator allele II				accessory gene regulator allele III				accessory gene regulator allele IV			accessory gene regulator alleles from S. argenteus group			haemolysin delta
**>CC8-MSSA**																						
Strain NCTC8325: in silico predicted hybridisation pattern	AMB	POS	POS	POS	POS	POS	POS	NEG	NEG	NEG	NEG	NEG	NEG	NEG	NEG	NEG	NEG	NEG	NEG	NEG	NEG	POS
Strain RN4220:VC40: in silico predicted hybridisation pattern	AMB	POS	POS	POS	POS	POS	POS	NEG	NEG	NEG	NEG	NEG	NEG	NEG	NEG	NEG	NEG	NEG	NEG	NEG	NEG	POS
Strain Newman: in silico predicted hybridisation pattern	AMB	POS	POS	POS	POS	POS	POS	NEG	NEG	NEG	NEG	NEG	NEG	NEG	NEG	NEG	NEG	NEG	NEG	NEG	NEG	POS
Beaver I_Austria_B2	POS	POS	POS	POS	POS	POS	POS	NEG	NEG	NEG	NEG	NEG	NEG	NEG	NEG	NEG	POS	NEG				POS
Beaver J_Austria_B3	POS	POS	POS	POS	POS	POS	POS	NEG	NEG	NEG	NEG	NEG	NEG	NEG	NEG	NEG	AMB	NEG				POS
**>CC12-MSSA**																						
Strain KLT6: in silico predicted hybridisation pattern	AMB	POS	POS	NEG	NEG	NEG	NEG	POS	POS	POS	POS	NEG	NEG	NEG	NEG	NEG	NEG	NEG	NEG	NEG	NEG	POS
Strain 21266: in silico predicted hybridisation pattern	AMB	POS	POS	NEG	NEG	NEG	NEG	POS	POS	POS	POS	NEG	NEG	NEG	NEG	NEG	NEG	NEG	NEG	NEG	NEG	POS
NP66 chromosome: in silico predicted hybridisation pattern	POS	POS	POS	NEG	NEG	NEG	NEG	POS	POS	POS	POS	NEG	NEG	NEG	NEG	NEG	NEG	NEG	NEG	NEG	NEG	POS
Beaver K_Austria_B4	POS	POS	POS	NEG	NEG	NEG	NEG	POS	POS	POS	POS	NEG	NEG	NEG	NEG	NEG	NEG	NEG				POS
**>CC49-MSSA**																						
Strain 21262: in silico predicted hybridisation pattern	AMB	POS	POS	NEG	NEG	NEG	NEG	POS	POS	POS	POS	NEG	NEG	NEG	NEG	NEG	NEG	NEG	NEG	NEG	NEG	POS
Strain Tager 104: in silico predicted hybridisation pattern	AMB	POS	POS	NEG	NEG	NEG	NEG	POS	POS	POS	POS	NEG	NEG	NEG	NEG	NEG	NEG	NEG	NEG	NEG	NEG	POS
**>CC49-MSSA (lukF-P83/lukM+)**																						
Strain 22_M61_07_10: in silico predicted hybridisation pattern	POS	POS	POS	NEG	NEG	NEG	NEG	POS	POS	POS	POS	NEG	NEG	NEG	NEG	NEG	NEG	NEG	NEG	NEG	NEG	POS
Strain 22_M48_10_10: in silico predicted hybridisation pattern	POS	POS	POS	NEG	NEG	NEG	NEG	POS	POS	POS	POS	NEG	NEG	NEG	NEG	NEG	NEG	NEG	NEG	NEG	NEG	POS
Strain 22_M47_10_10: in silico predicted hybridisation pattern	POS	POS	POS	NEG	NEG	NEG	NEG	POS	POS	POS	POS	NEG	NEG	NEG	NEG	NEG	NEG	NEG	NEG	NEG	NEG	POS
**>CC49-MSSA (lukF-PV+/lukS-PV?)**																						
Beaver C_Bavaria_WT65: in silico predicted hybridisation pattern	POS	POS	POS	NEG	NEG	NEG	NEG	POS	POS	POS	POS	NEG	NEG	NEG	NEG	NEG	NEG	NEG	NEG	NEG	NEG	POS
Beaver C_Bavaria_WT65	POS	POS	POS	NEG	NEG	NEG	NEG	POS	POS	POS	POS	NEG	NEG	NEG	NEG	NEG	NEG	NEG	NEG	NEG		POS
**>CC398-MSSA**																						
Strain 21331: in silico predicted hybridisation pattern	AMB	POS	POS	AMB	AMB	POS	AMB	NEG	NEG	NEG	NEG	NEG	NEG	NEG	NEG	NEG	NEG	NEG	NEG	NEG	NEG	POS
Strain 71193: in silico predicted hybridisation pattern	AMB	POS	POS	AMB	AMB	POS	AMB	NEG	NEG	NEG	NEG	NEG	NEG	NEG	NEG	NEG	NEG	NEG	NEG	NEG	NEG	POS
Strain S123: in silico predicted hybridisation pattern	AMB	POS	POS	AMB	AMB	POS	AMB	NEG	NEG	NEG	NEG	NEG	NEG	NEG	NEG	NEG	NEG	NEG	NEG	NEG	NEG	POS
Beaver H_Austria_B1	POS	POS	POS	POS	POS	POS	POS	NEG	NEG	NEG	NEG	NEG	NEG	NEG	NEG	NEG	NEG	NEG				POS
**>CC1956-MSSA**																						
Strain C6589: in silico predicted hybridisation pattern	POS	POS	POS	NEG	AMB	POS	NEG	NEG	NEG	NEG	NEG	NEG	NEG	NEG	NEG	POS	POS	POS	NEG	NEG	NEG	POS
Strain C6591: in silico predicted hybridisation pattern	POS	POS	POS	NEG	AMB	POS	NEG	NEG	NEG	NEG	NEG	NEG	NEG	NEG	NEG	POS	POS	POS	NEG	NEG	NEG	POS
Strain C6597: in silico predicted hybridisation pattern	POS	POS	POS	NEG	AMB	POS	NEG	NEG	NEG	NEG	NEG	NEG	NEG	NEG	NEG	POS	POS	POS	NEG	NEG	NEG	POS
Strain C6598: in silico predicted hybridisation pattern	POS	POS	POS	NEG	AMB	POS	NEG	NEG	NEG	NEG	NEG	NEG	NEG	NEG	NEG	POS	POS	POS	NEG	NEG	NEG	POS
Strain C6601: in silico predicted hybridisation pattern	POS	POS	POS	NEG	AMB	POS	NEG	NEG	NEG	NEG	NEG	NEG	NEG	NEG	NEG	POS	POS	POS	NEG	NEG	NEG	POS
**>CC1956-MSSA (lukF-PV+/lukS-PV?)**																						
Beaver A_Berlin_WT19: in silico predicted hybridisation pattern	POS	POS	POS	NEG	AMB	POS	NEG	NEG	NEG	NEG	NEG	NEG	NEG	NEG	NEG	POS	POS	POS	NEG	NEG	NEG	POS
Beaver A_Berlin_WT19	POS	POS	POS	NEG	POS	POS	NEG	NEG	NEG	NEG	NEG	NEG	NEG	NEG	NEG	POS	POS	POS	NEG	NEG		POS
Beaver B_Berlin_WT63	POS	POS	POS	NEG	POS	POS	NEG	NEG	NEG	NEG	NEG	NEG	NEG	NEG	NEG	POS	POS	POS	NEG	NEG		POS
Beaver B_Berlin_WT64	POS	POS	POS	NEG	POS	POS	NEG	NEG	NEG	NEG	NEG	NEG	NEG	NEG	NEG	POS	POS	POS				POS
Beaver D_Berlin_WT66	POS	POS	POS	NEG	POS	POS	NEG	NEG	NEG	NEG	NEG	NEG	NEG	NEG	NEG	POS	POS	POS				POS
Beaver D_Berlin_WT67A	POS	POS	POS	NEG	POS	POS	NEG	NEG	NEG	NEG	NEG	NEG	NEG	NEG	NEG	POS	POS	POS				POS
Beaver D_Berlin_WT67B	POS	POS	POS	NEG	POS	POS	NEG	NEG	NEG	NEG	NEG	NEG	NEG	NEG	NEG	POS	POS	POS	NEG	NEG		POS
Beaver D_Berlin_WT68	POS	POS	POS	NEG	NEG	POS	NEG	NEG	NEG	NEG	NEG	NEG	NEG	NEG	NEG	POS	NEG	POS	NEG	NEG		NEG
Beaver D_Berlin_WT69	POS	POS	POS	NEG	NEG	POS	NEG	NEG	NEG	NEG	NEG	NEG	NEG	NEG	NEG	POS	NEG	POS				NEG
Beaver F_Berlin_WT71	POS	POS	POS	NEG	POS	POS	NEG	NEG	NEG	NEG	NEG	NEG	NEG	NEG	NEG	POS	POS	POS				POS
Beaver E_Berlin_WT70	POS	POS	POS	NEG	POS	POS	NEG	NEG	NEG	NEG	NEG	NEG	NEG	NEG	NEG	POS	POS	POS				POS
Beaver G_Berlin_WT110	POS	POS	POS	NEG	POS	POS	NEG	NEG	NEG	NEG	NEG	NEG	NEG	NEG	NEG	POS	POS	POS				POS
Beaver G_Berlin_WT111	POS	POS	POS	NEG	POS	POS	NEG	NEG	NEG	NEG	NEG	NEG	NEG	NEG	NEG	POS	POS	POS				POS

STRAIN / ISOLATE	METHICILLIN RESISTANCE AND SCCmec TYPING																					
	ugpQ	mecA	delta_mecR1	mecR1	mecI	fudoh-PSM	cstB-SCC1 (ex Q2G1R6) (SCCmec II/III)	xylR/mecR2	mecC	mecR-SCCmec XI	mecR-S04009	mecI (SCCmec XI)	mecI (S04009)	blaZ (SCCmec XI) combined	mecA-vitulinus	mecR2-vitul (-aureus)	mecR2-lent	mecA-sciuri	mecR2-sciur	plsSCC (COL)	mvaS-SCC	Q5HJW6
	Glycerophosphoryl diester phosphodiesterase. Accompanies mecA in nearly all SCCmec sequences.	Modified penicillin binding protein (PBP2a)	Truncated methicillin resistance operon repressor 1. Truncated mecR1 is present in SCCmec I, IV, V, VI, VII; complete absence of mecR1 from SCCmec V, IX, X	Methicillin resistance operon repressor 1. Un-truncated sequence in SCCmec II, SCCmec III, SCCmec VIII	Methicillin-resistance regulatory protein. Present in SCCmec II (although absent from Irish SCCmec II variants C and E), SCCmec III, SCCmec VIII	Phenol soluble modulin from SCCmec	CsoR-like sulfur transferase-regulated genes B/metallo-beta-lactamase superfamily protein. Pseudogene containing two stop codons. Subtyping SCCmec II. Also present in SCCmec VIII and irregular	Methicillin resistance operon repressor 2, Homolog of xylose repressor. Located next to mec operon downstream of mecI (not present if mecI is truncated). Present in SCCmec II (although absent from Irish SCCmec II	Alternate gene encoding a modified penicillin binding protein . Present in, and characteristic for, SCCmec XI					Beta-lactamase from SCCmec XI						Plasmin-sensitive surface protein, prevents bacterial adhesion in vitro, located in SCC, close to mec operon. Subtyping SCCmec I	Truncated 3-hydroxy-3-methylglutaryl CoA synthase. Subtyping SCCmec I, II, IV, V	Putative protein next to dru
**>CC8-MSSA**																						
Strain NCTC8325: in silico predicted hybridisation pattern	NEG	NEG	NEG	NEG	NEG	NEG	NEG	NEG	NEG	NEG	NEG	NEG	NEG	NEG	NEG	NEG	NEG	NEG	NEG	NEG	NEG	NEG
Strain RN4220:VC40: in silico predicted hybridisation pattern	NEG	NEG	NEG	NEG	NEG	NEG	NEG	NEG	NEG	NEG	NEG	NEG	NEG	NEG	NEG	NEG	NEG	NEG	NEG	NEG	NEG	NEG
Strain Newman: in silico predicted hybridisation pattern	NEG	NEG	NEG	NEG	NEG	NEG	NEG	NEG	NEG	NEG	NEG	NEG	NEG	NEG	NEG	NEG	NEG	NEG	NEG	NEG	NEG	NEG
Beaver I_Austria_B2	NEG	NEG	NEG	NEG	NEG			NEG	NEG					NEG						NEG		
Beaver J_Austria_B3	NEG	NEG	NEG	NEG	NEG			NEG	NEG					NEG						NEG		
**>CC12-MSSA**																						
Strain KLT6: in silico predicted hybridisation pattern	NEG	NEG	NEG	NEG	NEG	NEG	NEG	NEG	NEG	NEG	NEG	NEG	NEG	NEG	NEG	NEG	NEG	NEG	NEG	NEG	NEG	NEG
Strain 21266: in silico predicted hybridisation pattern	NEG	NEG	NEG	NEG	NEG	NEG	NEG	NEG	NEG	NEG	NEG	NEG	NEG	NEG	NEG	NEG	NEG	NEG	NEG	NEG	NEG	NEG
NP66 chromosome: in silico predicted hybridisation pattern	NEG	NEG	NEG	NEG	NEG	NEG	NEG	NEG	NEG	NEG	NEG	NEG	NEG	NEG	NEG	NEG	NEG	NEG	NEG	NEG	NEG	NEG
Beaver K_Austria_B4	NEG	NEG	NEG	NEG	NEG			NEG	NEG					NEG						NEG		
**>CC49-MSSA**																						
Strain 21262: in silico predicted hybridisation pattern	NEG	NEG	NEG	NEG	NEG	NEG	NEG	NEG	NEG	NEG	NEG	NEG	NEG	NEG	NEG	NEG	NEG	NEG	NEG	NEG	NEG	NEG
Strain Tager 104: in silico predicted hybridisation pattern	NEG	NEG	NEG	NEG	NEG	NEG	NEG	NEG	NEG	NEG	NEG	NEG	NEG	NEG	NEG	NEG	NEG	NEG	NEG	NEG	NEG	NEG
**>CC49-MSSA (lukF-P83/lukM+)**																						
Strain 22_M61_07_10: in silico predicted hybridisation pattern	NEG	NEG	NEG	NEG	NEG	NEG	NEG	NEG	NEG	NEG	NEG	NEG	NEG	NEG	NEG	NEG	NEG	NEG	NEG	NEG	NEG	NEG
Strain 22_M48_10_10: in silico predicted hybridisation pattern	NEG	NEG	NEG	NEG	NEG	NEG	NEG	NEG	NEG	NEG	NEG	NEG	NEG	NEG	NEG	NEG	NEG	NEG	NEG	NEG	NEG	NEG
Strain 22_M47_10_10: in silico predicted hybridisation pattern	NEG	NEG	NEG	NEG	NEG	NEG	NEG	NEG	NEG	NEG	NEG	NEG	NEG	NEG	NEG	NEG	NEG	NEG	NEG	NEG	NEG	NEG
**>CC49-MSSA (lukF-PV+/lukS-PV?)**																						
Beaver C_Bavaria_WT65: in silico predicted hybridisation pattern	NEG	NEG	NEG	NEG	NEG	NEG	NEG	NEG	NEG	NEG	NEG	NEG	NEG	NEG	NEG	NEG	NEG	NEG	NEG	NEG	NEG	NEG
Beaver C_Bavaria_WT65	NEG	NEG	NEG	NEG	NEG	NEG	NEG	NEG	NEG	NEG	NEG	NEG	NEG	NEG	NEG	NEG	NEG	NEG	NEG	NEG	NEG	NEG
**>CC398-MSSA**																						
Strain 21331: in silico predicted hybridisation pattern	NEG	NEG	NEG	NEG	NEG	NEG	NEG	NEG	NEG	NEG	NEG	NEG	NEG	NEG	NEG	NEG	NEG	NEG	NEG	NEG	NEG	NEG
Strain 71193: in silico predicted hybridisation pattern	NEG	NEG	NEG	NEG	NEG	NEG	NEG	NEG	NEG	NEG	NEG	NEG	NEG	NEG	NEG	NEG	NEG	NEG	NEG	NEG	NEG	NEG
Strain S123: in silico predicted hybridisation pattern	NEG	NEG	NEG	NEG	NEG	NEG	NEG	NEG	NEG	NEG	NEG	NEG	NEG	NEG	NEG	NEG	NEG	NEG	NEG	NEG	NEG	NEG
Beaver H_Austria_B1	NEG	NEG	NEG	NEG	NEG			NEG	NEG					NEG						NEG		
**>CC1956-MSSA**																						
Strain C6589: in silico predicted hybridisation pattern	NEG	NEG	NEG	NEG	NEG	NEG	NEG	NEG	NEG	NEG	NEG	NEG	NEG	NEG	NEG	NEG	NEG	NEG	NEG	NEG	NEG	NEG
Strain C6591: in silico predicted hybridisation pattern	NEG	NEG	NEG	NEG	NEG	NEG	NEG	NEG	NEG	NEG	NEG	NEG	NEG	NEG	NEG	NEG	NEG	NEG	NEG	NEG	NEG	NEG
Strain C6597: in silico predicted hybridisation pattern	NEG	NEG	NEG	NEG	NEG	NEG	NEG	NEG	NEG	NEG	NEG	NEG	NEG	NEG	NEG	NEG	NEG	NEG	NEG	NEG	NEG	NEG
Strain C6598: in silico predicted hybridisation pattern	NEG	NEG	NEG	NEG	NEG	NEG	NEG	NEG	NEG	NEG	NEG	NEG	NEG	NEG	NEG	NEG	NEG	NEG	NEG	NEG	NEG	NEG
Strain C6601: in silico predicted hybridisation pattern	NEG	NEG	NEG	NEG	NEG	NEG	NEG	NEG	NEG	NEG	NEG	NEG	NEG	NEG	NEG	NEG	NEG	NEG	NEG	NEG	NEG	NEG
**>CC1956-MSSA (lukF-PV+/lukS-PV?)**																						
Beaver A_Berlin_WT19: in silico predicted hybridisation pattern	NEG	NEG	NEG	NEG	NEG	NEG	NEG	NEG	NEG	NEG	NEG	NEG	NEG	NEG	NEG	NEG	NEG	NEG	NEG	NEG	NEG	NEG
Beaver A_Berlin_WT19	NEG	NEG	NEG	NEG	NEG	NEG	NEG	NEG	NEG	NEG	NEG	NEG	NEG	NEG	NEG	NEG	NEG	NEG	NEG	NEG	NEG	NEG
Beaver B_Berlin_WT63	NEG	NEG	NEG	NEG	NEG	NEG	NEG	NEG	NEG	NEG	NEG	NEG	NEG	NEG	NEG	NEG	NEG	NEG	NEG	NEG	NEG	NEG
Beaver B_Berlin_WT64	NEG	NEG	NEG	NEG	NEG			NEG	NEG					NEG						NEG		
Beaver D_Berlin_WT66	NEG	NEG	NEG	NEG	NEG			NEG	NEG					NEG						NEG		
Beaver D_Berlin_WT67A	NEG	NEG	NEG	NEG	NEG			NEG	NEG					NEG						NEG		
Beaver D_Berlin_WT67B	NEG	NEG	NEG	NEG	NEG	NEG	NEG	NEG	NEG	NEG	NEG	NEG	NEG	NEG	NEG	NEG	NEG	NEG	NEG	NEG	NEG	NEG
Beaver D_Berlin_WT68	NEG	NEG	NEG	NEG	NEG	NEG	NEG	NEG	NEG	NEG	NEG	NEG	NEG	NEG	NEG	NEG	NEG	NEG	NEG	NEG	NEG	NEG
Beaver D_Berlin_WT69	NEG	NEG	NEG	NEG	NEG			NEG	NEG					NEG						NEG		
Beaver F_Berlin_WT71	NEG	NEG	NEG	NEG	NEG			NEG	NEG					NEG						NEG		
Beaver E_Berlin_WT70	NEG	NEG	NEG	NEG	NEG			NEG	NEG					NEG						NEG		
Beaver G_Berlin_WT110	NEG	NEG	NEG	NEG	NEG			NEG	NEG					NEG						NEG		
Beaver G_Berlin_WT111	NEG	NEG	NEG	NEG	NEG			NEG	NEG					NEG						NEG		

STRAIN / ISOLATE	METHICILLIN RESISTANCE AND SCCmec TYPING																					
	Q7A207	cstB-SCC2 (Q2G1R6)	Q9S0M4	kdpA-SCC	kdpB-SCC	kdpC-SCC	kdpD-SCC	kdpE-SCC	Q93IB7	D1GU38		Q933A2	D1GU55	F9JXC0		B2Y834	B6VQU0	Q3YK51	ydhK			C1PH94
										D1GU38	D1GU38 (TW20)			F9JXC0 (full)	F9JXC0 (trunc)				ydhK (FPR3757)	hp1473_ydhK)	hp1472_ydhK)	
	Putative protein	CsoR-like sulfur transferase-regulated genes B/metallo-beta-lactamase superfamily protein. Present in SCCmec IVa (truncated) and SCCmec X, variably present in SCCmec I (usually present, but absent from MR1	Putative protein. Subtyping SCCmec I, SCCmec/ACME composites and SCCmec from WA40	Potassium-translocating ATPase A, chain 2	Potassium-transporting ATPase B, chain 1	Potassium-translocating ATPase C, chain 2	Sensor kinase protein	KDP operon transcriptional regulatory protein	LytTR domain DNA-binding regulator. Subtyping SCCmec III	Subtyping SCCmec III, identification of SCCmec VT, SCCmec ZH47, SCCmec VII because of an association with (additional/second) ccrC copies	Putative protein	Putative ADP-ribosyltransferase. Subtyping SCCmec III and SCCmec IX	Putative membrane protein. Subtyping SCCmec III, additional marker for SCCmec VII	KDP operon transcriptional regulatory protein		Abortive phage resistance protein. Subtyping SCCmec IV, i.e., identification of SCCmec IV A, G, c and SCCmec MRSAZH47	Putative protein. Subtyping SCCmec IV, i.e., identification of SCCmec IVh/j	Putative protein. Subtyping SCCmec IV, i.e., identification of SCCmec IV g	Putative lipoprotein. Present in some composite elements comprising SCCmec and heavy metal resistance genes including the one in FPR3757, GenBank: CP000255.1			Putative protein
**>CC8-MSSA**																						
Strain NCTC8325: in silico predicted hybridisation pattern	NEG	NEG	NEG	NEG	NEG	NEG	NEG	NEG	NEG	NEG	NEG	NEG	NEG	NEG	NEG	NEG	NEG	NEG	NEG	NEG	NEG	NEG
Strain RN4220:VC40: in silico predicted hybridisation pattern	NEG	NEG	NEG	NEG	NEG	NEG	NEG	NEG	NEG	NEG	NEG	NEG	NEG	NEG	NEG	NEG	NEG	NEG	NEG	NEG	NEG	NEG
Strain Newman: in silico predicted hybridisation pattern	NEG	NEG	NEG	NEG	NEG	NEG	NEG	NEG	NEG	NEG	NEG	NEG	NEG	NEG	NEG	NEG	NEG	NEG	NEG	NEG	NEG	NEG
Beaver I_Austria_B2				NEG	NEG	NEG	NEG	NEG														
Beaver J_Austria_B3				NEG	NEG	NEG	NEG	NEG														
**>CC12-MSSA**																						
Strain KLT6: in silico predicted hybridisation pattern	NEG	NEG	NEG	NEG	NEG	NEG	NEG	NEG	NEG	NEG	NEG	NEG	NEG	NEG	NEG	NEG	NEG	NEG	NEG	NEG	NEG	NEG
Strain 21266: in silico predicted hybridisation pattern	NEG	NEG	NEG	NEG	NEG	NEG	NEG	NEG	NEG	NEG	NEG	NEG	NEG	NEG	NEG	NEG	NEG	NEG	NEG	NEG	NEG	NEG
NP66 chromosome: in silico predicted hybridisation pattern	NEG	NEG	NEG	NEG	NEG	NEG	NEG	NEG	NEG	NEG	NEG	NEG	NEG	NEG	NEG	NEG	NEG	NEG	NEG	NEG	NEG	NEG
Beaver K_Austria_B4				NEG	NEG	NEG	NEG	NEG														
**>CC49-MSSA**																						
Strain 21262: in silico predicted hybridisation pattern	NEG	NEG	NEG	NEG	NEG	NEG	NEG	NEG	NEG	NEG	NEG	NEG	NEG	NEG	NEG	NEG	NEG	NEG	NEG	NEG	NEG	POS
Strain Tager 104: in silico predicted hybridisation pattern	NEG	NEG	NEG	NEG	NEG	NEG	NEG	NEG	NEG	NEG	NEG	NEG	NEG	NEG	NEG	NEG	NEG	NEG	NEG	NEG	NEG	POS
**>CC49-MSSA (lukF-P83/lukM+)**																						
Strain 22_M61_07_10: in silico predicted hybridisation pattern	NEG	NEG	NEG	NEG	NEG	NEG	NEG	NEG	NEG	NEG	NEG	NEG	NEG	NEG	NEG	NEG	NEG	NEG	NEG	NEG	NEG	POS
Strain 22_M48_10_10: in silico predicted hybridisation pattern	NEG	NEG	NEG	NEG	NEG	NEG	NEG	NEG	NEG	NEG	NEG	NEG	NEG	NEG	NEG	NEG	NEG	NEG	NEG	NEG	NEG	POS
Strain 22_M47_10_10: in silico predicted hybridisation pattern	NEG	NEG	NEG	NEG	NEG	NEG	NEG	NEG	NEG	NEG	NEG	NEG	NEG	NEG	NEG	NEG	NEG	NEG	NEG	NEG	NEG	POS
**>CC49-MSSA (lukF-PV+/lukS-PV?)**																						
Beaver C_Bavaria_WT65: in silico predicted hybridisation pattern	NEG	NEG	NEG	NEG	NEG	NEG	NEG	NEG	NEG	NEG	NEG	NEG	NEG	NEG	NEG	NEG	NEG	NEG	NEG	NEG	NEG	POS
Beaver C_Bavaria_WT65	NEG	NEG	NEG	NEG	NEG	NEG	NEG	NEG	NEG	NEG	NEG	NEG	NEG	NEG	NEG	NEG	NEG	NEG	NEG	NEG	NEG	POS
**>CC398-MSSA**																						
Strain 21331: in silico predicted hybridisation pattern	NEG	NEG	NEG	NEG	NEG	NEG	NEG	NEG	NEG	NEG	NEG	NEG	NEG	NEG	NEG	NEG	NEG	NEG	NEG	NEG	NEG	NEG
Strain 71193: in silico predicted hybridisation pattern	NEG	NEG	NEG	NEG	NEG	NEG	NEG	NEG	NEG	NEG	NEG	NEG	NEG	NEG	NEG	NEG	NEG	NEG	NEG	NEG	NEG	NEG
Strain S123: in silico predicted hybridisation pattern	NEG	NEG	NEG	NEG	NEG	NEG	NEG	NEG	NEG	NEG	NEG	NEG	NEG	NEG	NEG	NEG	NEG	NEG	NEG	POS	POS	NEG
Beaver H_Austria_B1				NEG	NEG	NEG	NEG	NEG														
**>CC1956-MSSA**																						
Strain C6589: in silico predicted hybridisation pattern	NEG	NEG	NEG	NEG	NEG	NEG	NEG	NEG	NEG	NEG	NEG	NEG	NEG	NEG	NEG	NEG	NEG	NEG	NEG	NEG	NEG	NEG
Strain C6591: in silico predicted hybridisation pattern	NEG	NEG	NEG	NEG	NEG	NEG	NEG	NEG	NEG	NEG	NEG	NEG	NEG	NEG	NEG	NEG	NEG	NEG	NEG	NEG	NEG	NEG
Strain C6597: in silico predicted hybridisation pattern	NEG	NEG	NEG	NEG	NEG	NEG	NEG	NEG	NEG	NEG	NEG	NEG	NEG	NEG	NEG	NEG	NEG	NEG	NEG	NEG	NEG	NEG
Strain C6598: in silico predicted hybridisation pattern	NEG	NEG	NEG	NEG	NEG	NEG	NEG	NEG	NEG	NEG	NEG	NEG	NEG	NEG	NEG	NEG	NEG	NEG	NEG	NEG	NEG	NEG
Strain C6601: in silico predicted hybridisation pattern	NEG	NEG	NEG	NEG	NEG	NEG	NEG	NEG	NEG	NEG	NEG	NEG	NEG	NEG	NEG	NEG	NEG	NEG	NEG	NEG	NEG	NEG
**>CC1956-MSSA (lukF-PV+/lukS-PV?)**																						
Beaver A_Berlin_WT19: in silico predicted hybridisation pattern	NEG	NEG	NEG	NEG	NEG	NEG	NEG	NEG	NEG	NEG	NEG	NEG	NEG	NEG	NEG	NEG	NEG	NEG	NEG	NEG	NEG	NEG
Beaver A_Berlin_WT19	NEG	NEG	NEG	NEG	NEG	NEG	NEG	NEG	NEG	NEG	NEG	NEG	NEG	NEG	NEG	NEG	NEG	NEG	NEG	NEG	NEG	NEG
Beaver B_Berlin_WT63	NEG	NEG	NEG	NEG	NEG	NEG	NEG	NEG	NEG	NEG	NEG	NEG	NEG	NEG	NEG	NEG	NEG	NEG	NEG	NEG	NEG	NEG
Beaver B_Berlin_WT64				NEG	NEG	NEG	NEG	NEG														
Beaver D_Berlin_WT66				NEG	NEG	NEG	NEG	NEG														
Beaver D_Berlin_WT67A				NEG	NEG	NEG	NEG	NEG														
Beaver D_Berlin_WT67B	NEG	NEG	NEG	NEG	NEG	NEG	NEG	NEG	NEG	NEG	NEG	NEG	NEG	NEG	NEG	NEG	NEG	NEG	NEG	NEG	NEG	NEG
Beaver D_Berlin_WT68	NEG	NEG	NEG	NEG	NEG	NEG	NEG	NEG	NEG	NEG	NEG	NEG	NEG	NEG	NEG	NEG	NEG	NEG	NEG	NEG	NEG	NEG
Beaver D_Berlin_WT69				NEG	NEG	NEG	NEG	NEG														
Beaver F_Berlin_WT71				NEG	NEG	NEG	NEG	NEG														
Beaver E_Berlin_WT70				NEG	NEG	NEG	NEG	NEG														
Beaver G_Berlin_WT110				NEG	NEG	NEG	NEG	NEG														
Beaver G_Berlin_WT111				NEG	NEG	NEG	NEG	NEG														

STRAIN / ISOLATE	METHICILLIN RESISTANCE AND SCCmec TYPING																		
							cas1												
	DUF1958	Q4LAG7 (SCCmecV, SO385)	Q4LAG7 (SCCfus 45394F/MSSA 476)	C5QAP8 (SCCmec XI)	Q8CU82	D3JD07	cas1 (MSHR1132)	cas1 (M06-171)	fusC (Q6GD50)	tirS	yeaA	arcA-SCC	arcB-SCC	arcC-SCC	arcD-SCC	opp3B	opp3C	adhC	speG
	Putative protein. Subtyping SCCmec VT. Present, e.g., in PM1, GenBank BAFA but absent, e.g., in Strain 3957, GenBank AOFU	Putative protein located within SCCmec type V elements	Putative protein located within SCCfus elements	Putative protein. Identification of SCCmec XI	Putative protein. Present in some SCCmec/fus composite elements.	Putative protein. Present in some composite SCC elements	CRISPR-associated endonuclease 1. Present in MSHR1132	CRISPR-associated endonuclease 1. Present in M06/0171, GenBank HE980450.1	SCC-associated fusidic acid resistance gene. Present in "SCCfus" elements or together with mecA in composite elements.	Staphylococcal TIR-protein binding protein. Subtyping SCCfus because it is frequently, but not always, accompanying fusC	Putative DNA methyltransferase. Subtyping SCCmecIV/fus composite elements	Arginine deiminase	Ornithine carbamoyltransferase	Carbamate kinase	Arginine/ornithine antiporter	Oligopeptide permease, channel-forming protein	Oligopeptide permease, channel-forming protein	Alcohol dehydrogenase, zinc-containing	Spermidine N-acetyltransferase. Usually associated with ACME or composite SCCmec/ACME elements
**>CC8-MSSA**																			
Strain NCTC8325: in silico predicted hybridisation pattern	NEG	NEG	NEG	NEG	NEG	NEG	NEG	NEG	NEG	NEG	NEG	NEG	NEG	NEG	NEG	NEG	NEG	NEG	NEG
Strain RN4220:VC40: in silico predicted hybridisation pattern	NEG	NEG	NEG	NEG	NEG	NEG	NEG	NEG	NEG	NEG	NEG	NEG	NEG	NEG	NEG	NEG	NEG	NEG	NEG
Strain Newman: in silico predicted hybridisation pattern	NEG	NEG	NEG	NEG	NEG	NEG	NEG	NEG	NEG	NEG	NEG	NEG	NEG	NEG	NEG	NEG	NEG	NEG	NEG
Beaver I_Austria_B2									NEG			NEG	NEG	NEG	NEG				
Beaver J_Austria_B3									NEG			NEG	NEG	NEG	NEG				
**>CC12-MSSA**																			
Strain KLT6: in silico predicted hybridisation pattern	NEG	NEG	NEG	NEG	NEG	NEG	NEG	NEG	NEG	NEG	NEG	NEG	NEG	NEG	NEG	NEG	NEG	NEG	NEG
Strain 21266: in silico predicted hybridisation pattern	NEG	NEG	NEG	NEG	NEG	NEG	NEG	NEG	NEG	NEG	NEG	NEG	NEG	NEG	NEG	NEG	NEG	NEG	NEG
NP66 chromosome: in silico predicted hybridisation pattern	NEG	NEG	NEG	NEG	NEG	NEG	NEG	NEG	NEG	NEG	NEG	NEG	NEG	NEG	NEG	NEG	NEG	NEG	NEG
Beaver K_Austria_B4									NEG			NEG	NEG	NEG	NEG				
**>CC49-MSSA**																			
Strain 21262: in silico predicted hybridisation pattern	NEG	NEG	NEG	NEG	NEG	NEG	NEG	NEG	NEG	NEG	NEG	NEG	NEG	NEG	NEG	NEG	NEG	NEG	NEG
Strain Tager 104: in silico predicted hybridisation pattern	NEG	NEG	NEG	NEG	NEG	NEG	NEG	NEG	NEG	NEG	NEG	NEG	NEG	NEG	NEG	NEG	NEG	NEG	NEG
**>CC49-MSSA (lukF-P83/lukM+)**																			
Strain 22_M61_07_10: in silico predicted hybridisation pattern	NEG	NEG	NEG	NEG	NEG	NEG	NEG	NEG	NEG	NEG	NEG	NEG	NEG	NEG	NEG	NEG	NEG	NEG	NEG
Strain 22_M48_10_10: in silico predicted hybridisation pattern	NEG	NEG	NEG	NEG	NEG	NEG	NEG	NEG	NEG	NEG	NEG	NEG	NEG	NEG	NEG	NEG	NEG	NEG	NEG
Strain 22_M47_10_10: in silico predicted hybridisation pattern	NEG	NEG	NEG	NEG	NEG	NEG	NEG	NEG	NEG	NEG	NEG	NEG	NEG	NEG	NEG	NEG	NEG	NEG	NEG
**>CC49-MSSA (lukF-PV+/lukS-PV?)**																			
Beaver C_Bavaria_WT65: in silico predicted hybridisation pattern	NEG	NEG	NEG	NEG	NEG	NEG	NEG	NEG	NEG	NEG	NEG	NEG	NEG	NEG	NEG	NEG	NEG	NEG	NEG
Beaver C_Bavaria_WT65	NEG	NEG	NEG	NEG	NEG	NEG	NEG	NEG	NEG	NEG	NEG	NEG	NEG	NEG	NEG	NEG	NEG	NEG	NEG
**>CC398-MSSA**																			
Strain 21331: in silico predicted hybridisation pattern	NEG	NEG	NEG	NEG	NEG	NEG	NEG	NEG	NEG	NEG	NEG	NEG	NEG	NEG	NEG	NEG	NEG	NEG	NEG
Strain 71193: in silico predicted hybridisation pattern	NEG	NEG	NEG	NEG	NEG	NEG	NEG	NEG	NEG	NEG	NEG	NEG	NEG	NEG	NEG	NEG	NEG	NEG	NEG
Strain S123: in silico predicted hybridisation pattern	NEG	NEG	NEG	NEG	NEG	NEG	NEG	NEG	NEG	NEG	NEG	NEG	NEG	NEG	NEG	NEG	NEG	NEG	NEG
Beaver H_Austria_B1									NEG			NEG	NEG	NEG	NEG				
**>CC1956-MSSA**																			
Strain C6589: in silico predicted hybridisation pattern	NEG	NEG	NEG	NEG	NEG	NEG	NEG	NEG	NEG	NEG	NEG	NEG	NEG	NEG	NEG	NEG	NEG	NEG	NEG
Strain C6591: in silico predicted hybridisation pattern	NEG	NEG	NEG	NEG	NEG	NEG	NEG	NEG	NEG	NEG	NEG	NEG	NEG	NEG	NEG	NEG	NEG	NEG	NEG
Strain C6597: in silico predicted hybridisation pattern	NEG	NEG	NEG	NEG	NEG	NEG	NEG	NEG	NEG	NEG	NEG	NEG	NEG	NEG	NEG	NEG	NEG	NEG	NEG
Strain C6598: in silico predicted hybridisation pattern	NEG	NEG	NEG	NEG	NEG	NEG	NEG	NEG	NEG	NEG	NEG	NEG	NEG	NEG	NEG	NEG	NEG	NEG	NEG
Strain C6601: in silico predicted hybridisation pattern	NEG	NEG	NEG	NEG	NEG	NEG	NEG	NEG	NEG	NEG	NEG	NEG	NEG	NEG	NEG	NEG	NEG	NEG	NEG
**>CC1956-MSSA (lukF-PV+/lukS-PV?)**																			
Beaver A_Berlin_WT19: in silico predicted hybridisation pattern	NEG	NEG	NEG	NEG	NEG	NEG	NEG	NEG	NEG	NEG	NEG	NEG	NEG	NEG	NEG	NEG	NEG	NEG	NEG
Beaver A_Berlin_WT19	NEG	NEG	NEG	NEG	NEG	NEG	NEG	NEG	NEG	NEG	NEG	NEG	NEG	NEG	NEG	NEG	NEG	NEG	NEG
Beaver B_Berlin_WT63	NEG	NEG	NEG	NEG	NEG	NEG	NEG	NEG	NEG	NEG	NEG	NEG	NEG	NEG	NEG	NEG	NEG	NEG	NEG
Beaver B_Berlin_WT64									NEG			NEG	NEG	NEG	NEG				
Beaver D_Berlin_WT66									NEG			NEG	NEG	NEG	NEG				
Beaver D_Berlin_WT67A									NEG			NEG	NEG	NEG	NEG				
Beaver D_Berlin_WT67B	NEG	NEG	NEG	NEG	NEG	NEG	NEG	NEG	NEG	NEG	NEG	NEG	NEG	NEG	NEG	NEG	NEG	NEG	NEG
Beaver D_Berlin_WT68	NEG	NEG	NEG	NEG	NEG	NEG	NEG	NEG	NEG	NEG	NEG	NEG	NEG	NEG	NEG	NEG	NEG	NEG	NEG
Beaver D_Berlin_WT69									NEG			NEG	NEG	NEG	NEG				
Beaver F_Berlin_WT71									NEG			NEG	NEG	NEG	NEG				
Beaver E_Berlin_WT70									NEG			NEG	NEG	NEG	NEG				
Beaver G_Berlin_WT110									NEG			NEG	NEG	NEG	NEG				
Beaver G_Berlin_WT111									NEG			NEG	NEG	NEG	NEG				

STRAIN / ISOLATE	METHICILLIN RESISTANCE AND SCCmec TYPING																					
	ccrA/B-01		ccrA/B-02		ccrA/B-03		ccrAA/C		ccrA/B-04			Alternative SCC termini										
	ccrA-1	ccrB-1	ccrA-2	ccrB-2	ccrA-3	ccrB-3	ccrAA	ccrC (85-2082)	ccrA-4	ccrB-4	Q9XB68-dcs	SCCmec Terminus 1	SCCmec Terminus 2	SCCmec Terminus 3	SCCmec Terminus 4	SCCmec Terminus 5	SCCmec Terminus 6	SCCmec Terminus 7	SCCmec Terminus 9	SCCmec Terminus 10	SCCmec Terminus 11	SCCmec Terminus 12/13/14
	Cassette chromosome recombinase A, type 1	Cassette chromosome recombinase B, type 1	Cassette chromosome recombinase A, type 2	Cassette chromosome recombinase B, type 2	Cassette chromosome recombinase A, type 3	Cassette chromosome recombinase B, type 3	hypoth. Protein associated with ccrC	Cassette chromosome recombinase C	Cassette chromosome recombinase A, type 4	Cassette chromosome recombinase B, type 4	Located at the terminus of SCCmec next to orfX.This locus comprises the downstream constant segment (dcs) that in turn comprises a copy of the SCC direct repeat	SCC integration site alternate to dcs										
>CC8-MSSA																						
Strain NCTC8325: in silico predicted hybridisation pattern	NEG	NEG	NEG	NEG	NEG	NEG	NEG	NEG	NEG	NEG	NEG	NEG	NEG	NEG	NEG	NEG	NEG	NEG	NEG	NEG	NEG	NEG
Strain RN4220:VC40: in silico predicted hybridisation pattern	NEG	NEG	NEG	NEG	NEG	NEG	NEG	NEG	NEG	NEG	NEG	NEG	NEG	NEG	NEG	NEG	NEG	NEG	NEG	NEG	NEG	NEG
Strain Newman: in silico predicted hybridisation pattern	NEG	NEG	NEG	NEG	NEG	NEG	NEG	NEG	NEG	NEG	NEG	NEG	NEG	NEG	NEG	NEG	NEG	NEG	NEG	NEG	NEG	NEG
Beaver I_Austria_B2	NEG	NEG	NEG	NEG	NEG	NEG	NEG	NEG	NEG	NEG	NEG											
Beaver J_Austria_B3	NEG	NEG	NEG	NEG	NEG	NEG	NEG	NEG	NEG	NEG	NEG											
>CC12-MSSA																						
Strain KLT6: in silico predicted hybridisation pattern	NEG	NEG	NEG	NEG	NEG	NEG	NEG	NEG	NEG	NEG	NEG	NEG	NEG	NEG	NEG	NEG	NEG	NEG	NEG	NEG	NEG	NEG
Strain 21266: in silico predicted hybridisation pattern	NEG	NEG	NEG	NEG	NEG	NEG	NEG	NEG	NEG	NEG	NEG	NEG	NEG	NEG	NEG	NEG	NEG	NEG	NEG	NEG	NEG	NEG
NP66 chromosome: in silico predicted hybridisation pattern	NEG	NEG	NEG	NEG	NEG	NEG	NEG	NEG	NEG	NEG	NEG	NEG	NEG	NEG	NEG	NEG	NEG	NEG	NEG	NEG	NEG	NEG
Beaver K_Austria_B4	NEG	NEG	NEG	NEG	NEG	NEG	NEG	NEG	NEG	NEG	NEG											
>CC49-MSSA																						
Strain 21262: in silico predicted hybridisation pattern	NEG	NEG	NEG	NEG	NEG	NEG	NEG	NEG	NEG	NEG	NEG	NEG	NEG	NEG	NEG	NEG	NEG	NEG	NEG	NEG	NEG	NEG
Strain Tager 104: in silico predicted hybridisation pattern	NEG	NEG	NEG	NEG	NEG	NEG	NEG	NEG	NEG	NEG	NEG	NEG	NEG	NEG	NEG	NEG	NEG	NEG	NEG	NEG	NEG	NEG
>CC49-MSSA (lukF-P83/lukM+)																						
Strain 22_M61_07_10: in silico predicted hybridisation pattern	NEG	NEG	NEG	NEG	NEG	NEG	NEG	NEG	NEG	NEG	NEG	NEG	NEG	NEG	NEG	NEG	NEG	NEG	NEG	NEG	NEG	NEG
Strain 22_M48_10_10: in silico predicted hybridisation pattern	NEG	NEG	NEG	NEG	NEG	NEG	NEG	NEG	NEG	NEG	NEG	NEG	NEG	NEG	NEG	NEG	NEG	NEG	NEG	NEG	NEG	NEG
Strain 22_M47_10_10: in silico predicted hybridisation pattern	NEG	NEG	NEG	NEG	NEG	NEG	NEG	NEG	NEG	NEG	NEG	NEG	NEG	NEG	NEG	NEG	NEG	NEG	NEG	NEG	NEG	NEG
>CC49-MSSA (lukF-PV+/lukS-PV?)																						
Beaver C_Bavaria_WT65: in silico predicted hybridisation pattern	NEG	NEG	NEG	NEG	NEG	NEG	NEG	NEG	NEG	NEG	NEG	NEG	NEG	NEG	NEG	NEG	NEG	NEG	NEG	NEG	NEG	NEG
Beaver C_Bavaria_WT65	NEG	NEG	NEG	NEG	NEG	NEG	NEG	NEG	NEG	NEG	NEG	NEG	NEG	NEG	NEG	NEG	NEG	NEG	NEG	NEG	NEG	NEG
>CC398-MSSA																						
Strain 21331: in silico predicted hybridisation pattern	NEG	NEG	NEG	NEG	NEG	NEG	NEG	NEG	NEG	NEG	NEG	NEG	NEG	NEG	NEG	NEG	NEG	NEG	NEG	NEG	NEG	NEG
Strain 71193: in silico predicted hybridisation pattern	NEG	NEG	NEG	NEG	NEG	NEG	NEG	NEG	NEG	NEG	NEG	NEG	NEG	NEG	NEG	NEG	NEG	NEG	NEG	NEG	NEG	NEG
Strain S123: in silico predicted hybridisation pattern	NEG	NEG	NEG	NEG	NEG	NEG	NEG	NEG	NEG	NEG	NEG	NEG	NEG	NEG	NEG	NEG	NEG	NEG	NEG	NEG	NEG	NEG
Beaver H_Austria_B1	NEG	NEG	NEG	NEG	NEG	NEG	NEG	NEG	NEG	NEG	NEG											
>CC1956-MSSA																						
Strain C6589: in silico predicted hybridisation pattern	NEG	NEG	NEG	NEG	NEG	NEG	NEG	NEG	NEG	NEG	NEG	NEG	NEG	NEG	NEG	NEG	NEG	NEG	NEG	NEG	NEG	NEG
Strain C6591: in silico predicted hybridisation pattern	NEG	NEG	NEG	NEG	NEG	NEG	NEG	NEG	NEG	NEG	NEG	NEG	NEG	NEG	NEG	NEG	NEG	NEG	NEG	NEG	NEG	NEG
Strain C6597: in silico predicted hybridisation pattern	NEG	NEG	NEG	NEG	NEG	NEG	NEG	NEG	NEG	NEG	NEG	NEG	NEG	NEG	NEG	NEG	NEG	NEG	NEG	NEG	NEG	NEG
Strain C6598: in silico predicted hybridisation pattern	NEG	NEG	NEG	NEG	NEG	NEG	NEG	NEG	NEG	NEG	NEG	NEG	NEG	NEG	NEG	NEG	NEG	NEG	NEG	NEG	NEG	NEG
Strain C6601: in silico predicted hybridisation pattern	NEG	NEG	NEG	NEG	NEG	NEG	NEG	NEG	NEG	NEG	NEG	NEG	NEG	NEG	NEG	NEG	NEG	NEG	NEG	NEG	NEG	NEG
>CC1956-MSSA (lukF-PV+/lukS-PV?)																						
Beaver A_Berlin_WT19: in silico predicted hybridisation pattern	NEG	NEG	NEG	NEG	NEG	NEG	NEG	NEG	NEG	NEG	NEG	NEG	NEG	NEG	NEG	NEG	NEG	NEG	NEG	NEG	NEG	NEG
Beaver A_Berlin_WT19	NEG	NEG	NEG	NEG	NEG	NEG	NEG	NEG	NEG	NEG	NEG	NEG	NEG	NEG	NEG	NEG	NEG	NEG	NEG	NEG	NEG	NEG
Beaver B_Berlin_WT63	NEG	NEG	NEG	NEG	NEG	NEG	NEG	NEG	NEG	NEG	NEG	NEG	NEG	NEG	NEG	NEG	NEG	NEG	NEG	NEG	NEG	NEG
Beaver B_Berlin_WT64	NEG	NEG	NEG	NEG	NEG	NEG	NEG	NEG	NEG	NEG	NEG											
Beaver D_Berlin_WT66	NEG	NEG	NEG	NEG	NEG	NEG	NEG	NEG	NEG	NEG	NEG											
Beaver D_Berlin_WT67A	NEG	NEG	NEG	NEG	NEG	NEG	NEG	NEG	NEG	NEG	NEG											
Beaver D_Berlin_WT67B	NEG	NEG	NEG	NEG	NEG	NEG	NEG	NEG	NEG	NEG	NEG	NEG	NEG	NEG	NEG	NEG	NEG	NEG	NEG	NEG	NEG	NEG
Beaver D_Berlin_WT68	NEG	NEG	NEG	NEG	NEG	NEG	NEG	NEG	NEG	NEG	NEG	NEG	NEG	NEG	NEG	NEG	NEG	NEG	NEG	NEG	NEG	NEG
Beaver D_Berlin_WT69	NEG	NEG	NEG	NEG	NEG	NEG	NEG	NEG	NEG	NEG	NEG											
Beaver F_Berlin_WT71	NEG	NEG	NEG	NEG	NEG	NEG	NEG	NEG	NEG	NEG	NEG											
Beaver E_Berlin_WT70	NEG	NEG	NEG	NEG	NEG	NEG	NEG	NEG	NEG	NEG	NEG											
Beaver G_Berlin_WT110	NEG	NEG	NEG	NEG	NEG	NEG	NEG	NEG	NEG	NEG	NEG											
Beaver G_Berlin_WT111	NEG	NEG	NEG	NEG	NEG	NEG	NEG	NEG	NEG	NEG	NEG											

STRAIN / ISOLATE	HEAVY METAL RESISTANCES, SCCmec AND OTHERS																					
										arsB			arsC						cadD			
	merA	merB	mco -plasmid	copA2 - plasmid	copA2 -SCC	mco -SCC	arsA	arsD	arsD2	arsB (SCC)	arsB (chromosomal)	arsB (plasmidic)	arsC (chromosomal)	arsC (SCC or plasmidic)	czrB	czrC	cadA	cadC	cadD (total)	cadD (R35)	cadX (plasmid)	cadX (SCC)
	mercury resistance operon		Multi copper oxidase	Copper exporting ATPase	Copper exporting ATPase	Multi copper oxidase	arsenical pump-driving ATPase	Putative dehydrogenase	trans-acting repressor of arsenic resistance operon	arsenical pump membrane protein			arsenate reductase		zink and cobalt transporter protein	cadmium and zinc resistance gene C, heavy metal translocating P-type ATPase. Frequently associated with SCCmec elements from livestock MRSA	cadmium transporting ATPase	putative regulator of cadmium efflux	cadmium transport protein D		Putative regulator of cadmium efflux	
**>CC8-MSSA**																						
Strain NCTC8325: in silico predicted hybridisation pattern	NEG	NEG	NEG	NEG	NEG	NEG	NEG	NEG	NEG	NEG	POS	NEG	POS	NEG	POS	NEG	NEG	NEG	NEG	NEG	NEG	NEG
Strain RN4220:VC40: in silico predicted hybridisation pattern	NEG	NEG	NEG	NEG	NEG	NEG	NEG	NEG	NEG	NEG	POS	NEG	POS	NEG	POS	NEG	NEG	NEG	NEG	NEG	NEG	NEG
Strain Newman: in silico predicted hybridisation pattern	NEG	NEG	NEG	NEG	NEG	NEG	NEG	NEG	NEG	NEG	POS	NEG	POS	NEG	POS	NEG	NEG	NEG	NEG	NEG	NEG	NEG
Beaver I_Austria_B2	NEG	NEG																				
Beaver J_Austria_B3	NEG	NEG																				
**>CC12-MSSA**																						
Strain KLT6: in silico predicted hybridisation pattern	NEG	NEG	NEG	NEG	NEG	NEG	NEG	NEG	NEG	NEG	POS	NEG	NEG	NEG	POS	NEG	NEG	NEG	POS	NEG	POS	NEG
Strain 21266: in silico predicted hybridisation pattern	NEG	NEG	NEG	NEG	NEG	NEG	NEG	NEG	NEG	NEG	POS	NEG	NEG	NEG	POS	NEG	NEG	NEG	POS	NEG	POS	NEG
NP66 chromosome: in silico predicted hybridisation pattern	NEG	NEG	NEG	NEG	NEG	NEG	NEG	NEG	NEG	NEG	POS	NEG	NEG	NEG	POS	NEG	NEG	NEG	NEG	NEG	NEG	NEG
Beaver K_Austria_B4	NEG	NEG																				
**>CC49-MSSA**																						
Strain 21262: in silico predicted hybridisation pattern	NEG	NEG	POS	POS	NEG	NEG	NEG	NEG	NEG	NEG	POS	POS	POS	POS	POS	NEG	POS	POS	NEG	NEG	NEG	NEG
Strain Tager 104: in silico predicted hybridisation pattern	NEG	NEG	POS	POS	NEG	NEG	NEG	NEG	NEG	NEG	POS	POS	POS	POS	POS	NEG	POS	POS	NEG	NEG	NEG	NEG
**>CC49-MSSA (lukF-P83/lukM+)**																						
Strain 22_M61_07_10: in silico predicted hybridisation pattern	NEG	NEG	NEG	NEG	NEG	NEG	NEG	NEG	NEG	NEG	POS	NEG	POS	NEG	POS	NEG	NEG	NEG	NEG	NEG	NEG	NEG
Strain 22_M48_10_10: in silico predicted hybridisation pattern	NEG	NEG	NEG	NEG	NEG	NEG	NEG	NEG	NEG	NEG	POS	NEG	POS	NEG	POS	NEG	NEG	NEG	NEG	NEG	NEG	NEG
Strain 22_M47_10_10: in silico predicted hybridisation pattern	NEG	NEG	NEG	NEG	NEG	NEG	NEG	NEG	NEG	NEG	POS	NEG	POS	NEG	POS	NEG	NEG	NEG	NEG	NEG	NEG	NEG
**>CC49-MSSA (lukF-PV+/lukS-PV?)**																						
Beaver C_Bavaria_WT65: in silico predicted hybridisation pattern	NEG	NEG	NEG	NEG	NEG	NEG	NEG	NEG	NEG	NEG	POS	NEG	POS	NEG	POS	NEG	NEG	NEG	NEG	NEG	NEG	NEG
Beaver C_Bavaria_WT65	NEG	NEG	NEG	NEG	NEG	NEG	NEG	NEG	NEG	NEG	POS	NEG	POS	NEG	POS	NEG	NEG	NEG	NEG	NEG	NEG	NEG
**>CC398-MSSA**																						
Strain 21331: in silico predicted hybridisation pattern	NEG	NEG	NEG	NEG	NEG	NEG	NEG	NEG	NEG	NEG	POS	NEG	NEG	NEG	POS	NEG	NEG	NEG	AMB	NEG	POS	NEG
Strain 71193: in silico predicted hybridisation pattern	NEG	NEG	NEG	NEG	NEG	NEG	NEG	NEG	NEG	NEG	POS	NEG	NEG	NEG	POS	NEG	NEG	NEG	AMB	NEG	POS	NEG
Strain S123: in silico predicted hybridisation pattern	NEG	NEG	POS	POS	NEG	NEG	NEG	NEG	NEG	NEG	POS	NEG	NEG	NEG	POS	NEG	NEG	NEG	NEG	NEG	NEG	NEG
Beaver H_Austria_B1	NEG	NEG																				
**>CC1956-MSSA**																						
Strain C6589: in silico predicted hybridisation pattern	NEG	NEG	NEG	NEG	NEG	NEG	NEG	NEG	NEG	NEG	POS	NEG	NEG	NEG	POS	NEG	NEG	NEG	NEG	NEG	NEG	NEG
Strain C6591: in silico predicted hybridisation pattern	NEG	NEG	NEG	NEG	NEG	NEG	NEG	NEG	NEG	NEG	POS	NEG	NEG	NEG	POS	NEG	NEG	NEG	NEG	NEG	NEG	NEG
Strain C6597: in silico predicted hybridisation pattern	NEG	NEG	NEG	NEG	NEG	NEG	NEG	NEG	NEG	NEG	POS	NEG	NEG	NEG	POS	NEG	NEG	NEG	NEG	NEG	NEG	NEG
Strain C6598: in silico predicted hybridisation pattern	NEG	NEG	NEG	NEG	NEG	NEG	NEG	NEG	NEG	NEG	POS	NEG	NEG	NEG	POS	NEG	NEG	NEG	NEG	NEG	NEG	NEG
Strain C6601: in silico predicted hybridisation pattern	NEG	NEG	NEG	NEG	NEG	NEG	NEG	NEG	NEG	NEG	POS	NEG	NEG	NEG	POS	NEG	NEG	NEG	NEG	NEG	NEG	NEG
**>CC1956-MSSA (lukF-PV+/lukS-PV?)**																						
Beaver A_Berlin_WT19: in silico predicted hybridisation pattern	NEG	NEG	NEG	NEG	NEG	NEG	NEG	NEG	NEG	NEG	POS	NEG	NEG	NEG	POS	NEG	NEG	NEG	NEG	NEG	NEG	NEG
Beaver A_Berlin_WT19	NEG	NEG	NEG	NEG	NEG	NEG	NEG	NEG	NEG	NEG	POS	NEG	NEG	NEG	POS	NEG	NEG	NEG	NEG	NEG	NEG	NEG
Beaver B_Berlin_WT63	NEG	NEG	NEG	NEG	NEG	NEG	NEG	NEG	NEG	NEG	POS	NEG	NEG	NEG	POS	NEG	NEG	NEG	NEG	NEG	NEG	NEG
Beaver B_Berlin_WT64	NEG	NEG																				
Beaver D_Berlin_WT66	NEG	NEG																				
Beaver D_Berlin_WT67A	NEG	NEG																				
Beaver D_Berlin_WT67B	NEG	NEG	NEG	NEG	NEG	NEG	NEG	NEG	NEG	NEG	POS	NEG	NEG	NEG	POS	NEG	NEG	NEG	NEG	NEG	NEG	NEG
Beaver D_Berlin_WT68	NEG	NEG	NEG	NEG	NEG	NEG	NEG	NEG	NEG	NEG	POS	NEG	NEG	NEG	POS	NEG	NEG	NEG	NEG	NEG	NEG	NEG
Beaver D_Berlin_WT69	NEG	NEG																				
Beaver F_Berlin_WT71	NEG	NEG																				
Beaver E_Berlin_WT70	NEG	NEG																				
Beaver G_Berlin_WT110	NEG	NEG																				
Beaver G_Berlin_WT111	NEG	NEG																				

STRAIN / ISOLATE	RESISTANCE : PENICILLINASE			RESISTANCE : MLS-ANTIBIOTICS																
							ermC													
	blaZ	blaI	blaR	ermA	ermA-43	ermB	ermC	ermC-GM	ermF	ermT	linA/lnuA	lnuB	lsa-B	lsa-E	msrA	mefA	mph(C)	vat(A)	vat(B)	vga(A)
	beta-lactamase	beta lactamase repressor (inhibitor)	beta-lactamase regulatory protein	rRNA adenine N-6-methyl-transferase, erythromycin/clindamycin resistance		erythro-mycin/clindamycin resistance	0,00	rRNA adenine methylase-like protein ErmGM	rRNA adenine N-6-methyltransferase	0,00	Lincosamid-Nucleotidyltransferase	lincosamide nucleotidyltransferase	lincosamide ABC transporter	lincosamide ABC transporter	energy-dependent efflux of erythro-mycin	macrolide efflux protein A	probable lysylphos-phatidyl-glycerol synthetase	virginia-mycin A acetyltransferase	acetyl-transferase inactivating streptogramin A	ATP binding protein, streptogramin-A-resistance
**>CC8-MSSA**																				
Strain NCTC8325: in silico predicted hybridisation pattern	NEG	NEG	NEG	NEG	NEG	NEG	NEG	NEG	NEG	NEG	NEG	NEG	NEG	NEG	NEG	NEG	NEG	NEG	NEG	NEG
Strain RN4220:VC40: in silico predicted hybridisation pattern	NEG	NEG	NEG	NEG	NEG	NEG	NEG	NEG	NEG	NEG	NEG	NEG	NEG	NEG	NEG	NEG	NEG	NEG	NEG	NEG
Strain Newman: in silico predicted hybridisation pattern	NEG	NEG	NEG	NEG	NEG	NEG	NEG	NEG	NEG	NEG	NEG	NEG	NEG	NEG	NEG	NEG	NEG	NEG	NEG	NEG
Beaver I_Austria_B2	NEG	NEG	NEG	NEG		NEG	POS				NEG				NEG	NEG	NEG	NEG	NEG	NEG
Beaver J_Austria_B3	POS	POS	POS	NEG		NEG	NEG				NEG				NEG	NEG	NEG	NEG	NEG	NEG
**>CC12-MSSA**																				
Strain KLT6: in silico predicted hybridisation pattern	NEG	NEG	NEG	NEG	NEG	NEG	NEG	NEG	NEG	NEG	NEG	NEG	NEG	NEG	NEG	NEG	NEG	NEG	NEG	NEG
Strain 21266: in silico predicted hybridisation pattern	POS	POS	POS	NEG	NEG	NEG	NEG	NEG	NEG	NEG	NEG	NEG	NEG	NEG	NEG	NEG	NEG	NEG	NEG	NEG
NP66 chromosome: in silico predicted hybridisation pattern	POS	POS	AMB	NEG	NEG	NEG	NEG	NEG	NEG	NEG	NEG	NEG	NEG	NEG	NEG	NEG	NEG	NEG	NEG	NEG
Beaver K_Austria_B4	POS	POS	AMB	NEG		NEG	NEG				NEG				NEG	NEG	NEG	NEG	NEG	NEG
**>CC49-MSSA**																				
Strain 21262: in silico predicted hybridisation pattern	NEG	NEG	NEG	NEG	NEG	NEG	POS	NEG	NEG	NEG	NEG	NEG	NEG	NEG	NEG	NEG	NEG	NEG	NEG	NEG
Strain Tager 104: in silico predicted hybridisation pattern	NEG	NEG	NEG	NEG	NEG	NEG	NEG	NEG	NEG	NEG	NEG	NEG	NEG	NEG	NEG	NEG	NEG	NEG	NEG	NEG
**>CC49-MSSA (lukF-P83/lukM+)**																				
Strain 22_M61_07_10: in silico predicted hybridisation pattern	NEG	NEG	NEG	NEG	NEG	NEG	NEG	NEG	NEG	NEG	NEG	NEG	NEG	NEG	NEG	NEG	NEG	NEG	NEG	NEG
Strain 22_M48_10_10: in silico predicted hybridisation pattern	NEG	NEG	NEG	NEG	NEG	NEG	NEG	NEG	NEG	NEG	NEG	NEG	NEG	NEG	NEG	NEG	NEG	NEG	NEG	NEG
Strain 22_M47_10_10: in silico predicted hybridisation pattern	NEG	NEG	NEG	NEG	NEG	NEG	NEG	NEG	NEG	NEG	NEG	NEG	NEG	NEG	NEG	NEG	NEG	NEG	NEG	NEG
**>CC49-MSSA (lukF-PV+/lukS-PV?)**																				
Beaver C_Bavaria_WT65: in silico predicted hybridisation pattern	NEG	NEG	NEG	NEG	NEG	NEG	NEG	NEG	NEG	NEG	NEG	NEG	NEG	NEG	NEG	NEG	NEG	NEG	NEG	NEG
Beaver C_Bavaria_WT65	NEG	NEG	NEG	NEG	NEG	NEG	NEG	NEG	NEG	NEG	NEG	NEG	NEG	NEG	NEG	NEG	NEG	NEG	NEG	NEG
**>CC398-MSSA**																				
Strain 21331: in silico predicted hybridisation pattern	NEG	NEG	NEG	NEG	NEG	NEG	NEG	NEG	NEG	POS	NEG	NEG	NEG	NEG	NEG	NEG	NEG	NEG	NEG	NEG
Strain 71193: in silico predicted hybridisation pattern	POS	POS	POS	NEG	NEG	NEG	NEG	NEG	NEG	POS	NEG	NEG	NEG	NEG	NEG	NEG	NEG	NEG	NEG	NEG
Strain S123: in silico predicted hybridisation pattern	POS	POS	POS	NEG	NEG	POS	NEG	NEG	NEG	NEG	NEG	NEG	NEG	NEG	NEG	NEG	NEG	NEG	NEG	NEG
Beaver H_Austria_B1	POS	POS	POS	POS		NEG	NEG				NEG				NEG	NEG	NEG	NEG	NEG	NEG
**>CC1956-MSSA**																				
Strain C6589: in silico predicted hybridisation pattern	NEG	NEG	NEG	NEG	NEG	NEG	NEG	NEG	NEG	NEG	NEG	NEG	NEG	NEG	NEG	NEG	NEG	NEG	NEG	NEG
Strain C6591: in silico predicted hybridisation pattern	NEG	NEG	NEG	NEG	NEG	NEG	NEG	NEG	NEG	NEG	NEG	NEG	NEG	NEG	NEG	NEG	NEG	NEG	NEG	NEG
Strain C6597: in silico predicted hybridisation pattern	NEG	NEG	NEG	NEG	NEG	NEG	NEG	NEG	NEG	NEG	NEG	NEG	NEG	NEG	NEG	NEG	NEG	NEG	NEG	NEG
Strain C6598: in silico predicted hybridisation pattern	NEG	NEG	NEG	NEG	NEG	NEG	NEG	NEG	NEG	NEG	NEG	NEG	NEG	NEG	NEG	NEG	NEG	NEG	NEG	NEG
Strain C6601: in silico predicted hybridisation pattern	NEG	NEG	NEG	NEG	NEG	NEG	NEG	NEG	NEG	NEG	NEG	NEG	NEG	NEG	NEG	NEG	NEG	NEG	NEG	NEG
**>CC1956-MSSA (lukF-PV+/lukS-PV?)**																				
Beaver A_Berlin_WT19: in silico predicted hybridisation pattern	NEG	NEG	NEG	NEG	NEG	NEG	NEG	NEG	NEG	NEG	NEG	NEG	NEG	NEG	NEG	NEG	NEG	NEG	NEG	NEG
Beaver A_Berlin_WT19	NEG	NEG	NEG	NEG	NEG	NEG	NEG	NEG	NEG	NEG	NEG	NEG	NEG	NEG	NEG	NEG	NEG	NEG	NEG	NEG
Beaver B_Berlin_WT63	NEG	NEG	NEG	NEG	NEG	NEG	NEG	NEG	NEG	NEG	NEG	NEG	NEG	NEG	NEG	NEG	NEG	NEG	NEG	NEG
Beaver B_Berlin_WT64	NEG	NEG	NEG	NEG		NEG	NEG				NEG				NEG	NEG	NEG	NEG	NEG	NEG
Beaver D_Berlin_WT66	NEG	NEG	NEG	NEG		NEG	NEG				NEG				NEG	NEG	NEG	NEG	NEG	NEG
Beaver D_Berlin_WT67A	NEG	NEG	NEG	NEG		NEG	NEG				NEG				NEG	NEG	NEG	NEG	NEG	NEG
Beaver D_Berlin_WT67B	NEG	NEG	NEG	NEG	NEG	NEG	NEG	NEG	NEG	NEG	NEG	NEG	NEG	NEG	NEG	NEG	NEG	NEG	NEG	NEG
Beaver D_Berlin_WT68	NEG	NEG	NEG	NEG	NEG	NEG	NEG	NEG	NEG	NEG	NEG	NEG	NEG	NEG	NEG	NEG	NEG	NEG	NEG	NEG
Beaver D_Berlin_WT69	NEG	NEG	NEG	NEG		NEG	NEG				NEG				NEG	NEG	NEG	NEG	NEG	NEG
Beaver F_Berlin_WT71	NEG	NEG	NEG	NEG		NEG	NEG				NEG				NEG	NEG	NEG	NEG	NEG	NEG
Beaver E_Berlin_WT70	NEG	NEG	NEG	NEG		NEG	NEG				NEG				NEG	NEG	NEG	NEG	NEG	NEG
Beaver G_Berlin_WT110	NEG	NEG	NEG	NEG		NEG	NEG				NEG				NEG	NEG	NEG	NEG	NEG	NEG
Beaver G_Berlin_WT111	NEG	NEG	NEG	NEG		NEG	NEG				NEG				NEG	NEG	NEG	NEG	NEG	NEG

STRAIN / ISOLATE	RESISTANCE : MLS-ANTIBIOTICS						RESISTANCE : AMINOGLYSOSIDES										
									ant9				aadA		aadE		
	vga(A) (BM 3327)	vgaB	vgaC	vgaD	vgaE	vgb	aacA-aphD	aadD	ant9-C2944	ant9	ble	aad6	aadA-var1	aadA-var2	aadE-C2944	aadE	aphA3
		streptogramin A ABC	streptogramin A ABC transporter		streptogramin A ABC	virginia-mycin B hydrolase	bifunctional enzyme Aac/Aph, gentamicin resistance	amino-glycoside adenyl-transferase,tobramycin resistance	adenyltransferase AAd9		bleomycin resistance protein	aminoglycoside 6-adenylyltransferase	aminoglycoside-3'-adenylyltransferase		streptomycin aminoglycoside 6-adenylyltransferase		3'5'-aminog-lycoside phospho-transferase, neo-/ kanamycin resistance
**>CC8-MSSA**																	
Strain NCTC8325: in silico predicted hybridisation pattern	NEG	NEG	NEG	NEG	NEG	NEG	NEG	NEG	NEG	NEG	NEG	NEG	NEG	NEG	NEG	NEG	NEG
Strain RN4220:VC40: in silico predicted hybridisation pattern	NEG	NEG	NEG	NEG	NEG	NEG	NEG	NEG	NEG	NEG	NEG	NEG	NEG	NEG	NEG	NEG	NEG
Strain Newman: in silico predicted hybridisation pattern	NEG	NEG	NEG	NEG	NEG	NEG	NEG	NEG	NEG	NEG	NEG	NEG	NEG	NEG	NEG	NEG	NEG
Beaver I_Austria_B2	NEG					NEG	NEG	NEG									NEG
Beaver J_Austria_B3	NEG					NEG	NEG	NEG									NEG
**>CC12-MSSA**																	
Strain KLT6: in silico predicted hybridisation pattern	NEG	NEG	NEG	NEG	NEG	NEG	NEG	NEG	NEG	NEG	NEG	NEG	NEG	NEG	NEG	NEG	NEG
Strain 21266: in silico predicted hybridisation pattern	NEG	NEG	NEG	NEG	NEG	NEG	NEG	NEG	NEG	NEG	NEG	NEG	NEG	NEG	NEG	NEG	NEG
NP66 chromosome: in silico predicted hybridisation pattern	NEG	NEG	NEG	NEG	NEG	NEG	NEG	NEG	NEG	NEG	NEG	NEG	NEG	NEG	NEG	NEG	NEG
Beaver K_Austria_B4	NEG					NEG	NEG	NEG									NEG
**>CC49-MSSA**																	
Strain 21262: in silico predicted hybridisation pattern	NEG	NEG	NEG	NEG	NEG	NEG	NEG	NEG	NEG	NEG	NEG	NEG	NEG	NEG	NEG	NEG	NEG
Strain Tager 104: in silico predicted hybridisation pattern	NEG	NEG	NEG	NEG	NEG	NEG	NEG	NEG	NEG	NEG	NEG	NEG	NEG	NEG	NEG	NEG	NEG
**>CC49-MSSA (lukF-P83/lukM+)**																	
Strain 22_M61_07_10: in silico predicted hybridisation pattern	NEG	NEG	NEG	NEG	NEG	NEG	NEG	NEG	NEG	NEG	NEG	NEG	NEG	NEG	NEG	NEG	NEG
Strain 22_M48_10_10: in silico predicted hybridisation pattern	NEG	NEG	NEG	NEG	NEG	NEG	NEG	NEG	NEG	NEG	NEG	NEG	NEG	NEG	NEG	NEG	NEG
Strain 22_M47_10_10: in silico predicted hybridisation pattern	NEG	NEG	NEG	NEG	NEG	NEG	NEG	NEG	NEG	NEG	NEG	NEG	NEG	NEG	NEG	NEG	NEG
**>CC49-MSSA (lukF-PV+/lukS-PV?)**																	
Beaver C_Bavaria_WT65: in silico predicted hybridisation pattern	NEG	NEG	NEG	NEG	NEG	NEG	NEG	NEG	NEG	NEG	NEG	NEG	NEG	NEG	NEG	NEG	NEG
Beaver C_Bavaria_WT65	NEG	NEG	NEG	NEG	NEG	NEG	NEG	NEG	NEG	NEG	NEG	NEG	NEG	NEG	NEG	NEG	NEG
**>CC398-MSSA**																	
Strain 21331: in silico predicted hybridisation pattern	NEG	NEG	NEG	NEG	NEG	NEG	NEG	NEG	NEG	NEG	NEG	NEG	NEG	NEG	NEG	NEG	NEG
Strain 71193: in silico predicted hybridisation pattern	NEG	NEG	NEG	NEG	NEG	NEG	NEG	NEG	NEG	NEG	NEG	NEG	NEG	NEG	NEG	NEG	NEG
Strain S123: in silico predicted hybridisation pattern	NEG	NEG	NEG	NEG	NEG	NEG	NEG	NEG	NEG	NEG	NEG	POS	NEG	NEG	NEG	NEG	NEG
Beaver H_Austria_B1	NEG					NEG	NEG	NEG									NEG
**>CC1956-MSSA**																	
Strain C6589: in silico predicted hybridisation pattern	NEG	NEG	NEG	NEG	NEG	NEG	NEG	NEG	NEG	NEG	NEG	NEG	NEG	NEG	NEG	NEG	NEG
Strain C6591: in silico predicted hybridisation pattern	NEG	NEG	NEG	NEG	NEG	NEG	NEG	NEG	NEG	NEG	NEG	NEG	NEG	NEG	NEG	NEG	NEG
Strain C6597: in silico predicted hybridisation pattern	NEG	NEG	NEG	NEG	NEG	NEG	NEG	NEG	NEG	NEG	NEG	NEG	NEG	NEG	NEG	NEG	NEG
Strain C6598: in silico predicted hybridisation pattern	NEG	NEG	NEG	NEG	NEG	NEG	NEG	NEG	NEG	NEG	NEG	NEG	NEG	NEG	NEG	NEG	NEG
Strain C6601: in silico predicted hybridisation pattern	NEG	NEG	NEG	NEG	NEG	NEG	NEG	NEG	NEG	NEG	NEG	NEG	NEG	NEG	NEG	NEG	NEG
**>CC1956-MSSA (lukF-PV+/lukS-PV?)**																	
Beaver A_Berlin_WT19: in silico predicted hybridisation pattern	NEG	NEG	NEG	NEG	NEG	NEG	NEG	NEG	NEG	NEG	NEG	NEG	NEG	NEG	NEG	NEG	NEG
Beaver A_Berlin_WT19	NEG	NEG	NEG	NEG	NEG	NEG	NEG	NEG	NEG	NEG	NEG	NEG	NEG	NEG	NEG	NEG	NEG
Beaver B_Berlin_WT63	NEG	NEG	NEG	NEG	NEG	NEG	NEG	NEG	NEG	NEG	NEG	NEG	NEG	NEG	NEG	NEG	NEG
Beaver B_Berlin_WT64	NEG					NEG	NEG	NEG									NEG
Beaver D_Berlin_WT66	NEG					NEG	NEG	NEG									NEG
Beaver D_Berlin_WT67A	NEG					NEG	NEG	NEG									NEG
Beaver D_Berlin_WT67B	NEG	NEG	NEG	NEG	NEG	NEG	NEG	NEG	NEG	NEG	NEG	NEG	NEG	NEG	NEG	NEG	NEG
Beaver D_Berlin_WT68	NEG	NEG	NEG	NEG	NEG	NEG	NEG	NEG	NEG	NEG	NEG	NEG	NEG	NEG	NEG	NEG	NEG
Beaver D_Berlin_WT69	NEG					NEG	NEG	NEG									NEG
Beaver F_Berlin_WT71	NEG					NEG	NEG	NEG									NEG
Beaver E_Berlin_WT70	NEG					NEG	NEG	NEG									NEG
Beaver G_Berlin_WT110	NEG					NEG	NEG	NEG									NEG
Beaver G_Berlin_WT111	NEG					NEG	NEG	NEG									NEG

STRAIN / ISOLATE	RESISTANCE : MISCELLANEOUS GENES																					
									tetM													
	sat	dfrA	dfrG	far1	mupA	mupB	tetK	tetL	tetM (com-bined)	tetM-O	tetM-S	cat	cfr	fexA	apmA	fosB	qacA	qacC	smr	vanA	vanB	vanZ
	strepto-thricine-acetyl-transferase	dihydro-folate reductase type 1	dihydrofolate reductase	fusidic acid resistence	mupirocin resistence protein	plasmidic isoleucyl-tR	tetracycline resistance	Tetracycline resistanc	tetracycline resistance			chloramphenicol acetyltransferase	23S rRNA methyltransferase	chloramphenicol/florfenicol exporter	aminocyclitol acetyltr	metallothiol transferase	quaternary ammonium compound resistance protein A	quaternary ammonium compound resistance protein C		vancomycin resistance gene	vancomycin resistance gene from enterococci and Clostridium	teicoplanin resistance gene from enterococci
**>CC8-MSSA**																						
Strain NCTC8325: in silico predicted hybridisation pattern	NEG	NEG	NEG	NEG	NEG	NEG	NEG	NEG	NEG	NEG	NEG	NEG	NEG	NEG	NEG	POS	NEG	NEG	NEG	NEG	NEG	NEG
Strain RN4220:VC40: in silico predicted hybridisation pattern	NEG	NEG	NEG	NEG	NEG	NEG	NEG	NEG	NEG	NEG	NEG	POS	NEG	NEG	NEG	POS	NEG	NEG	NEG	NEG	NEG	NEG
Strain Newman: in silico predicted hybridisation pattern	NEG	NEG	NEG	NEG	NEG	NEG	NEG	NEG	NEG	NEG	NEG	NEG	NEG	NEG	NEG	POS	NEG	NEG	NEG	NEG	NEG	NEG
Beaver I_Austria_B2	NEG	NEG		NEG	NEG		NEG		NEG			NEG	NEG	NEG		POS	NEG	NEG		NEG	NEG	NEG
Beaver J_Austria_B3	NEG	NEG		NEG	NEG		NEG		NEG			NEG	NEG	NEG		POS	NEG	NEG		NEG	NEG	NEG
**>CC12-MSSA**																						
Strain KLT6: in silico predicted hybridisation pattern	NEG	NEG	NEG	NEG	NEG	NEG	NEG	NEG	NEG	NEG	NEG	NEG	NEG	NEG	NEG	POS	NEG	NEG	NEG	NEG	NEG	NEG
Strain 21266: in silico predicted hybridisation pattern	NEG	NEG	NEG	NEG	NEG	NEG	NEG	NEG	NEG	NEG	NEG	NEG	NEG	NEG	NEG	POS	NEG	NEG	NEG	NEG	NEG	NEG
NP66 chromosome: in silico predicted hybridisation pattern	NEG	NEG	NEG	NEG	NEG	NEG	NEG	NEG	NEG	NEG	NEG	NEG	NEG	NEG	NEG	POS	NEG	NEG	NEG	NEG	NEG	NEG
Beaver K_Austria_B4	NEG	NEG		NEG	NEG		NEG		NEG			NEG	NEG	NEG		POS	NEG	NEG		NEG	NEG	NEG
**>CC49-MSSA**																						
Strain 21262: in silico predicted hybridisation pattern	NEG	NEG	NEG	NEG	NEG	NEG	NEG	NEG	NEG	NEG	NEG	NEG	NEG	NEG	NEG	NEG	NEG	NEG	NEG	NEG	NEG	NEG
Strain Tager 104: in silico predicted hybridisation pattern	NEG	NEG	NEG	NEG	NEG	NEG	NEG	NEG	NEG	NEG	NEG	NEG	NEG	NEG	NEG	NEG	NEG	NEG	NEG	NEG	NEG	NEG
**>CC49-MSSA (lukF-P83/lukM+)**																						
Strain 22_M61_07_10: in silico predicted hybridisation pattern	NEG	NEG	NEG	NEG	NEG	NEG	NEG	NEG	NEG	NEG	NEG	NEG	NEG	NEG	NEG	NEG	NEG	NEG	NEG	NEG	NEG	NEG
Strain 22_M48_10_10: in silico predicted hybridisation pattern	NEG	NEG	NEG	NEG	NEG	NEG	NEG	NEG	NEG	NEG	NEG	NEG	NEG	NEG	NEG	NEG	NEG	NEG	NEG	NEG	NEG	NEG
Strain 22_M47_10_10: in silico predicted hybridisation pattern	NEG	NEG	NEG	NEG	NEG	NEG	NEG	NEG	NEG	NEG	NEG	NEG	NEG	NEG	NEG	NEG	NEG	NEG	NEG	NEG	NEG	NEG
**>CC49-MSSA (lukF-PV+/lukS-PV?)**																						
Beaver C_Bavaria_WT65: in silico predicted hybridisation pattern	NEG	NEG	NEG	NEG	NEG	NEG	NEG	NEG	NEG	NEG	NEG	NEG	NEG	NEG	NEG	NEG	NEG	NEG	NEG	NEG	NEG	NEG
Beaver C_Bavaria_WT65	NEG	NEG	NEG	NEG	NEG	NEG	NEG	NEG	NEG	NEG	NEG	NEG	NEG	NEG	NEG	NEG	NEG	NEG	NEG	NEG	NEG	NEG
**>CC398-MSSA**																						
Strain 21331: in silico predicted hybridisation pattern	NEG	NEG	NEG	NEG	NEG	NEG	NEG	NEG	NEG	NEG	NEG	NEG	NEG	NEG	NEG	NEG	NEG	NEG	NEG	NEG	NEG	NEG
Strain 71193: in silico predicted hybridisation pattern	NEG	NEG	NEG	NEG	NEG	NEG	NEG	NEG	NEG	NEG	NEG	NEG	NEG	NEG	NEG	NEG	NEG	NEG	NEG	NEG	NEG	NEG
Strain S123: in silico predicted hybridisation pattern	NEG	NEG	NEG	NEG	NEG	NEG	NEG	NEG	POS	NEG	NEG	NEG	NEG	NEG	NEG	NEG	NEG	NEG	NEG	NEG	NEG	NEG
Beaver H_Austria_B1	NEG	NEG		NEG	NEG		NEG		POS			NEG	NEG	NEG		NEG	NEG	NEG		NEG	NEG	NEG
**>CC1956-MSSA**																						
Strain C6589: in silico predicted hybridisation pattern	NEG	NEG	NEG	NEG	NEG	NEG	NEG	NEG	NEG	NEG	NEG	NEG	NEG	NEG	NEG	POS	NEG	NEG	NEG	NEG	NEG	NEG
Strain C6591: in silico predicted hybridisation pattern	NEG	NEG	NEG	NEG	NEG	NEG	NEG	NEG	NEG	NEG	NEG	NEG	NEG	NEG	NEG	POS	NEG	NEG	NEG	NEG	NEG	NEG
Strain C6597: in silico predicted hybridisation pattern	NEG	NEG	NEG	NEG	NEG	NEG	NEG	NEG	NEG	NEG	NEG	NEG	NEG	NEG	NEG	POS	NEG	NEG	NEG	NEG	NEG	NEG
Strain C6598: in silico predicted hybridisation pattern	NEG	NEG	NEG	NEG	NEG	NEG	NEG	NEG	NEG	NEG	NEG	NEG	NEG	NEG	NEG	POS	NEG	NEG	NEG	NEG	NEG	NEG
Strain C6601: in silico predicted hybridisation pattern	NEG	NEG	NEG	NEG	NEG	NEG	NEG	NEG	NEG	NEG	NEG	NEG	NEG	NEG	NEG	POS	NEG	NEG	NEG	NEG	NEG	NEG
**>CC1956-MSSA (lukF-PV+/lukS-PV?)**																						
Beaver A_Berlin_WT19: in silico predicted hybridisation pattern	NEG	NEG	NEG	NEG	NEG	NEG	NEG	NEG	NEG	NEG	NEG	NEG	NEG	NEG	NEG	POS	NEG	NEG	NEG	NEG	NEG	NEG
Beaver A_Berlin_WT19	NEG	NEG	NEG	NEG	NEG	NEG	NEG	NEG	NEG	NEG	NEG	NEG	NEG	NEG	NEG	POS	NEG	NEG	NEG	NEG	NEG	NEG
Beaver B_Berlin_WT63	NEG	NEG	NEG	NEG	NEG	NEG	NEG	NEG	NEG	NEG	NEG	NEG	NEG	NEG	NEG	POS	NEG	NEG	NEG	NEG	NEG	NEG
Beaver B_Berlin_WT64	NEG	NEG		NEG	NEG		NEG		NEG			NEG	NEG	NEG		POS	NEG	NEG		NEG	NEG	NEG
Beaver D_Berlin_WT66	NEG	NEG		NEG	NEG		NEG		NEG			NEG	NEG	NEG		POS	NEG	NEG		NEG	NEG	NEG
Beaver D_Berlin_WT67A	NEG	NEG		NEG	NEG		NEG		NEG			NEG	NEG	NEG		POS	NEG	NEG		NEG	NEG	NEG
Beaver D_Berlin_WT67B	NEG	NEG	NEG	NEG	NEG	NEG	NEG	NEG	NEG	NEG	NEG	NEG	NEG	NEG	NEG	POS	NEG	NEG	NEG	NEG	NEG	NEG
Beaver D_Berlin_WT68	NEG	NEG	NEG	NEG	NEG	NEG	NEG	NEG	NEG	NEG	NEG	NEG	NEG	NEG	NEG	POS	NEG	NEG	NEG	NEG	NEG	NEG
Beaver D_Berlin_WT69	NEG	NEG		NEG	NEG		NEG		NEG			NEG	NEG	NEG		POS	NEG	NEG		NEG	NEG	NEG
Beaver F_Berlin_WT71	NEG	NEG		NEG	NEG		NEG		NEG			NEG	NEG	NEG		POS	NEG	NEG		NEG	NEG	NEG
Beaver E_Berlin_WT70	NEG	NEG		NEG	NEG		NEG		NEG			NEG	NEG	NEG		POS	NEG	NEG		NEG	NEG	NEG
Beaver G_Berlin_WT110	NEG	NEG		NEG	NEG		NEG		NEG			NEG	NEG	NEG		POS	NEG	NEG		NEG	NEG	NEG
Beaver G_Berlin_WT111	NEG	NEG		NEG	NEG		NEG		NEG			NEG	NEG	NEG		POS	NEG	NEG		NEG	NEG	NEG

STRAIN / ISOLATE	VIRULENCE : TOX.SCHOCK.T.			VIRULENCE : ENTEROTOXINS																		
	tst1			seA			seB	seC	seD	seD2	seE	seH	seJ	seK	seL	seN2	seQ	seR	seS	seT	seU2	seW
	tst1 (consensus)	tst1 ("human" allele)	tst1 ("bovine" allele)	entA	entA (320E)	entA (N315) / entP																
	toxic shock syndrome toxin 1			Enterotoxin A	Enterotoxin A, allele from strain 320E	Enterotoxin A, allele from strain N315 = Entero-toxin P	Enterotoxin B	Enterotoxin C	Enterotoxin D	Enterotoxin similar to EntD	Enterotoxin E	Enterotoxin H	Enterotoxin J	Enterotoxin K	Enterotoxin L	Putative Enterotoxin N2	Enterotoxin Q	Enterotoxin R	Enterotoxin S	Enterotoxin T	Enterotoxin U2	Putative Enterotoxin
**>CC8-MSSA**																						
Strain NCTC8325: in silico predicted hybridisation pattern	NEG	NEG	NEG	NEG	NEG	NEG	NEG	NEG	NEG	NEG	NEG	NEG	NEG	NEG	NEG	NEG	NEG	NEG	NEG	NEG	NEG	NEG
Strain RN4220:VC40: in silico predicted hybridisation pattern	NEG	NEG	NEG	NEG	NEG	NEG	NEG	NEG	NEG	NEG	NEG	NEG	NEG	NEG	NEG	NEG	NEG	NEG	NEG	NEG	NEG	NEG
Strain Newman: in silico predicted hybridisation pattern	NEG	NEG	NEG	POS	NEG	NEG	NEG	NEG	NEG	NEG	NEG	NEG	NEG	NEG	NEG	NEG	NEG	NEG	NEG	NEG	NEG	NEG
Beaver I_Austria_B2	NEG	NEG	NEG	NEG	NEG	NEG	NEG	NEG	NEG		NEG	NEG	NEG	NEG	NEG		NEG	NEG				
Beaver J_Austria_B3	NEG	NEG	NEG	NEG	NEG	NEG	NEG	NEG	POS		NEG	NEG	POS	NEG	NEG		NEG	POS				
**>CC12-MSSA**																						
Strain KLT6: in silico predicted hybridisation pattern	NEG	NEG	NEG	NEG	NEG	NEG	POS	NEG	NEG	NEG	NEG	NEG	NEG	NEG	NEG	NEG	NEG	NEG	NEG	NEG	NEG	NEG
Strain 21266: in silico predicted hybridisation pattern	NEG	NEG	NEG	NEG	NEG	POS	POS	NEG	NEG	NEG	NEG	NEG	NEG	NEG	NEG	NEG	NEG	NEG	NEG	NEG	NEG	NEG
NP66 chromosome: in silico predicted hybridisation pattern	NEG	NEG	NEG	NEG	NEG	POS	NEG	POS	NEG	NEG	NEG	NEG	NEG	NEG	POS	NEG	NEG	NEG	NEG	NEG	NEG	NEG
Beaver K_Austria_B4	NEG	NEG	NEG	NEG	NEG	POS	NEG	NEG	NEG		NEG	NEG	NEG	NEG	NEG		NEG	NEG				
**>CC49-MSSA**																						
Strain 21262: in silico predicted hybridisation pattern	POS	POS	NEG	NEG	NEG	POS	NEG	POS	NEG	NEG	NEG	NEG	NEG	NEG	POS	NEG	NEG	NEG	NEG	NEG	NEG	NEG
Strain Tager 104: in silico predicted hybridisation pattern	NEG	NEG	NEG	NEG	NEG	POS	NEG	NEG	NEG	NEG	NEG	NEG	NEG	NEG	NEG	NEG	NEG	NEG	NEG	NEG	NEG	NEG
**>CC49-MSSA (lukF-P83/lukM+)**																						
Strain 22_M61_07_10: in silico predicted hybridisation pattern	NEG	NEG	NEG	NEG	NEG	NEG	NEG	NEG	NEG	NEG	NEG	NEG	NEG	NEG	NEG	NEG	NEG	NEG	NEG	NEG	NEG	NEG
Strain 22_M48_10_10: in silico predicted hybridisation pattern	NEG	NEG	NEG	NEG	NEG	NEG	NEG	NEG	NEG	NEG	NEG	NEG	NEG	NEG	NEG	NEG	NEG	NEG	NEG	NEG	NEG	NEG
Strain 22_M47_10_10: in silico predicted hybridisation pattern	NEG	NEG	NEG	NEG	NEG	NEG	NEG	NEG	NEG	NEG	NEG	NEG	NEG	NEG	NEG	NEG	NEG	NEG	NEG	NEG	NEG	NEG
**>CC49-MSSA (lukF-PV+/lukS-PV?)**																						
Beaver C_Bavaria_WT65: in silico predicted hybridisation pattern	NEG	NEG	NEG	NEG	NEG	NEG	NEG	NEG	NEG	NEG	NEG	NEG	NEG	NEG	NEG	NEG	NEG	NEG	NEG	NEG	NEG	NEG
Beaver C_Bavaria_WT65	NEG	NEG	NEG	NEG	NEG	NEG	NEG	NEG	NEG	NEG	NEG	NEG	NEG	NEG	NEG	NEG	NEG	NEG	NEG	NEG	NEG	NEG
**>CC398-MSSA**																						
Strain 21331: in silico predicted hybridisation pattern	NEG	NEG	NEG	NEG	NEG	NEG	NEG	NEG	NEG	NEG	NEG	NEG	NEG	NEG	NEG	NEG	NEG	NEG	NEG	NEG	NEG	NEG
Strain 71193: in silico predicted hybridisation pattern	NEG	NEG	NEG	NEG	NEG	NEG	NEG	NEG	NEG	NEG	NEG	NEG	NEG	NEG	NEG	NEG	NEG	NEG	NEG	NEG	NEG	NEG
Strain S123: in silico predicted hybridisation pattern	NEG	NEG	NEG	NEG	NEG	NEG	NEG	NEG	NEG	NEG	NEG	NEG	NEG	NEG	NEG	NEG	NEG	NEG	NEG	NEG	NEG	NEG
Beaver H_Austria_B1	NEG	NEG	NEG	NEG	NEG	NEG	NEG	NEG	NEG		NEG	NEG	NEG	NEG	NEG		NEG	NEG				
**>CC1956-MSSA**																						
Strain C6589: in silico predicted hybridisation pattern	NEG	NEG	NEG	NEG	NEG	NEG	NEG	NEG	NEG	NEG	NEG	NEG	NEG	NEG	NEG	NEG	NEG	NEG	NEG	NEG	POS	NEG
Strain C6591: in silico predicted hybridisation pattern	NEG	NEG	NEG	NEG	NEG	NEG	NEG	NEG	NEG	NEG	NEG	NEG	NEG	NEG	NEG	NEG	NEG	NEG	NEG	NEG	POS	NEG
Strain C6597: in silico predicted hybridisation pattern	NEG	NEG	NEG	NEG	NEG	NEG	NEG	NEG	NEG	NEG	NEG	NEG	NEG	NEG	NEG	NEG	NEG	NEG	NEG	NEG	POS	NEG
Strain C6598: in silico predicted hybridisation pattern	NEG	NEG	NEG	NEG	NEG	NEG	NEG	NEG	NEG	NEG	NEG	NEG	NEG	NEG	NEG	NEG	NEG	NEG	NEG	NEG	POS	NEG
Strain C6601: in silico predicted hybridisation pattern	NEG	NEG	NEG	NEG	NEG	NEG	NEG	NEG	NEG	NEG	NEG	NEG	NEG	NEG	NEG	NEG	NEG	NEG	NEG	NEG	POS	NEG
**>CC1956-MSSA (lukF-PV+/lukS-PV?)**																						
Beaver A_Berlin_WT19: in silico predicted hybridisation pattern	NEG	NEG	NEG	NEG	NEG	NEG	NEG	NEG	NEG	NEG	NEG	NEG	NEG	NEG	NEG	NEG	NEG	NEG	NEG	NEG	POS	NEG
Beaver A_Berlin_WT19	NEG	NEG	NEG	NEG	NEG	NEG	NEG	NEG	NEG	NEG	NEG	NEG	NEG	NEG	NEG	NEG	NEG	NEG	NEG	NEG	POS	NEG
Beaver B_Berlin_WT63	NEG	NEG	NEG	NEG	NEG	NEG	NEG	NEG	NEG	NEG	NEG	NEG	NEG	NEG	NEG	NEG	NEG	NEG	NEG	NEG	POS	NEG
Beaver B_Berlin_WT64	NEG	NEG	NEG	NEG	NEG	NEG	NEG	NEG	NEG		NEG	NEG	NEG	NEG	NEG		NEG	NEG				
Beaver D_Berlin_WT66	NEG	NEG	NEG	NEG	NEG	NEG	NEG	NEG	NEG		NEG	NEG	NEG	NEG	NEG		NEG	NEG				
Beaver D_Berlin_WT67A	NEG	NEG	NEG	NEG	NEG	NEG	NEG	NEG	NEG		NEG	NEG	NEG	NEG	NEG		NEG	NEG				
Beaver D_Berlin_WT67B	NEG	NEG	NEG	NEG	NEG	NEG	NEG	NEG	NEG	NEG	NEG	NEG	NEG	NEG	NEG	NEG	NEG	NEG	NEG	NEG	POS	NEG
Beaver D_Berlin_WT68	NEG	NEG	NEG	NEG	NEG	NEG	NEG	NEG	NEG	NEG	NEG	NEG	NEG	NEG	NEG	NEG	NEG	NEG	NEG	NEG	POS	NEG
Beaver D_Berlin_WT69	NEG	NEG	NEG	NEG	NEG	NEG	NEG	NEG	NEG		NEG	NEG	NEG	NEG	NEG		NEG	NEG				
Beaver F_Berlin_WT71	NEG	NEG	NEG	NEG	NEG	NEG	NEG	NEG	NEG		NEG	NEG	NEG	NEG	NEG		NEG	NEG				
Beaver E_Berlin_WT70	NEG	NEG	NEG	NEG	NEG	NEG	NEG	NEG	NEG		NEG	NEG	NEG	NEG	NEG		NEG	NEG				
Beaver G_Berlin_WT110	NEG	NEG	NEG	NEG	NEG	NEG	NEG	NEG	NEG		NEG	NEG	NEG	NEG	NEG		NEG	NEG				
Beaver G_Berlin_WT111	NEG	NEG	NEG	NEG	NEG	NEG	NEG	NEG	NEG		NEG	NEG	NEG	NEG	NEG		NEG	NEG				

STRAIN / ISOLATE	VIRULENCE : ENTEROTOXINS								VIRULENCE : HLG AND LEUKOCIDINS														
	egc (total)	selg	seli	selm	sen	selo	selu	ORF CM14	lukF	lukS		hlgA	lukF/S (int)	lukF-PV	lukS-PV	lukF-PV (P83)	lukM	lukD	lukE	lukX	lukY		
										lukS	lukS (ST22+ST45)										lukY	lukY (ST30+ST45)	lukY (ST1850)
	egc cluster	Enterotoxin G	Enterotoxin I	Enterotoxin M	Enterotoxin N	Enterotoxin O	Enterotoxin U and/or Y	Enterotoxin-like protein ORF CM14	Haemolysin gamma / leukocidin, component B	Haemolysin gamma / leukocidin, component C		Haemolysin gamma,	intermedius group leukocidin	Panton Valentine leukocidin F component	Panton Valentine leukocidin S component	F component from ruminant leukocidin	S component from ruminant leukocidin	leukocidin D component	leukocidin E component	leukocidin/ haemolysin toxin family protein	leukocidin/haemolysin toxin family protein		
**>CC8-MSSA**																							
Strain NCTC8325: in silico predicted hybridisation pattern	NEG	NEG	NEG	NEG	NEG	NEG	NEG	NEG	POS	POS	AMB	POS	NEG	NEG	NEG	NEG	NEG	POS	POS	POS	POS	NEG	NEG
Strain RN4220:VC40: in silico predicted hybridisation pattern	NEG	NEG	NEG	NEG	NEG	NEG	NEG	NEG	POS	POS	AMB	POS	NEG	NEG	NEG	NEG	NEG	POS	POS	POS	POS	NEG	NEG
Strain Newman: in silico predicted hybridisation pattern	NEG	NEG	NEG	NEG	NEG	NEG	NEG	NEG	POS	POS	AMB	POS	NEG	NEG	NEG	NEG	NEG	POS	POS	POS	POS	NEG	NEG
Beaver I_Austria_B2	NEG	NEG	NEG	NEG	NEG	NEG	NEG	NEG	POS	POS	POS	POS		NEG	NEG	NEG	NEG	POS	POS	POS	POS	NEG	
Beaver J_Austria_B3	NEG	NEG	NEG	NEG	NEG	NEG	NEG	NEG	POS	POS	POS	POS		NEG	NEG	NEG	NEG	POS	POS	POS	POS	NEG	
**>CC12-MSSA**																							
Strain KLT6: in silico predicted hybridisation pattern	NEG	NEG	NEG	NEG	NEG	NEG	NEG	POS	POS	POS	AMB	POS	NEG	NEG	NEG	NEG	NEG	POS	POS	POS	POS	NEG	NEG
Strain 21266: in silico predicted hybridisation pattern	NEG	NEG	NEG	NEG	NEG	NEG	NEG	POS	POS	POS	AMB	POS	NEG	NEG	NEG	NEG	NEG	POS	POS	POS	POS	NEG	NEG
NP66 chromosome: in silico predicted hybridisation pattern	NEG	NEG	NEG	NEG	NEG	NEG	NEG	POS	POS	POS	AMB	POS	NEG	NEG	NEG	NEG	NEG	POS	POS	POS	POS	NEG	NEG
Beaver K_Austria_B4	NEG	NEG	NEG	NEG	NEG	NEG	NEG	POS	POS	POS	POS	POS		NEG	NEG	NEG	NEG	POS	POS	POS	POS	NEG	
**>CC49-MSSA**																							
Strain 21262: in silico predicted hybridisation pattern	NEG	NEG	NEG	NEG	NEG	NEG	NEG	NEG	POS	POS	AMB	POS	NEG	NEG	NEG	NEG	NEG	POS	AMB	POS	AMB	NEG	NEG
Strain Tager 104: in silico predicted hybridisation pattern	NEG	NEG	NEG	NEG	NEG	NEG	NEG	NEG	POS	POS	AMB	POS	NEG	NEG	NEG	NEG	NEG	POS	AMB	POS	AMB	NEG	NEG
**>CC49-MSSA (lukF-P83/lukM+)**																							
Strain 22_M61_07_10: in silico predicted hybridisation pattern	NEG	NEG	NEG	NEG	NEG	NEG	NEG	NEG	POS	POS	AMB	POS	NEG	NEG	NEG	POS	POS	POS	AMB	POS	AMB	NEG	NEG
Strain 22_M48_10_10: in silico predicted hybridisation pattern	NEG	NEG	NEG	NEG	NEG	NEG	NEG	NEG	POS	POS	AMB	POS	NEG	NEG	NEG	POS	POS	POS	AMB	POS	AMB	NEG	NEG
Strain 22_M47_10_10: in silico predicted hybridisation pattern	NEG	NEG	NEG	NEG	NEG	NEG	NEG	NEG	POS	POS	AMB	POS	NEG	NEG	NEG	POS	POS	POS	AMB	POS	AMB	NEG	NEG
**>CC49-MSSA (lukF-PV+/lukS-PV?)**																							
Beaver C_Bavaria_WT65: in silico predicted hybridisation pattern	NEG	NEG	NEG	NEG	NEG	NEG	NEG	NEG	POS	POS	AMB	POS	NEG	POS	AMB	NEG	NEG	POS	AMB	POS	AMB	NEG	NEG
Beaver C_Bavaria_WT65	NEG	NEG	NEG	NEG	NEG	NEG	NEG	NEG	POS	POS	POS	POS	NEG	POS	POS	NEG	NEG	POS	POS	POS	POS	NEG	NEG
**>CC398-MSSA**																							
Strain 21331: in silico predicted hybridisation pattern	NEG	NEG	NEG	NEG	NEG	NEG	NEG	NEG	POS	POS	AMB	POS	NEG	NEG	NEG	NEG	NEG	NEG	NEG	POS	AMB	NEG	NEG
Strain 71193: in silico predicted hybridisation pattern	NEG	NEG	NEG	NEG	NEG	NEG	NEG	NEG	POS	POS	AMB	POS	NEG	NEG	NEG	NEG	NEG	NEG	NEG	POS	AMB	NEG	NEG
Strain S123: in silico predicted hybridisation pattern	NEG	NEG	NEG	NEG	NEG	NEG	NEG	NEG	POS	POS	AMB	POS	NEG	NEG	NEG	NEG	NEG	NEG	NEG	POS	AMB	NEG	NEG
Beaver H_Austria_B1	NEG	NEG	NEG	NEG	NEG	NEG	NEG	NEG	POS	POS	POS	POS		NEG	NEG	NEG	NEG	NEG	NEG	POS	POS	NEG	
**>CC1956-MSSA**																							
Strain C6589: in silico predicted hybridisation pattern	NEG	NEG	NEG	NEG	NEG	NEG	NEG	NEG	POS	POS	AMB	POS	NEG	NEG	NEG	NEG	NEG	POS	AMB	POS	POS	NEG	NEG
Strain C6591: in silico predicted hybridisation pattern	NEG	NEG	NEG	NEG	NEG	NEG	NEG	NEG	POS	POS	AMB	POS	NEG	NEG	NEG	NEG	NEG	POS	AMB	POS	POS	NEG	NEG
Strain C6597: in silico predicted hybridisation pattern	NEG	NEG	NEG	NEG	NEG	NEG	NEG	NEG	POS	POS	AMB	POS	NEG	NEG	NEG	NEG	NEG	POS	AMB	POS	POS	NEG	NEG
Strain C6598: in silico predicted hybridisation pattern	NEG	NEG	NEG	NEG	NEG	NEG	NEG	NEG	POS	POS	AMB	POS	NEG	NEG	NEG	NEG	NEG	POS	AMB	POS	POS	NEG	NEG
Strain C6601: in silico predicted hybridisation pattern	NEG	NEG	NEG	NEG	NEG	NEG	NEG	NEG	POS	POS	AMB	POS	NEG	NEG	NEG	NEG	NEG	POS	AMB	POS	POS	NEG	NEG
**>CC1956-MSSA (lukF-PV+/lukS-PV?)**																							
Beaver A_Berlin_WT19: in silico predicted hybridisation pattern	NEG	NEG	NEG	NEG	NEG	NEG	NEG	NEG	POS	POS	AMB	POS	NEG	POS	AMB	NEG	NEG	POS	AMB	POS	POS	NEG	NEG
Beaver A_Berlin_WT19	NEG	NEG	NEG	NEG	NEG	NEG	NEG	NEG	POS	POS	POS	POS	NEG	POS	POS	NEG	NEG	POS	POS	POS	POS	NEG	NEG
Beaver B_Berlin_WT63	NEG	NEG	NEG	NEG	NEG	NEG	NEG	NEG	POS	POS	POS	POS	NEG	POS	POS	NEG	NEG	POS	POS	POS	POS	NEG	NEG
Beaver B_Berlin_WT64	NEG	NEG	NEG	NEG	NEG	NEG	NEG	NEG	POS	POS	POS	POS		POS	POS	NEG	NEG	POS	POS	POS	POS	NEG	
Beaver D_Berlin_WT66	NEG	NEG	NEG	NEG	NEG	NEG	NEG	NEG	POS	POS	POS	POS		POS	POS	NEG	NEG	POS	POS	POS	POS	NEG	
Beaver D_Berlin_WT67A	NEG	NEG	NEG	NEG	NEG	NEG	NEG	NEG	POS	POS	POS	POS		POS	POS	NEG	NEG	POS	POS	POS	POS	NEG	
Beaver D_Berlin_WT67B	NEG	NEG	NEG	NEG	NEG	NEG	NEG	NEG	POS	POS	POS	POS	NEG	POS	AMB	NEG	NEG	POS	POS	POS	POS	NEG	NEG
Beaver D_Berlin_WT68	NEG	NEG	NEG	NEG	NEG	NEG	NEG	NEG	POS	POS	POS	POS	NEG	POS	POS	NEG	NEG	POS	POS	POS	POS	NEG	NEG
Beaver D_Berlin_WT69	NEG	NEG	NEG	NEG	NEG	NEG	NEG	NEG	POS	POS	POS	POS		POS	POS	NEG	NEG	POS	POS	POS	POS	NEG	
Beaver F_Berlin_WT71	NEG	NEG	NEG	NEG	NEG	NEG	NEG	NEG	POS	POS	POS	POS		POS	POS	NEG	NEG	POS	POS	POS	POS	NEG	
Beaver E_Berlin_WT70	NEG	NEG	NEG	NEG	NEG	NEG	NEG	NEG	POS	POS	POS	POS		POS	POS	NEG	NEG	POS	POS	POS	POS	NEG	
Beaver G_Berlin_WT110	NEG	NEG	NEG	NEG	NEG	NEG	NEG	NEG	POS	POS	NEG	POS		POS	NEG	NEG	NEG	POS	POS	POS	POS	NEG	
Beaver G_Berlin_WT111	NEG	NEG	NEG	NEG	NEG	NEG	NEG	NEG	POS	POS	NEG	POS		POS	NEG	NEG	NEG	POS	POS	POS	POS	NEG	

STRAIN / ISOLATE	VIRULENCE : HAEMOLYSINS								VIRULENCE : HLB-CONV PHAGES			VIRULENCE : OTHER FACTORS								
			hlIII		hlb															
	corB (=hl)	hla	hlIII (cons)	hlIII (other than RF122)	hlb-probe 1	hlb-probe 2	hlb-probe 3	un-truncated hlb	sak	chp	scn	etA	etB	etD	etE / "etD2"	edinA	edinB	edinC	esxA	esxB
	Putative membrane protein	Haemolysin alpha	Putative membrane protein		haemolysin beta				staphylo-kinase	chemotaxis-inhibiting protein (CHIPS)	Staphyl. Comple-ment inhibitor	exfoliative toxin serotype A	exfoliative toxin serotype B	exfoliative toxin D	exfoliative toxin D2	epidermal cell differen-tiation inhibitor	epidermal cell differen-tiation inhibitor B	epidermal cell differen-tiation inhibitor C	virulence factor esxA	virulence factor esxB
**>CC8-MSSA**																				
Strain NCTC8325: in silico predicted hybridisation pattern	POS	POS	POS	POS	POS	POS	POS	POS	POS	POS	POS	NEG	NEG	NEG	NEG	NEG	NEG	NEG	POS	POS
Strain RN4220:VC40: in silico predicted hybridisation pattern	POS	POS	POS	POS	POS	POS	POS	POS	POS	NEG	NEG	NEG	NEG	NEG	NEG	NEG	NEG	NEG	POS	POS
Strain Newman: in silico predicted hybridisation pattern	POS	POS	POS	POS	POS	POS	POS	POS	POS	POS	POS	NEG	NEG	NEG	NEG	NEG	NEG	NEG	POS	POS
Beaver I_Austria_B2	POS	POS	POS	POS	POS	POS	POS	NEG	POS	NEG	POS	NEG	NEG	NEG		NEG	NEG	NEG		
Beaver J_Austria_B3	POS	POS	POS	POS	POS	POS	POS	NEG	POS	NEG	POS	NEG	NEG	NEG		NEG	NEG	NEG		
**>CC12-MSSA**																				
Strain KLT6: in silico predicted hybridisation pattern	POS	POS	POS	POS	POS	POS	POS	POS	NEG	NEG	NEG	NEG	NEG	NEG	NEG	NEG	NEG	NEG	POS	NEG
Strain 21266: in silico predicted hybridisation pattern	POS	POS	POS	POS	POS	POS	POS	POS	POS	NEG	POS	NEG	NEG	NEG	NEG	NEG	NEG	NEG	POS	NEG
NP66 chromosome: in silico predicted hybridisation pattern	POS	POS	POS	POS	NEG	NEG	NEG	POS	POS	NEG	POS	NEG	NEG	NEG	NEG	NEG	NEG	NEG	POS	NEG
Beaver K_Austria_B4	POS	POS	POS	POS	POS	POS	POS	NEG	POS	NEG	POS	NEG	NEG	NEG		NEG	NEG	NEG		
**>CC49-MSSA**																				
Strain 21262: in silico predicted hybridisation pattern	POS	POS	POS	NEG	POS	POS	POS	AMB	POS	POS	POS	NEG	NEG	NEG	NEG	NEG	NEG	NEG	POS	NEG
Strain Tager 104: in silico predicted hybridisation pattern	POS	POS	POS	NEG	POS	POS	POS	AMB	POS	POS	POS	NEG	NEG	NEG	NEG	NEG	NEG	NEG	POS	NEG
**>CC49-MSSA (lukF-P83/lukM+)**																				
Strain 22_M61_07_10: in silico predicted hybridisation pattern	POS	POS	POS	NEG	POS	POS	POS	AMB	NEG	NEG	NEG	NEG	NEG	NEG	NEG	NEG	NEG	NEG	POS	NEG
Strain 22_M48_10_10: in silico predicted hybridisation pattern	POS	POS	POS	NEG	POS	POS	POS	AMB	NEG	NEG	NEG	NEG	NEG	NEG	NEG	NEG	NEG	NEG	POS	NEG
Strain 22_M47_10_10: in silico predicted hybridisation pattern	POS	POS	POS	NEG	POS	POS	POS	AMB	NEG	NEG	NEG	NEG	NEG	NEG	NEG	NEG	NEG	NEG	POS	NEG
**>CC49-MSSA (lukF-PV+/lukS-PV?)**																				
Beaver C_Bavaria_WT65: in silico predicted hybridisation pattern	POS	POS	POS	NEG	POS	POS	POS	AMB	NEG	NEG	NEG	NEG	NEG	NEG	NEG	NEG	NEG	NEG	POS	NEG
Beaver C_Bavaria_WT65	POS	POS	POS	NEG	POS	POS	POS	POS	NEG	NEG	NEG	NEG	NEG	NEG	NEG	NEG	NEG	NEG	POS	NEG
**>CC398-MSSA**																				
Strain 21331: in silico predicted hybridisation pattern	POS	POS	POS	POS	AMB	NEG	NEG	NEG	NEG	POS	POS	NEG	NEG	NEG	NEG	NEG	NEG	NEG	POS	NEG
Strain 71193: in silico predicted hybridisation pattern	POS	POS	POS	POS	AMB	NEG	NEG	NEG	NEG	POS	POS	NEG	NEG	NEG	NEG	NEG	NEG	NEG	POS	NEG
Strain S123: in silico predicted hybridisation pattern	POS	POS	POS	POS	NEG	NEG	NEG	NEG	NEG	NEG	NEG	NEG	NEG	NEG	NEG	NEG	NEG	NEG	POS	NEG
Beaver H_Austria_B1	POS	POS	POS	POS	NEG	NEG	NEG	POS	NEG	NEG	NEG	NEG	NEG	NEG		NEG	NEG	NEG		
**>CC1956-MSSA**																				
Strain C6589: in silico predicted hybridisation pattern	POS	POS	POS	NEG	POS	AMB	POS	POS	NEG	NEG	NEG	NEG	NEG	NEG	NEG	NEG	NEG	NEG	POS	NEG
Strain C6591: in silico predicted hybridisation pattern	POS	POS	POS	NEG	POS	AMB	POS	POS	NEG	NEG	NEG	NEG	NEG	NEG	NEG	NEG	NEG	NEG	POS	NEG
Strain C6597: in silico predicted hybridisation pattern	POS	POS	POS	NEG	POS	AMB	POS	POS	NEG	NEG	NEG	NEG	NEG	NEG	NEG	NEG	NEG	NEG	POS	NEG
Strain C6598: in silico predicted hybridisation pattern	POS	POS	POS	NEG	POS	AMB	POS	POS	NEG	NEG	NEG	NEG	NEG	NEG	NEG	NEG	NEG	NEG	POS	NEG
Strain C6601: in silico predicted hybridisation pattern	POS	POS	POS	NEG	POS	AMB	POS	POS	NEG	NEG	NEG	NEG	NEG	NEG	NEG	NEG	NEG	NEG	POS	NEG
**>CC1956-MSSA (lukF-PV+/lukS-PV?)**																				
Beaver A_Berlin_WT19: in silico predicted hybridisation pattern	POS	POS	POS	NEG	POS	AMB	POS	POS	NEG	NEG	NEG	NEG	NEG	NEG	NEG	NEG	NEG	NEG	POS	NEG
Beaver A_Berlin_WT19	POS	POS	POS	NEG	AMB	POS	POS	POS	NEG	NEG	NEG	NEG	NEG	NEG	NEG	NEG	NEG	NEG	POS	NEG
Beaver B_Berlin_WT63	POS	POS	POS	NEG	POS	POS	POS	POS	NEG	NEG	NEG	NEG	NEG	NEG	NEG	NEG	NEG	NEG	POS	NEG
Beaver B_Berlin_WT64	POS	POS	POS	NEG	POS	POS	POS	POS	NEG	NEG	NEG	NEG	NEG	NEG		NEG	NEG	NEG		
Beaver D_Berlin_WT66	POS	POS	POS	NEG	POS	POS	POS	POS	NEG	NEG	NEG	NEG	NEG	NEG		NEG	NEG	NEG		
Beaver D_Berlin_WT67A	POS	POS	POS	NEG	POS	POS	POS	POS	NEG	NEG	NEG	NEG	NEG	NEG		NEG	NEG	NEG		
Beaver D_Berlin_WT67B	POS	POS	POS	NEG	POS	POS	POS	POS	NEG	NEG	NEG	NEG	NEG	NEG	NEG	NEG	NEG	NEG	POS	NEG
Beaver D_Berlin_WT68	POS	POS	POS	NEG	POS	POS	POS	POS	NEG	NEG	NEG	NEG	NEG	NEG	NEG	NEG	NEG	NEG	POS	NEG
Beaver D_Berlin_WT69	POS	POS	POS	NEG	POS	POS	POS	POS	NEG	NEG	NEG	NEG	NEG	NEG		NEG	NEG	NEG		
Beaver F_Berlin_WT71	POS	POS	POS	NEG	POS	POS	POS	POS	NEG	NEG	NEG	NEG	NEG	NEG		NEG	NEG	NEG		
Beaver E_Berlin_WT70	POS	POS	POS	NEG	POS	POS	POS	POS	NEG	NEG	NEG	NEG	NEG	NEG		NEG	NEG	NEG		
Beaver G_Berlin_WT110	POS	POS	POS	NEG	POS	NEG	POS	POS	NEG	NEG	NEG	NEG	NEG	NEG		NEG	NEG	NEG		
Beaver G_Berlin_WT111	POS	POS	POS	NEG	POS	NEG	POS	POS	NEG	NEG	NEG	NEG	NEG	NEG		NEG	NEG	NEG		

STRAIN / ISOLATE	VIRULENCE : PROTEASES										VIRULENCE : STAPHYLOCOCCAL SUPERANTIGEN/ENTEROTOXIN-LIKE GENES (SET/SSL)											
	aur			splA	splB	splE	sspA	sspB	sspP		setC / selX	ssl01						ssl02		ssl03		
	aur (cons)	aur (Other than MRSA252)	aur (MRSA252)						sspP (cons)	sspP (other than ST93)		ssl01/set6 (COL)	ssl01/set6 (Mu50+ N315)	ssl01/set6 (MW2+ MSSA476)	ssl01/set6 (MRSA252)	ssl01/set6 (RF122)	ssl01/set6 (other alleles)	ssl02/set7	ssl02/set7 (MRSA252)	ssl03/set8_probe 1	ssl03/set8_probe 2	ssl03/set8 (MRSA252, SAR0424)
	aureolysin			serin- protease A	serin- protease B	serin- protease E	glutamylendopeptidase	Staphopain B, protease	Staphopain A (Staphylopain A), protease		Staphyl. exotoxin-like protein	Staphylococcal superantigen-like protein 1						Staphylococcal superantigen-like protein 2		Staphylococcal superantigen-like protein 3		
**>CC8-MSSA**																						
Strain NCTC8325: in silico predicted hybridisation pattern	POS	POS	NEG	POS	POS	POS	POS	POS	POS	POS	POS	POS	AMB	NEG	NEG	NEG	NEG	POS	AMB	POS	POS	NEG
Strain RN4220:VC40: in silico predicted hybridisation pattern	POS	POS	NEG	POS	POS	POS	POS	POS	POS	POS	POS	POS	AMB	NEG	NEG	NEG	NEG	POS	AMB	POS	POS	NEG
Strain Newman: in silico predicted hybridisation pattern	POS	POS	NEG	POS	POS	POS	POS	POS	POS	POS	POS	POS	AMB	NEG	NEG	NEG	NEG	POS	AMB	POS	POS	NEG
Beaver I_Austria_B2	POS	POS	NEG	POS	POS	POS	POS	POS	POS	POS	POS	POS	AMB	NEG	NEG	NEG	NEG	POS	NEG	POS	POS	NEG
Beaver J_Austria_B3	POS	POS	NEG	POS	POS	POS	POS	POS	POS	POS	POS	POS	AMB	NEG	NEG	NEG	NEG	POS	NEG	POS	POS	NEG
**>CC12-MSSA**																						
Strain KLT6: in silico predicted hybridisation pattern	POS	POS	NEG	POS	POS	POS	POS	POS	POS	POS	POS	NEG	NEG	NEG	NEG	NEG	POS	POS	AMB	POS	POS	NEG
Strain 21266: in silico predicted hybridisation pattern	POS	POS	NEG	POS	POS	POS	POS	POS	POS	POS	POS	NEG	NEG	NEG	NEG	NEG	POS	POS	AMB	POS	POS	NEG
NP66 chromosome: in silico predicted hybridisation pattern	POS	POS	NEG	POS	POS	POS	POS	POS	POS	POS	POS	NEG	NEG	NEG	NEG	NEG	POS	POS	NEG	POS	POS	NEG
Beaver K_Austria_B4	POS	POS	NEG	POS	POS	POS	POS	POS	POS	POS	POS	NEG	NEG	NEG	NEG	NEG	POS	POS	NEG	POS	POS	NEG
**>CC49-MSSA**																						
Strain 21262: in silico predicted hybridisation pattern	POS	POS	NEG	POS	POS	POS	AMB	POS	POS	POS	POS	NEG	NEG	NEG	NEG	NEG	POS	POS	NEG	POS	POS	NEG
Strain Tager 104: in silico predicted hybridisation pattern	POS	POS	NEG	POS	POS	POS	AMB	POS	POS	POS	POS	NEG	NEG	NEG	NEG	NEG	POS	POS	NEG	POS	POS	NEG
**>CC49-MSSA (lukF-P83/lukM+)**																						
Strain 22_M61_07_10: in silico predicted hybridisation pattern	POS	POS	NEG	POS	POS	POS	AMB	POS	POS	POS	POS	NEG	NEG	NEG	NEG	NEG	POS	POS	NEG	POS	POS	NEG
Strain 22_M48_10_10: in silico predicted hybridisation pattern	POS	POS	NEG	NEG	POS	POS	AMB	POS	POS	POS	POS	NEG	NEG	NEG	NEG	NEG	POS	POS	NEG	POS	POS	NEG
Strain 22_M47_10_10: in silico predicted hybridisation pattern	POS	POS	NEG	NEG	POS	POS	AMB	POS	POS	POS	POS	NEG	NEG	NEG	NEG	NEG	POS	POS	NEG	POS	POS	NEG
**>CC49-MSSA (lukF-PV+/lukS-PV?)**																						
Beaver C_Bavaria_WT65: in silico predicted hybridisation pattern	POS	POS	NEG	POS	POS	POS	AMB	POS	POS	POS	POS	NEG	NEG	NEG	NEG	NEG	POS	POS	NEG	POS	POS	NEG
Beaver C_Bavaria_WT65	POS	POS	NEG	POS	POS	POS	POS	POS	POS	POS	POS	NEG	NEG	NEG	NEG	NEG	POS	POS	NEG	POS	POS	NEG
**>CC398-MSSA**																						
Strain 21331: in silico predicted hybridisation pattern	POS	NEG	POS	NEG	NEG	NEG	POS	POS	POS	POS	POS	NEG	POS	NEG	NEG	NEG	NEG	AMB	POS	NEG	NEG	NEG
Strain 71193: in silico predicted hybridisation pattern	POS	NEG	POS	NEG	NEG	NEG	POS	POS	POS	POS	POS	NEG	POS	NEG	NEG	NEG	NEG	AMB	POS	NEG	NEG	NEG
Strain S123: in silico predicted hybridisation pattern	POS	NEG	POS	NEG	NEG	NEG	POS	POS	POS	POS	POS	NEG	POS	NEG	NEG	NEG	NEG	AMB	POS	NEG	NEG	NEG
Beaver H_Austria_B1	POS	NEG	POS	NEG	NEG	NEG	POS	POS	POS	POS	POS	NEG	POS	NEG	NEG	NEG	NEG	NEG	POS	NEG	NEG	NEG
**>CC1956-MSSA**																						
Strain C6589: in silico predicted hybridisation pattern	POS	POS	NEG	POS	POS	NEG	POS	POS	POS	POS	POS	NEG	NEG	NEG	NEG	POS	NEG	POS	AMB	AMB	POS	NEG
Strain C6591: in silico predicted hybridisation pattern	POS	POS	NEG	POS	POS	NEG	POS	POS	POS	POS	POS	NEG	NEG	NEG	NEG	POS	NEG	POS	AMB	AMB	POS	NEG
Strain C6597: in silico predicted hybridisation pattern	POS	POS	NEG	POS	POS	NEG	POS	POS	POS	POS	POS	NEG	NEG	NEG	NEG	POS	NEG	POS	AMB	AMB	POS	NEG
Strain C6598: in silico predicted hybridisation pattern	POS	POS	NEG	POS	POS	NEG	POS	POS	POS	POS	POS	NEG	NEG	NEG	NEG	POS	NEG	POS	AMB	AMB	POS	NEG
Strain C6601: in silico predicted hybridisation pattern	POS	POS	NEG	POS	POS	NEG	POS	POS	POS	POS	POS	NEG	NEG	NEG	NEG	POS	NEG	POS	AMB	AMB	POS	NEG
**>CC1956-MSSA (lukF-PV+/lukS-PV?)**																						
Beaver A_Berlin_WT19: in silico predicted hybridisation pattern	POS	POS	NEG	POS	POS	POS	POS	POS	POS	POS	POS	NEG	NEG	NEG	NEG	POS	NEG	POS	NEG	AMB	POS	NEG
Beaver A_Berlin_WT19	POS	POS	NEG	POS	POS	POS	POS	POS	POS	POS	POS	NEG	NEG	NEG	NEG	POS	NEG	POS	NEG	POS	POS	NEG
Beaver B_Berlin_WT63	POS	POS	NEG	POS	POS	POS	POS	POS	POS	POS	POS	NEG	NEG	NEG	NEG	POS	NEG	POS	AMB	POS	POS	NEG
Beaver B_Berlin_WT64	POS	POS	NEG	POS	POS	POS	POS	POS	POS	POS	POS	NEG	NEG	NEG	NEG	POS	NEG	POS	AMB	POS	POS	NEG
Beaver D_Berlin_WT66	POS	POS	NEG	POS	POS	POS	POS	POS	POS	POS	POS	NEG	NEG	NEG	NEG	POS	NEG	POS	AMB	POS	POS	NEG
Beaver D_Berlin_WT67A	POS	POS	NEG	POS	POS	POS	POS	POS	POS	POS	POS	NEG	NEG	NEG	NEG	POS	NEG	POS	AMB	POS	POS	NEG
Beaver D_Berlin_WT67B	POS	POS	NEG	POS	POS	POS	POS	POS	POS	POS	POS	NEG	NEG	NEG	NEG	POS	NEG	POS	AMB	POS	POS	NEG
Beaver D_Berlin_WT68	POS	POS	NEG	POS	POS	POS	POS	POS	POS	POS	POS	NEG	NEG	NEG	NEG	POS	NEG	POS	AMB	POS	POS	NEG
Beaver D_Berlin_WT69	POS	POS	NEG	POS	POS	POS	POS	POS	POS	POS	POS	NEG	NEG	NEG	NEG	POS	NEG	POS	AMB	POS	POS	NEG
Beaver F_Berlin_WT71	POS	POS	NEG	POS	POS	POS	POS	POS	POS	POS	POS	NEG	NEG	NEG	NEG	POS	NEG	POS	AMB	POS	POS	NEG
Beaver E_Berlin_WT70	POS	POS	NEG	POS	POS	POS	POS	POS	POS	POS	POS	NEG	NEG	NEG	NEG	POS	NEG	POS	AMB	POS	POS	NEG
Beaver G_Berlin_WT110	POS	POS	NEG	POS	POS	POS	POS	POS	POS	POS	POS	NEG	NEG	NEG	NEG	POS	NEG	POS	NEG	NEG	POS	NEG
Beaver G_Berlin_WT111	POS	POS	NEG	POS	POS	POS	POS	POS	POS	POS	POS	NEG	NEG	NEG	NEG	POS	NEG	POS	NEG	NEG	POS	NEG

STRAIN / ISOLATE	VIRULENCE : STAPHYLOCOCCAL SUPERANTIGEN/ENTEROTOXIN-LIKE GENES (SET/SSL)																					
	ssl04		ssl05				ssl06		ssl07			ssl08	ssl09			ssl10			ssl11			
	ssl04/set9	ssl04/set9 (MRSA252, SAR0425)	ssl05/set3_probe 1	ssl05/set3 (RF122, probe-611)	ssl05/set3_probe 2 (612)	ssl05/set3 (MRSA252)	ssl06/set21	ssl06 (NCTC8325+ MW2)	ssl07/set1	ssl07/set1 (MRSA252)	ssl07/set1 (AF188836)	ssl08	ssl09/set5_probe 1	ssl09/set5_probe 2	ssl09/set5 (MRSA252)	ssl10/set4	ssl10 (RF122)	ssl10/set4 (MRSA252)	ssl11/set2 (COL)	ssl11/set2 (Mu50+ N315)	ssl11/set2 (MW2+ MSSA476)	ssl11/set2 (MRSA252)
	Staphylococcal superantigen-like protein 4		Staphylococcal superantigen-like protein 5				Staphylococcal superantigen-like protein 6		Staphylococcal superantigen-like protein 7			Staphylococcal superantigen-like protein 8	Staphylococcal superantigen-like protein 9			Staphylococcal superantigen-like protein 10			Staphylococcal superantigene-like protein 11			
**>CC8-MSSA**																						
Strain NCTC8325: in silico predicted hybridisation pattern	POS	NEG	POS	NEG	POS	NEG	POS	POS	POS	NEG	NEG	POS	POS	POS	NEG	POS	NEG	NEG	POS	NEG	NEG	NEG
Strain RN4220:VC40: in silico predicted hybridisation pattern	POS	NEG	POS	NEG	POS	NEG	POS	POS	POS	NEG	NEG	POS	POS	POS	NEG	POS	NEG	NEG	POS	NEG	NEG	NEG
Strain Newman: in silico predicted hybridisation pattern	POS	NEG	POS	NEG	POS	NEG	POS	POS	POS	NEG	NEG	POS	POS	POS	NEG	POS	NEG	NEG	POS	NEG	NEG	NEG
Beaver I_Austria_B2	POS	NEG	POS	NEG	POS	NEG	POS	POS	POS	NEG	NEG	POS	POS	POS	NEG	POS	NEG	NEG	POS	NEG	NEG	POS
Beaver J_Austria_B3	POS	NEG	POS	NEG	POS	NEG	POS	POS	POS	NEG	NEG	POS	POS	POS	NEG	POS	NEG	NEG	POS	NEG	NEG	POS
**>CC12-MSSA**																						
Strain KLT6: in silico predicted hybridisation pattern	POS	NEG	POS	NEG	POS	NEG	NEG	NEG	POS	NEG	NEG	POS	POS	POS	NEG	POS	NEG	NEG	POS	NEG	NEG	NEG
Strain 21266: in silico predicted hybridisation pattern	POS	NEG	POS	NEG	POS	NEG	NEG	NEG	POS	NEG	NEG	POS	POS	POS	NEG	POS	NEG	NEG	POS	NEG	NEG	NEG
NP66 chromosome: in silico predicted hybridisation pattern	POS	NEG	POS	NEG	POS	NEG	NEG	NEG	POS	NEG	NEG	POS	POS	POS	NEG	POS	NEG	NEG	POS	NEG	NEG	NEG
Beaver K_Austria_B4	POS	NEG	POS	NEG	POS	NEG	NEG	NEG	POS	NEG	NEG	POS	POS	POS	NEG	POS	NEG	NEG	POS	NEG	NEG	AMB
**>CC49-MSSA**																						
Strain 21262: in silico predicted hybridisation pattern	POS	NEG	NEG	POS	NEG	NEG	POS	POS	POS	NEG	NEG	POS	POS	POS	NEG	AMB	AMB	NEG	NEG	NEG	NEG	NEG
Strain Tager 104: in silico predicted hybridisation pattern	POS	NEG	NEG	POS	NEG	NEG	POS	POS	POS	NEG	NEG	POS	POS	POS	NEG	AMB	AMB	NEG	NEG	NEG	NEG	NEG
**>CC49-MSSA (lukF-P83/lukM+)**																						
Strain 22_M61_07_10: in silico predicted hybridisation pattern	POS	NEG	NEG	POS	NEG	NEG	POS	POS	POS	NEG	NEG	POS	POS	POS	NEG	AMB	AMB	NEG	NEG	NEG	NEG	NEG
Strain 22_M48_10_10: in silico predicted hybridisation pattern	POS	NEG	NEG	POS	NEG	NEG	POS	POS	POS	NEG	NEG	POS	POS	POS	NEG	AMB	AMB	NEG	NEG	NEG	NEG	NEG
Strain 22_M47_10_10: in silico predicted hybridisation pattern	POS	NEG	NEG	POS	NEG	NEG	POS	POS	POS	NEG	NEG	POS	POS	POS	NEG	AMB	AMB	NEG	NEG	NEG	NEG	NEG
**>CC49-MSSA (lukF-PV+/lukS-PV?)**																						
Beaver C_Bavaria_WT65: in silico predicted hybridisation pattern	POS	NEG	NEG	POS	NEG	NEG	POS	POS	POS	NEG	NEG	POS	POS	POS	NEG	AMB	AMB	NEG	NEG	NEG	NEG	NEG
Beaver C_Bavaria_WT65	POS	AMB	NEG	POS	AMB	NEG	POS	POS	POS	AMB	AMB	POS	POS	POS	NEG	POS	AMB	AMB	NEG	NEG	NEG	NEG
**>CC398-MSSA**																						
Strain 21331: in silico predicted hybridisation pattern	NEG	POS	NEG	NEG	NEG	POS	NEG	NEG	NEG	NEG	POS	NEG	NEG	NEG	POS	NEG	NEG	POS	NEG	NEG	NEG	POS
Strain 71193: in silico predicted hybridisation pattern	NEG	POS	NEG	NEG	NEG	POS	NEG	NEG	NEG	NEG	POS	NEG	NEG	NEG	POS	NEG	NEG	POS	NEG	NEG	NEG	POS
Strain S123: in silico predicted hybridisation pattern	NEG	POS	NEG	NEG	NEG	POS	NEG	NEG	NEG	NEG	POS	NEG	NEG	NEG	POS	NEG	NEG	POS	NEG	NEG	NEG	POS
Beaver H_Austria_B1	NEG	POS	NEG	NEG	NEG	POS	NEG	NEG	AMB	AMB	POS	NEG	NEG	NEG	POS	AMB	NEG	POS	NEG	NEG	NEG	POS
**>CC1956-MSSA**																						
Strain C6589: in silico predicted hybridisation pattern	POS	NEG	NEG	POS	NEG	NEG	NEG	NEG	POS	NEG	NEG	POS	POS	POS	NEG	NEG	POS	NEG	NEG	NEG	NEG	NEG
Strain C6591: in silico predicted hybridisation pattern	POS	NEG	NEG	POS	NEG	NEG	NEG	NEG	POS	NEG	NEG	POS	POS	POS	NEG	NEG	POS	NEG	NEG	NEG	NEG	NEG
Strain C6597: in silico predicted hybridisation pattern	POS	NEG	NEG	POS	NEG	NEG	NEG	NEG	POS	NEG	NEG	POS	POS	POS	NEG	NEG	POS	NEG	NEG	NEG	NEG	NEG
Strain C6598: in silico predicted hybridisation pattern	POS	NEG	NEG	POS	NEG	NEG	NEG	NEG	POS	NEG	NEG	POS	POS	POS	NEG	NEG	POS	NEG	NEG	NEG	NEG	NEG
Strain C6601: in silico predicted hybridisation pattern	POS	NEG	NEG	POS	NEG	NEG	NEG	NEG	POS	NEG	NEG	POS	POS	POS	NEG	NEG	POS	NEG	NEG	NEG	NEG	NEG
**>CC1956-MSSA (lukF-PV+/lukS-PV?)**																						
Beaver A_Berlin_WT19: in silico predicted hybridisation pattern	POS	NEG	NEG	POS	NEG	NEG	NEG	NEG	POS	NEG	NEG	POS	POS	POS	NEG	NEG	POS	NEG	NEG	NEG	NEG	NEG
Beaver A_Berlin_WT19	POS	NEG	NEG	POS	AMB	NEG	NEG	NEG	POS	AMB	NEG	POS	POS	POS	NEG	POS	POS	NEG	NEG	NEG	NEG	NEG
Beaver B_Berlin_WT63	POS	AMB	NEG	POS	AMB	NEG	NEG	NEG	POS	AMB	AMB	POS	POS	POS	NEG	POS	AMB	NEG	NEG	NEG	NEG	NEG
Beaver B_Berlin_WT64	POS	NEG	NEG	POS	AMB	NEG	NEG	NEG	POS	AMB	AMB	POS	POS	POS	NEG	AMB	POS	NEG	NEG	NEG	NEG	NEG
Beaver D_Berlin_WT66	POS	AMB	NEG	POS	AMB	NEG	NEG	NEG	POS	AMB	AMB	POS	POS	POS	NEG	POS	AMB	NEG	NEG	NEG	NEG	NEG
Beaver D_Berlin_WT67A	POS	AMB	NEG	AMB	POS	NEG	NEG	NEG	POS	AMB	AMB	POS	POS	POS	NEG	POS	AMB	NEG	NEG	NEG	NEG	NEG
Beaver D_Berlin_WT67B	POS	NEG	NEG	POS	AMB	NEG	NEG	NEG	POS	AMB	NEG	POS	POS	POS	NEG	AMB	POS	NEG	NEG	NEG	NEG	NEG
Beaver D_Berlin_WT68	POS	NEG	NEG	POS	AMB	NEG	NEG	NEG	POS	AMB	AMB	POS	POS	POS	NEG	AMB	POS	NEG	NEG	NEG	NEG	NEG
Beaver D_Berlin_WT69	POS	NEG	NEG	POS	AMB	NEG	NEG	NEG	POS	AMB	AMB	POS	POS	POS	NEG	AMB	POS	NEG	NEG	NEG	NEG	NEG
Beaver F_Berlin_WT71	POS	NEG	NEG	POS	AMB	NEG	NEG	NEG	POS	AMB	AMB	POS	POS	POS	NEG	AMB	POS	NEG	NEG	NEG	NEG	NEG
Beaver E_Berlin_WT70	POS	NEG	NEG	POS	AMB	NEG	NEG	NEG	POS	AMB	AMB	POS	POS	POS	NEG	AMB	POS	NEG	NEG	NEG	NEG	NEG
Beaver G_Berlin_WT110	POS	NEG	NEG	POS	NEG	NEG	NEG	NEG	POS	NEG	NEG	POS	POS	POS	NEG	AMB	POS	NEG	NEG	NEG	NEG	NEG
Beaver G_Berlin_WT111	POS	NEG	NEG	POS	NEG	NEG	NEG	NEG	POS	NEG	NEG	POS	POS	POS	NEG	AMB	POS	NEG	NEG	NEG	NEG	NEG

STRAIN / ISOLATE	VIRULENCE : SET/SSL GENES					CAPSULE- AND BIOFILM-ASSOCIATED GENES																
	setB3		setB2		setB1	Capsule type 1				Capsule type 5				Capsule type 8					icaA	icaC	icaD	bap
	setB3	setB3 (MRSA252)	setB2	setB2 (MRSA252)		cap 1	capH1	capJ1	capK1	cap 5	capH5	capJ5	capK5	cap 8	capH8	capI8	capJ8	capK8				
	Staphylococcal exotoxin-like protein, second locus					Capsule type 1	capsular poly-saccharide synthesis enzyme	O-antigen polymerase	capsular poly-saccharide biosyn-thesis protein	Capsule type 5	capsular poly-saccharide synthesis enzyme	O-antigen polymerase	capsular poly-saccharide biosyn-thesis protein	Capsule type 8	capsular poly-saccharide synthesis enzyme	capsular poly-saccharide biosyn-thesis protein	O-antigen poly-merase	capsular poly-saccharide biosyn-thesis protein	intercellular adhesion protein A	intercellular adhesion protein C	biofilm PIA synthesis protein D	Surface protein involved in biofilm formation
**>CC8-MSSA**																						
Strain NCTC8325: in silico predicted hybridisation pattern	POS	NEG	POS	NEG	POS	NEG	NEG	NEG	NEG	POS	POS	POS	POS	NEG	NEG	NEG	NEG	NEG	POS	POS	POS	NEG
Strain RN4220:VC40: in silico predicted hybridisation pattern	POS	NEG	POS	NEG	POS	NEG	NEG	NEG	NEG	POS	POS	POS	POS	NEG	NEG	NEG	NEG	NEG	POS	POS	POS	NEG
Strain Newman: in silico predicted hybridisation pattern	POS	NEG	POS	NEG	POS	NEG	NEG	NEG	NEG	POS	POS	POS	POS	NEG	NEG	NEG	NEG	NEG	POS	POS	POS	NEG
Beaver I_Austria_B2	POS	NEG	POS	NEG	POS	NEG	NEG	NEG	NEG	POS	POS	POS	POS	NEG	NEG	NEG	NEG	NEG	POS	POS	POS	NEG
Beaver J_Austria_B3	POS	NEG	POS	NEG	POS	NEG	NEG	NEG	NEG	POS	POS	POS	POS	NEG	NEG	NEG	NEG	NEG	POS	POS	POS	NEG
**>CC12-MSSA**																						
Strain KLT6: in silico predicted hybridisation pattern	POS	NEG	POS	NEG	POS	NEG	NEG	NEG	NEG	NEG	NEG	NEG	NEG	POS	POS	POS	POS	POS	POS	POS	POS	NEG
Strain 21266: in silico predicted hybridisation pattern	POS	NEG	POS	NEG	POS	NEG	NEG	NEG	NEG	NEG	NEG	NEG	NEG	POS	POS	POS	POS	POS	POS	POS	POS	NEG
NP66 chromosome: in silico predicted hybridisation pattern	POS	NEG	POS	NEG	POS	NEG	NEG	NEG	NEG	NEG	NEG	NEG	NEG	POS	POS	POS	POS	POS	POS	POS	POS	NEG
Beaver K_Austria_B4	POS	NEG	POS	NEG	POS	NEG	NEG	NEG	NEG	NEG	NEG	NEG	NEG	POS	POS	POS	POS	POS	POS	POS	POS	NEG
**>CC49-MSSA**																						
Strain 21262: in silico predicted hybridisation pattern	POS	NEG	POS	NEG	POS	NEG	NEG	NEG	NEG	POS	POS	POS	POS	NEG	NEG	NEG	NEG	NEG	POS	POS	POS	NEG
Strain Tager 104: in silico predicted hybridisation pattern	POS	NEG	POS	NEG	POS	NEG	NEG	NEG	NEG	POS	POS	POS	POS	NEG	NEG	NEG	NEG	NEG	POS	POS	POS	NEG
**>CC49-MSSA (lukF-P83/lukM+)**																						
Strain 22_M61_07_10: in silico predicted hybridisation pattern	POS	NEG	POS	NEG	POS	NEG	NEG	NEG	NEG	POS	POS	POS	POS	NEG	NEG	NEG	NEG	NEG	POS	POS	POS	NEG
Strain 22_M48_10_10: in silico predicted hybridisation pattern	POS	NEG	POS	NEG	POS	NEG	NEG	NEG	NEG	POS	POS	POS	POS	NEG	NEG	NEG	NEG	NEG	POS	POS	POS	NEG
Strain 22_M47_10_10: in silico predicted hybridisation pattern	POS	NEG	POS	NEG	POS	NEG	NEG	NEG	NEG	POS	POS	POS	POS	NEG	NEG	NEG	NEG	NEG	POS	POS	POS	NEG
**>CC49-MSSA (lukF-PV+/lukS-PV?)**																						
Beaver C_Bavaria_WT65: in silico predicted hybridisation pattern	POS	NEG	POS	NEG	POS	NEG	NEG	NEG	NEG	POS	POS	POS	POS	NEG	NEG	NEG	NEG	NEG	POS	POS	POS	NEG
Beaver C_Bavaria_WT65	POS	NEG	POS	NEG	POS	NEG	NEG	NEG	NEG	POS	POS	POS	POS	NEG	NEG	NEG	NEG	NEG	POS	POS	POS	NEG
**>CC398-MSSA**																						
Strain 21331: in silico predicted hybridisation pattern	NEG	POS	NEG	POS	AMB	NEG	NEG	NEG	NEG	POS	POS	POS	POS	NEG	NEG	NEG	NEG	NEG	POS	POS	POS	NEG
Strain 71193: in silico predicted hybridisation pattern	NEG	POS	NEG	POS	AMB	NEG	NEG	NEG	NEG	POS	POS	POS	POS	NEG	NEG	NEG	NEG	NEG	POS	POS	POS	NEG
Strain S123: in silico predicted hybridisation pattern	NEG	POS	NEG	POS	AMB	NEG	NEG	NEG	NEG	POS	POS	POS	POS	NEG	NEG	NEG	NEG	NEG	POS	POS	POS	NEG
Beaver H_Austria_B1	NEG	POS	AMB	POS	POS	NEG	NEG	NEG	NEG	POS	POS	POS	POS	NEG	NEG	NEG	NEG	NEG	POS	POS	POS	NEG
**>CC1956-MSSA**																						
Strain C6589: in silico predicted hybridisation pattern	POS	NEG	POS	NEG	POS	NEG	NEG	NEG	NEG	POS	POS	POS	POS	NEG	NEG	NEG	NEG	NEG	POS	POS	POS	NEG
Strain C6591: in silico predicted hybridisation pattern	POS	NEG	POS	NEG	POS	NEG	NEG	NEG	NEG	POS	POS	POS	POS	NEG	NEG	NEG	NEG	NEG	POS	POS	POS	NEG
Strain C6597: in silico predicted hybridisation pattern	POS	NEG	NEG	NEG	POS	NEG	NEG	NEG	NEG	POS	POS	POS	POS	NEG	NEG	NEG	NEG	NEG	POS	POS	POS	NEG
Strain C6598: in silico predicted hybridisation pattern	POS	NEG	POS	NEG	POS	NEG	NEG	NEG	NEG	POS	POS	POS	POS	NEG	NEG	NEG	NEG	NEG	POS	POS	POS	NEG
Strain C6601: in silico predicted hybridisation pattern	POS	NEG	POS	NEG	POS	NEG	NEG	NEG	NEG	POS	POS	POS	POS	NEG	NEG	NEG	NEG	NEG	POS	POS	POS	NEG
**>CC1956-MSSA (lukF-PV+/lukS-PV?)**																						
Beaver A_Berlin_WT19: in silico predicted hybridisation pattern	POS	NEG	POS	NEG	POS	NEG	NEG	NEG	NEG	POS	POS	POS	POS	NEG	NEG	NEG	NEG	NEG	POS	POS	POS	NEG
Beaver A_Berlin_WT19	POS	NEG	POS	NEG	POS	NEG	NEG	NEG	NEG	POS	POS	POS	POS	NEG	NEG	NEG	NEG	NEG	POS	POS	POS	NEG
Beaver B_Berlin_WT63	POS	NEG	POS	NEG	POS	NEG	NEG	NEG	NEG	POS	POS	POS	POS	NEG	NEG	NEG	NEG	NEG	POS	POS	POS	NEG
Beaver B_Berlin_WT64	POS	NEG	POS	NEG	POS	NEG	NEG	NEG	NEG	POS	POS	POS	POS	NEG	NEG	NEG	NEG	NEG	POS	POS	POS	NEG
Beaver D_Berlin_WT66	POS	NEG	POS	NEG	POS	NEG	NEG	NEG	NEG	POS	POS	POS	POS	NEG	NEG	NEG	NEG	NEG	POS	POS	POS	NEG
Beaver D_Berlin_WT67A	POS	NEG	POS	NEG	POS	NEG	NEG	NEG	NEG	POS	POS	POS	POS	NEG	NEG	NEG	NEG	NEG	POS	POS	POS	NEG
Beaver D_Berlin_WT67B	POS	NEG	POS	NEG	POS	NEG	NEG	NEG	NEG	POS	POS	POS	POS	NEG	NEG	NEG	NEG	NEG	POS	POS	POS	NEG
Beaver D_Berlin_WT68	POS	NEG	POS	NEG	POS	NEG	NEG	NEG	NEG	POS	POS	POS	POS	NEG	NEG	NEG	NEG	NEG	POS	POS	POS	NEG
Beaver D_Berlin_WT69	POS	NEG	POS	NEG	POS	NEG	NEG	NEG	NEG	POS	POS	POS	POS	NEG	NEG	NEG	NEG	NEG	POS	POS	POS	NEG
Beaver F_Berlin_WT71	POS	NEG	POS	NEG	POS	NEG	NEG	NEG	NEG	POS	POS	POS	POS	NEG	NEG	NEG	NEG	NEG	POS	POS	POS	NEG
Beaver E_Berlin_WT70	POS	NEG	POS	NEG	POS	NEG	NEG	NEG	NEG	POS	POS	POS	POS	NEG	NEG	NEG	NEG	NEG	POS	POS	POS	NEG
Beaver G_Berlin_WT110	POS	NEG	POS	NEG	POS	NEG	NEG	NEG	NEG	POS	POS	POS	POS	NEG	NEG	NEG	NEG	NEG	POS	POS	POS	NEG
Beaver G_Berlin_WT111	POS	NEG	POS	NEG	POS	NEG	NEG	NEG	NEG	POS	POS	POS	POS	NEG	NEG	NEG	NEG	NEG	POS	POS	POS	NEG

STRAIN / ISOLATE	bbp	bbp (cons)	bbp (COL+MW2)	bbp (MRSA252)	bbp (Mu50)	bbp (RF122)	bbp (ST45)	clfA	clfA (cons)	clfA (COL+RF122)	clfA (MRSA252)	clfA (Mu50+MW2)	clfB	clfB (cons)	clfB (COL+Mu50)	clfB (MW2)	clfB (RF122)	cna
	ADHAESION FACTORS / GENES ENCODING MICROBIAL SURFACE COMPONENTS RECOGNIZING ADHESIVE MATRIX MOLECULES (MSCRAMM GENES)																	
	bbp							clfA					clfB					cna
	Bone sialoprotein-binding protein							Clumping factor A					Clumping factor B					Collagen-binding adhesin
**>CC8-MSSA**																		
Strain NCTC8325: in silico predicted hybridisation pattern	NEG	NEG	NEG	NEG	NEG	NEG	NEG	POS	POS	POS	NEG	AMB	POS	POS	POS	NEG	NEG	NEG
Strain RN4220:VC40: in silico predicted hybridisation pattern	NEG	NEG	NEG	NEG	NEG	NEG	NEG	POS	POS	POS	NEG	AMB	POS	POS	POS	NEG	NEG	NEG
Strain Newman: in silico predicted hybridisation pattern	POS	POS	POS	NEG	NEG	NEG	NEG	POS	POS	POS	NEG	AMB	POS	POS	POS	NEG	NEG	NEG
Beaver I_Austria_B2	AMB	NEG	NEG	NEG	NEG	AMB	NEG	POS	POS	POS	AMB	AMB	POS	POS	POS	NEG	NEG	NEG
Beaver J_Austria_B3	POS	POS	POS	NEG	NEG	NEG	NEG	POS	POS	POS	NEG	AMB	POS	POS	POS	NEG	NEG	NEG
**>CC12-MSSA**																		
Strain KLT6: in silico predicted hybridisation pattern	NEG	NEG	NEG	NEG	NEG	NEG	NEG	POS	POS	POS	NEG	AMB	POS	POS	NEG	NEG	NEG	POS
Strain 21266: in silico predicted hybridisation pattern	NEG	NEG	NEG	NEG	NEG	NEG	NEG	POS	POS	POS	NEG	AMB	POS	POS	NEG	NEG	NEG	POS
NP66 chromosome: in silico predicted hybridisation pattern	POS	POS	NEG	NEG	POS	NEG	NEG	POS	POS	POS	NEG	NEG	POS	POS	NEG	NEG	NEG	POS
Beaver K_Austria_B4	POS	POS	NEG	NEG	POS	NEG	NEG	POS	POS	POS	NEG	AMB	POS	POS	NEG	NEG	NEG	POS
**>CC49-MSSA**																		
Strain 21262: in silico predicted hybridisation pattern	POS	POS	NEG	NEG	NEG	NEG	NEG	POS	POS	NEG	NEG	POS	POS	POS	NEG	POS	AMB	NEG
Strain Tager 104: in silico predicted hybridisation pattern	POS	POS	NEG	NEG	NEG	NEG	NEG	POS	POS	NEG	NEG	POS	POS	POS	NEG	POS	AMB	NEG
**>CC49-MSSA (lukF-P83/lukM+)**																		
Strain 22_M61_07_10: in silico predicted hybridisation pattern	POS	POS	NEG	NEG	NEG	NEG	NEG	POS	POS	NEG	NEG	POS	POS	POS	NEG	POS	AMB	NEG
Strain 22_M48_10_10: in silico predicted hybridisation pattern	POS	POS	NEG	NEG	NEG	NEG	NEG	POS	POS	NEG	NEG	POS	POS	POS	NEG	POS	AMB	NEG
Strain 22_M47_10_10: in silico predicted hybridisation pattern	POS	POS	NEG	NEG	NEG	NEG	NEG	POS	POS	NEG	NEG	POS	POS	POS	NEG	POS	AMB	NEG
**>CC49-MSSA (lukF-PV+/lukS-PV?)**																		
Beaver C_Bavaria_WT65: in silico predicted hybridisation pattern	POS	POS	NEG	NEG	NEG	NEG	NEG	POS	POS	NEG	NEG	POS	POS	POS	NEG	POS	NEG	NEG
Beaver C_Bavaria_WT65	POS	POS	AMB	NEG	POS	NEG	NEG	POS	POS	NEG	NEG	POS	POS	POS	NEG	POS	AMB	NEG
**>CC398-MSSA**																		
Strain 21331: in silico predicted hybridisation pattern	POS	POS	AMB	NEG	NEG	NEG	POS	POS	POS	POS	NEG	AMB	POS	POS	NEG	AMB	AMB	POS
Strain 71193: in silico predicted hybridisation pattern	POS	POS	AMB	NEG	NEG	NEG	POS	POS	POS	POS	NEG	AMB	POS	POS	NEG	AMB	AMB	POS
Strain S123: in silico predicted hybridisation pattern	POS	POS	AMB	NEG	NEG	NEG	POS	POS	POS	POS	NEG	AMB	POS	POS	NEG	AMB	AMB	POS
Beaver H_Austria_B1	POS	POS	NEG	NEG	NEG	NEG	POS	POS	POS	POS	AMB	AMB	POS	POS	NEG	AMB	POS	POS
**>CC1956-MSSA**																		
Strain C6589: in silico predicted hybridisation pattern	POS	POS	NEG	NEG	NEG	NEG	POS	POS	POS	NEG	NEG	POS	POS	POS	NEG	NEG	NEG	POS
Strain C6591: in silico predicted hybridisation pattern	POS	POS	NEG	NEG	NEG	NEG	POS	POS	POS	NEG	NEG	POS	POS	POS	NEG	NEG	NEG	POS
Strain C6597: in silico predicted hybridisation pattern	POS	POS	NEG	NEG	NEG	NEG	POS	POS	POS	NEG	NEG	POS	POS	POS	NEG	NEG	NEG	POS
Strain C6598: in silico predicted hybridisation pattern	POS	POS	NEG	NEG	NEG	NEG	POS	POS	POS	NEG	NEG	POS	POS	POS	NEG	NEG	NEG	POS
Strain C6601: in silico predicted hybridisation pattern	POS	POS	NEG	NEG	NEG	NEG	POS	POS	POS	NEG	NEG	POS	POS	POS	NEG	NEG	NEG	POS
**>CC1956-MSSA (lukF-PV+/lukS-PV?)**																		
Beaver A_Berlin_WT19: in silico predicted hybridisation pattern	POS	POS	NEG	NEG	NEG	NEG	POS	POS	POS	NEG	NEG	POS	POS	POS	NEG	NEG	NEG	POS
Beaver A_Berlin_WT19	POS	POS	NEG	NEG	NEG	NEG	AMB	POS	POS	NEG	NEG	POS	POS	POS	NEG	NEG	POS	POS
Beaver B_Berlin_WT63	POS	POS	NEG	NEG	NEG	NEG	POS	POS	POS	NEG	NEG	POS	POS	POS	NEG	NEG	POS	POS
Beaver B_Berlin_WT64	POS	POS	NEG	NEG	NEG	NEG	POS	POS	POS	NEG	NEG	POS	POS	POS	NEG	NEG	POS	POS
Beaver D_Berlin_WT66	POS	POS	AMB	NEG	NEG	NEG	POS	POS	POS	NEG	NEG	POS	POS	POS	NEG	NEG	POS	POS
Beaver D_Berlin_WT67A	POS	POS	AMB	NEG	NEG	NEG	POS	POS	POS	NEG	NEG	POS	POS	POS	NEG	NEG	POS	POS
Beaver D_Berlin_WT67B	POS	POS	NEG	NEG	NEG	NEG	POS	POS	POS	NEG	NEG	POS	POS	POS	NEG	NEG	NEG	POS
Beaver D_Berlin_WT68	POS	POS	NEG	NEG	NEG	NEG	POS	POS	POS	NEG	NEG	POS	POS	POS	NEG	NEG	POS	POS
Beaver D_Berlin_WT69	POS	POS	NEG	NEG	NEG	NEG	POS	POS	POS	NEG	NEG	POS	POS	POS	NEG	NEG	POS	POS
Beaver F_Berlin_WT71	POS	POS	NEG	NEG	NEG	NEG	POS	POS	POS	NEG	NEG	POS	POS	POS	NEG	NEG	POS	POS
Beaver E_Berlin_WT70	POS	POS	NEG	NEG	NEG	NEG	POS	POS	POS	NEG	NEG	POS	POS	POS	NEG	NEG	POS	POS
Beaver G_Berlin_WT110	POS	POS	NEG	NEG	NEG	NEG	NEG	POS	POS	NEG	NEG	POS	POS	POS	NEG	NEG	NEG	POS
Beaver G_Berlin_WT111	POS	POS	NEG	NEG	NEG	NEG	NEG	POS	POS	NEG	NEG	POS	POS	POS	NEG	NEG	NEG	POS

STRAIN / ISOLATE	ADHAESION FACTORS / GENES ENCODING MICROBIAL SURFACE COMPONENTS RECOGNIZING ADHESIVE MATRIX MOLECULES (MSCRAMM GENES)														
	ebh	ebpS					eno	efb		fnbA					
	ebh (cons)	ebpS	ebpS_probe 612	ebpS_probe 614	ebpS (01-1111)	ebpS (COL)		efb / fib	efb / fib (MRSA252)	fnbA	fnbA (cons)	fnbA (COL)	fnbA (MRSA252)	fnbA (Mu50+MW2)	fnbA (RF122)
	Cell wall associated fibronectin-binding protein	cell surface elastin binding protein					enolase	fibrinogen binding protein (19 kDa)		fibronectin-binding protein A					
**>CC8-MSSA**															
Strain NCTC8325: in silico predicted hybridisation pattern	POS	POS	POS	POS	NEG	POS	POS	POS	NEG	POS	POS	POS	NEG	NEG	NEG
Strain RN4220:VC40: in silico predicted hybridisation pattern	POS	POS	POS	POS	NEG	POS	POS	POS	NEG	POS	POS	POS	NEG	NEG	NEG
Strain Newman: in silico predicted hybridisation pattern	POS	POS	POS	POS	NEG	POS	POS	POS	NEG	POS	POS	POS	NEG	NEG	NEG
Beaver I_Austria_B2	POS	POS	POS	POS	NEG	POS	POS	POS	NEG	POS	POS	POS	NEG	NEG	NEG
Beaver J_Austria_B3	POS	POS	POS	POS	NEG	POS	POS	POS	NEG	POS	POS	POS	NEG	NEG	NEG
**>CC12-MSSA**															
Strain KLT6: in silico predicted hybridisation pattern	POS	POS	POS	POS	NEG	NEG	POS	POS	NEG	POS	POS	NEG	NEG	NEG	NEG
Strain 21266: in silico predicted hybridisation pattern	POS	POS	POS	POS	NEG	NEG	POS	POS	NEG	POS	POS	NEG	NEG	NEG	NEG
NP66 chromosome: in silico predicted hybridisation pattern	POS	POS	POS	POS	NEG	NEG	POS	POS	NEG	POS	POS	NEG	NEG	NEG	NEG
Beaver K_Austria_B4	POS	POS	POS	POS	NEG	NEG	POS	POS	NEG	POS	POS	NEG	NEG	NEG	NEG
**>CC49-MSSA**															
Strain 21262: in silico predicted hybridisation pattern	POS	POS	POS	POS	NEG	NEG	POS	POS	NEG	POS	POS	NEG	POS	NEG	NEG
Strain Tager 104: in silico predicted hybridisation pattern	POS	POS	POS	POS	NEG	NEG	POS	POS	NEG	POS	POS	NEG	POS	NEG	NEG
**>CC49-MSSA (lukF-P83/lukM+)**															
Strain 22_M61_07_10: in silico predicted hybridisation pattern	POS	POS	POS	POS	NEG	NEG	POS	POS	NEG	POS	POS	NEG	POS	NEG	NEG
Strain 22_M48_10_10: in silico predicted hybridisation pattern	POS	POS	POS	POS	NEG	NEG	POS	POS	NEG	POS	POS	NEG	POS	NEG	NEG
Strain 22_M47_10_10: in silico predicted hybridisation pattern	POS	POS	POS	POS	NEG	NEG	POS	POS	NEG	POS	POS	NEG	POS	NEG	NEG
**>CC49-MSSA (lukF-PV+/lukS-PV?)**															
Beaver C_Bavaria_WT65: in silico predicted hybridisation pattern	POS	POS	POS	POS	NEG	NEG	POS	POS	NEG	POS	POS	NEG	POS	NEG	NEG
Beaver C_Bavaria_WT65	POS	POS	POS	POS	NEG	NEG	POS	POS	NEG	POS	POS	NEG	POS	NEG	NEG
**>CC398-MSSA**															
Strain 21331: in silico predicted hybridisation pattern	POS	POS	NEG	POS	POS	POS	AMB	NEG	POS	NEG	NEG	NEG	NEG	NEG	NEG
Strain 71193: in silico predicted hybridisation pattern	POS	POS	NEG	POS	POS	POS	AMB	NEG	POS	NEG	AMB	NEG	NEG	NEG	NEG
Strain S123: in silico predicted hybridisation pattern	POS	POS	NEG	POS	POS	POS	AMB	NEG	POS	NEG	AMB	NEG	NEG	NEG	NEG
Beaver H_Austria_B1	POS	POS	NEG	POS	POS	POS	POS	NEG	POS	POS	POS	NEG	NEG	NEG	NEG
**>CC1956-MSSA**															
Strain C6589: in silico predicted hybridisation pattern	POS	POS	POS	POS	NEG	NEG	POS	POS	NEG	POS	POS	NEG	POS	NEG	NEG
Strain C6591: in silico predicted hybridisation pattern	POS	POS	POS	POS	NEG	NEG	POS	POS	NEG	POS	POS	NEG	POS	NEG	NEG
Strain C6597: in silico predicted hybridisation pattern	POS	POS	POS	POS	NEG	NEG	POS	POS	NEG	POS	POS	NEG	POS	NEG	NEG
Strain C6598: in silico predicted hybridisation pattern	POS	POS	POS	POS	NEG	NEG	POS	POS	NEG	POS	POS	NEG	POS	NEG	NEG
Strain C6601: in silico predicted hybridisation pattern	POS	POS	POS	POS	NEG	NEG	POS	POS	NEG	POS	POS	NEG	POS	NEG	NEG
**>CC1956-MSSA (lukF-PV+/lukS-PV?)**															
Beaver A_Berlin_WT19: in silico predicted hybridisation pattern	POS	POS	POS	POS	NEG	NEG	POS	POS	NEG	POS	POS	NEG	POS	NEG	NEG
Beaver A_Berlin_WT19	POS	POS	POS	POS	NEG	NEG	POS	POS	NEG	POS	POS	NEG	POS	NEG	NEG
Beaver B_Berlin_WT63	POS	POS	POS	POS	NEG	NEG	POS	POS	NEG	POS	POS	NEG	POS	NEG	NEG
Beaver B_Berlin_WT64	POS	POS	POS	POS	NEG	NEG	POS	POS	NEG	POS	POS	NEG	POS	NEG	NEG
Beaver D_Berlin_WT66	POS	POS	POS	POS	NEG	NEG	POS	POS	NEG	POS	POS	NEG	POS	NEG	NEG
Beaver D_Berlin_WT67A	POS	POS	POS	POS	NEG	NEG	POS	POS	NEG	POS	POS	NEG	POS	NEG	NEG
Beaver D_Berlin_WT67B	POS	POS	POS	POS	NEG	NEG	POS	POS	NEG	POS	POS	NEG	POS	NEG	NEG
Beaver D_Berlin_WT68	POS	POS	POS	POS	NEG	NEG	POS	POS	NEG	POS	POS	NEG	POS	NEG	NEG
Beaver D_Berlin_WT69	POS	POS	POS	POS	NEG	NEG	POS	POS	NEG	POS	POS	NEG	POS	NEG	NEG
Beaver F_Berlin_WT71	POS	POS	POS	POS	NEG	NEG	POS	POS	NEG	POS	POS	NEG	POS	NEG	NEG
Beaver E_Berlin_WT70	POS	POS	POS	POS	NEG	NEG	POS	POS	NEG	POS	POS	NEG	POS	NEG	NEG
Beaver G_Berlin_WT110	POS	POS	POS	POS	NEG	NEG	POS	POS	NEG	POS	POS	NEG	POS	NEG	NEG
Beaver G_Berlin_WT111	POS	POS	POS	POS	NEG	NEG	POS	POS	NEG	POS	POS	NEG	POS	NEG	NEG

STRAIN / ISOLATE	ADHAESION FACTORS / GENES ENCODING MICROBIAL SURFACE COMPONENTS RECOGNIZING ADHESIVE MATRIX MOLECULES (MSCRAMM GENES)																					
	fnbB							map				sasG				sasX / sesI	sdrC					
	fnbB	fnbB (COL)	fnbB (COL+Mu50+ MW2)	fnbB (Mu50)	fnbB (MW2)	fnbB (ST15)	fnbB (ST45-2)	map	map (COL)	map (MRSA252)	map (Mu50+MW2)	sasG	sasG (COL+Mu50)	sasG (MW2)	sasG (OtherThan252+122		sdrC	sdrC (B1)	sdrC (COL)	sdrC (Mu50)	sdrC (MW2+MRSA 252+RF122)	sdrC (OtherThan252+RF122)
	Fibronectin-binding protein B							Major histocompatibility complex class II analog protein (=Extracellular adherence protein, eap)				Staphylococcus aureus surface protein G					Ser-Asp rich fibrinogen-/bone sialoprotein-binding protein C					
**>CC8-MSSA**																						
Strain NCTC8325: in silico predicted hybridisation pattern	POS	AMB	AMB	NEG	NEG	NEG	NEG	POS	POS	NEG	NEG	POS	POS	NEG	POS	NEG	POS	NEG	POS	NEG	NEG	POS
Strain RN4220:VC40: in silico predicted hybridisation pattern	POS	AMB	AMB	NEG	NEG	NEG	NEG	POS	POS	NEG	NEG	POS	POS	NEG	POS	NEG	POS	NEG	POS	NEG	NEG	POS
Strain Newman: in silico predicted hybridisation pattern	POS	AMB	AMB	NEG	NEG	NEG	NEG	POS	POS	NEG	NEG	POS	POS	NEG	POS	NEG	POS	NEG	POS	NEG	NEG	POS
Beaver I_Austria_B2	POS	POS	AMB	NEG	NEG	NEG	NEG	POS	POS	NEG	NEG	POS	POS	NEG	POS		POS	NEG	POS	NEG	NEG	POS
Beaver J_Austria_B3	POS	POS	POS	NEG	NEG	NEG	NEG	POS	POS	NEG	NEG	POS	POS	NEG	POS		POS	NEG	POS	NEG	NEG	POS
**>CC12-MSSA**																						
Strain KLT6: in silico predicted hybridisation pattern	POS	NEG	AMB	NEG	NEG	NEG	AMB	POS	AMB	NEG	POS	NEG	NEG	NEG	NEG	NEG	POS	NEG	POS	NEG	NEG	POS
Strain 21266: in silico predicted hybridisation pattern	POS	NEG	AMB	NEG	NEG	NEG	AMB	POS	AMB	NEG	POS	NEG	NEG	NEG	NEG	NEG	POS	NEG	POS	NEG	NEG	POS
NP66 chromosome: in silico predicted hybridisation pattern	POS	NEG	AMB	NEG	NEG	NEG	AMB	POS	AMB	NEG	POS	NEG	NEG	NEG	NEG	NEG	POS	NEG	POS	NEG	NEG	POS
Beaver K_Austria_B4	NEG	NEG	NEG	NEG	NEG	NEG	NEG	POS	POS	NEG	POS	NEG	NEG	NEG	NEG		POS	NEG	POS	NEG	NEG	POS
**>CC49-MSSA**																						
Strain 21262: in silico predicted hybridisation pattern	POS	NEG	AMB	AMB	NEG	NEG	NEG	POS	POS	NEG	NEG	POS	NEG	NEG	POS	NEG	POS	NEG	POS	NEG	NEG	NEG
Strain Tager 104: in silico predicted hybridisation pattern	POS	NEG	AMB	AMB	NEG	NEG	NEG	POS	POS	NEG	NEG	POS	NEG	NEG	POS	NEG	POS	NEG	POS	NEG	NEG	NEG
**>CC49-MSSA (lukF-P83/lukM+)**																						
Strain 22_M61_07_10: in silico predicted hybridisation pattern	POS	NEG	AMB	AMB	NEG	NEG	NEG	POS	POS	NEG	NEG	POS	NEG	NEG	POS	NEG	POS	NEG	POS	NEG	NEG	NEG
Strain 22_M48_10_10: in silico predicted hybridisation pattern	POS	NEG	AMB	AMB	NEG	NEG	NEG	POS	POS	NEG	NEG	POS	NEG	NEG	POS	NEG	POS	NEG	POS	NEG	NEG	NEG
Strain 22_M47_10_10: in silico predicted hybridisation pattern	POS	NEG	AMB	AMB	NEG	NEG	NEG	POS	POS	NEG	NEG	POS	NEG	NEG	POS	NEG	POS	NEG	POS	NEG	NEG	NEG
**>CC49-MSSA (lukF-PV+/lukS-PV?)**																						
Beaver C_Bavaria_WT65: in silico predicted hybridisation pattern	POS	NEG	AMB	AMB	NEG	NEG	NEG	POS	POS	NEG	NEG	POS	NEG	NEG	POS	NEG	POS	NEG	POS	NEG	NEG	NEG
Beaver C_Bavaria_WT65	POS	NEG	POS	AMB	NEG	NEG	NEG	POS	POS	NEG	AMB	POS	POS	NEG	POS	NEG	POS	NEG	POS	NEG	NEG	NEG
**>CC398-MSSA**																						
Strain 21331: in silico predicted hybridisation pattern	POS	NEG	POS	NEG	NEG	NEG	NEG	NEG	NEG	AMB	NEG	NEG	NEG	NEG	NEG	NEG	POS	NEG	NEG	NEG	NEG	NEG
Strain 71193: in silico predicted hybridisation pattern	POS	NEG	POS	NEG	NEG	NEG	NEG	NEG	NEG	AMB	NEG	NEG	NEG	NEG	NEG	NEG	POS	NEG	NEG	NEG	NEG	NEG
Strain S123: in silico predicted hybridisation pattern	POS	NEG	POS	NEG	NEG	NEG	NEG	NEG	NEG	AMB	NEG	NEG	NEG	NEG	NEG	NEG	POS	NEG	NEG	NEG	NEG	NEG
Beaver H_Austria_B1	POS	NEG	POS	AMB	NEG	NEG	NEG	POS	POS	POS	NEG	NEG	NEG	NEG	NEG		POS	NEG	NEG	AMB	NEG	NEG
**>CC1956-MSSA**																						
Strain C6589: in silico predicted hybridisation pattern	POS	NEG	AMB	AMB	NEG	AMB	NEG	POS	POS	NEG	NEG	NEG	NEG	NEG	NEG	NEG	POS	NEG	POS	NEG	NEG	NEG
Strain C6591: in silico predicted hybridisation pattern	POS	NEG	AMB	AMB	NEG	AMB	NEG	POS	POS	NEG	NEG	NEG	NEG	NEG	NEG	NEG	POS	NEG	POS	NEG	NEG	NEG
Strain C6597: in silico predicted hybridisation pattern	POS	NEG	AMB	AMB	NEG	AMB	NEG	POS	POS	NEG	NEG	NEG	NEG	NEG	NEG	NEG	POS	NEG	POS	NEG	NEG	NEG
Strain C6598: in silico predicted hybridisation pattern	POS	NEG	AMB	AMB	NEG	AMB	NEG	POS	POS	NEG	NEG	NEG	NEG	NEG	NEG	NEG	POS	NEG	POS	NEG	NEG	NEG
Strain C6601: in silico predicted hybridisation pattern	POS	NEG	AMB	AMB	NEG	AMB	NEG	POS	POS	NEG	NEG	NEG	NEG	NEG	NEG	NEG	POS	NEG	POS	NEG	NEG	NEG
**>CC1956-MSSA (lukF-PV+/lukS-PV?)**																						
Beaver A_Berlin_WT19: in silico predicted hybridisation pattern	POS	NEG	AMB	AMB	NEG	AMB	NEG	POS	POS	NEG	NEG	NEG	NEG	NEG	NEG	NEG	POS	NEG	POS	NEG	NEG	NEG
Beaver A_Berlin_WT19	POS	NEG	POS	POS	NEG	AMB	NEG	POS	POS	NEG	POS	NEG	NEG	NEG	NEG	NEG	POS	NEG	POS	NEG	NEG	NEG
Beaver B_Berlin_WT63	POS	NEG	POS	AMB	NEG	AMB	NEG	POS	POS	NEG	POS	NEG	NEG	NEG	NEG	NEG	POS	NEG	POS	NEG	NEG	NEG
Beaver B_Berlin_WT64	POS	NEG	POS	AMB	NEG	AMB	NEG	POS	POS	NEG	POS	NEG	NEG	NEG	NEG		POS	NEG	POS	NEG	NEG	NEG
Beaver D_Berlin_WT66	POS	NEG	POS	AMB	NEG	AMB	NEG	POS	POS	NEG	POS	NEG	NEG	AMB	NEG		POS	NEG	POS	NEG	NEG	NEG
Beaver D_Berlin_WT67A	POS	NEG	POS	AMB	NEG	AMB	NEG	POS	POS	NEG	POS	NEG	NEG	AMB	NEG		POS	NEG	POS	NEG	NEG	NEG
Beaver D_Berlin_WT67B	POS	NEG	POS	AMB	NEG	AMB	NEG	POS	POS	NEG	POS	NEG	NEG	NEG	NEG	NEG	POS	NEG	POS	NEG	NEG	NEG
Beaver D_Berlin_WT68	POS	NEG	POS	AMB	NEG	AMB	NEG	POS	POS	NEG	POS	NEG	NEG	NEG	NEG	NEG	POS	NEG	POS	NEG	NEG	NEG
Beaver D_Berlin_WT69	POS	NEG	POS	AMB	NEG	AMB	NEG	POS	POS	NEG	POS	NEG	NEG	NEG	NEG		POS	NEG	POS	NEG	NEG	NEG
Beaver F_Berlin_WT71	POS	NEG	POS	AMB	NEG	AMB	NEG	POS	POS	NEG	POS	NEG	NEG	NEG	NEG		POS	NEG	POS	NEG	NEG	NEG
Beaver E_Berlin_WT70	POS	NEG	POS	AMB	NEG	AMB	NEG	POS	POS	NEG	AMB	NEG	NEG	NEG	NEG		POS	NEG	POS	NEG	NEG	NEG
Beaver G_Berlin_WT110	POS	NEG	POS	POS	NEG	NEG	NEG	POS	POS	NEG	NEG	NEG	NEG	NEG	NEG		POS	NEG	POS	NEG	NEG	NEG
Beaver G_Berlin_WT111	POS	NEG	POS	AMB	NEG	NEG	NEG	POS	POS	NEG	NEG	NEG	NEG	NEG	NEG		POS	NEG	POS	NEG	NEG	NEG

STRAIN / ISOLATE	ADHAESION FACTORS / MSCRAMM GENES											IMMUNOD.AG.B		DEFENSIN RESIST.		TRANSFERRIN BINDING PROT			PUTATIVE TRANSPORTER			
	sdrD					vwb						isaB		mprF		isdA			lmrP			
	sdrD	sdrD (cons)	sdrD (COL+MW2)	sdrD (Mu50)	sdrD (other)	vwb	vwb (cons)	vwb (COL+MW2)	vwb (MRSA252)	vwb (Mu50)	vwb (RF122)	isaB	isaB (MRSA252)	mprF (COL+MW2)	mprF (Mu50+252)	isdA (cons)	isdA (MRSA252)	isdA (Other Than MRSA252)	lmrP (OtherThanRF 122)	lmrP (OtherThanRF 122)	lmrP (RF122)	lmrP (RF122)
	Ser-Asp rich fibrinogen-/bone sialoprotein-binding protein D					van Willebrand factor binding protein						immunodominant antigen B		defensin resistance protein		transferrin-binding protein			hypothetical protein, similar to integral membrane protein LmrP			
**>CC8-MSSA**																						
Strain NCTC8325: in silico predicted hybridisation pattern	POS	POS	POS	NEG	NEG	POS	POS	POS	NEG	NEG	NEG	POS	NEG	POS	NEG	POS	NEG	POS	POS	POS	NEG	NEG
Strain RN4220:VC40: in silico predicted hybridisation pattern	POS	POS	POS	NEG	NEG	POS	POS	POS	NEG	NEG	NEG	POS	NEG	POS	NEG	POS	NEG	POS	POS	POS	NEG	NEG
Strain Newman: in silico predicted hybridisation pattern	POS	POS	POS	NEG	NEG	POS	POS	POS	NEG	NEG	NEG	POS	NEG	POS	NEG	POS	NEG	POS	POS	POS	NEG	NEG
Beaver I_Austria_B2	POS	POS	POS	NEG	NEG	POS	POS	POS	NEG	NEG	NEG	POS	AMB	POS	AMB	POS	NEG	POS	POS	POS	NEG	NEG
Beaver J_Austria_B3	POS	POS	POS	NEG	NEG	POS	POS	POS	NEG	NEG	NEG	POS	AMB	POS	NEG	POS	NEG	POS	POS	POS	NEG	NEG
**>CC12-MSSA**																						
Strain KLT6: in silico predicted hybridisation pattern	NEG	NEG	NEG	NEG	NEG	POS	POS	NEG	NEG	NEG	NEG	POS	NEG	POS	NEG	POS	NEG	POS	POS	POS	NEG	NEG
Strain 21266: in silico predicted hybridisation pattern	NEG	NEG	NEG	NEG	NEG	POS	POS	NEG	NEG	NEG	NEG	POS	NEG	POS	NEG	POS	NEG	POS	POS	POS	NEG	NEG
NP66 chromosome: in silico predicted hybridisation pattern	POS	POS	POS	NEG	NEG	POS	POS	NEG	NEG	NEG	NEG	POS	NEG	POS	NEG	POS	NEG	POS	POS	POS	NEG	NEG
Beaver K_Austria_B4	POS	POS	POS	NEG	NEG	POS	POS	NEG	NEG	NEG	NEG	POS	AMB	POS	NEG	POS	NEG	POS	POS	POS	NEG	NEG
**>CC49-MSSA**																						
Strain 21262: in silico predicted hybridisation pattern	POS	AMB	NEG	NEG	POS	POS	POS	NEG	NEG	NEG	NEG	POS	NEG	POS	NEG	POS	NEG	POS	NEG	NEG	POS	POS
Strain Tager 104: in silico predicted hybridisation pattern	POS	AMB	NEG	NEG	POS	POS	POS	NEG	NEG	NEG	NEG	POS	NEG	POS	NEG	POS	NEG	POS	NEG	NEG	POS	POS
**>CC49-MSSA (lukF-P83/lukM+)**																						
Strain 22_M61_07_10: in silico predicted hybridisation pattern	POS	AMB	NEG	NEG	POS	POS	POS	NEG	NEG	NEG	NEG	POS	NEG	POS	NEG	POS	NEG	POS	NEG	NEG	POS	POS
Strain 22_M48_10_10: in silico predicted hybridisation pattern	POS	AMB	NEG	NEG	POS	POS	POS	NEG	NEG	NEG	NEG	POS	NEG	POS	NEG	POS	NEG	POS	NEG	NEG	POS	POS
Strain 22_M47_10_10: in silico predicted hybridisation pattern	POS	AMB	NEG	NEG	POS	POS	POS	NEG	NEG	NEG	NEG	POS	NEG	POS	NEG	POS	NEG	POS	NEG	NEG	POS	POS
**>CC49-MSSA (lukF-PV+/lukS-PV?)**																						
Beaver C_Bavaria_WT65: in silico predicted hybridisation pattern	POS	AMB	NEG	NEG	POS	POS	POS	NEG	NEG	NEG	NEG	POS	NEG	POS	NEG	POS	NEG	POS	NEG	NEG	POS	POS
Beaver C_Bavaria_WT65	POS	POS	NEG	NEG	POS	POS	POS	NEG	NEG	NEG	NEG	POS	NEG	POS	AMB	POS	AMB	POS	NEG	NEG	POS	POS
**>CC398-MSSA**																						
Strain 21331: in silico predicted hybridisation pattern	POS	POS	NEG	AMB	NEG	POS	POS	NEG	NEG	NEG	NEG	NEG	POS	NEG	POS	POS	POS	NEG	POS	POS	NEG	NEG
Strain 71193: in silico predicted hybridisation pattern	POS	POS	NEG	AMB	NEG	POS	POS	NEG	NEG	NEG	NEG	NEG	POS	NEG	POS	POS	POS	NEG	POS	POS	NEG	NEG
Strain S123: in silico predicted hybridisation pattern	POS	POS	NEG	AMB	NEG	POS	POS	NEG	NEG	NEG	NEG	NEG	POS	NEG	POS	POS	POS	NEG	POS	POS	NEG	NEG
Beaver H_Austria_B1	POS	POS	NEG	NEG	NEG	POS	POS	NEG	NEG	NEG	NEG	NEG	POS	NEG	NEG	POS	POS	NEG	POS	POS	NEG	NEG
**>CC1956-MSSA**																						
Strain C6589: in silico predicted hybridisation pattern	NEG	NEG	NEG	NEG	NEG	POS	POS	POS	NEG	NEG	NEG	POS	NEG	NEG	POS	POS	NEG	POS	NEG	NEG	POS	POS
Strain C6591: in silico predicted hybridisation pattern	NEG	NEG	NEG	NEG	NEG	POS	POS	POS	NEG	NEG	NEG	POS	NEG	NEG	POS	POS	NEG	POS	NEG	NEG	POS	POS
Strain C6597: in silico predicted hybridisation pattern	NEG	NEG	NEG	NEG	NEG	POS	POS	POS	NEG	NEG	NEG	POS	NEG	NEG	POS	POS	NEG	POS	NEG	NEG	POS	POS
Strain C6598: in silico predicted hybridisation pattern	NEG	NEG	NEG	NEG	NEG	POS	POS	POS	NEG	NEG	NEG	POS	NEG	NEG	POS	POS	NEG	POS	NEG	NEG	POS	POS
Strain C6601: in silico predicted hybridisation pattern	NEG	NEG	NEG	NEG	NEG	POS	POS	POS	NEG	NEG	NEG	POS	NEG	NEG	POS	POS	NEG	POS	NEG	NEG	POS	POS
**>CC1956-MSSA (lukF-PV+/lukS-PV?)**																						
Beaver A_Berlin_WT19: in silico predicted hybridisation pattern	NEG	NEG	NEG	NEG	NEG	POS	POS	POS	NEG	NEG	NEG	POS	NEG	NEG	POS	POS	NEG	POS	NEG	NEG	POS	POS
Beaver A_Berlin_WT19	NEG	NEG	NEG	NEG	NEG	POS	POS	POS	NEG	NEG	NEG	POS	AMB	NEG	NEG	POS	NEG	POS	NEG	NEG	POS	POS
Beaver B_Berlin_WT63	NEG	NEG	NEG	NEG	NEG	POS	POS	POS	NEG	NEG	NEG	POS	AMB	NEG	NEG	POS	AMB	POS	NEG	NEG	POS	POS
Beaver B_Berlin_WT64	NEG	NEG	NEG	NEG	NEG	POS	POS	POS	NEG	NEG	NEG	POS	AMB	NEG	NEG	POS	AMB	POS	NEG	NEG	POS	POS
Beaver D_Berlin_WT66	NEG	NEG	NEG	NEG	NEG	POS	POS	POS	NEG	NEG	NEG	POS	AMB	NEG	NEG	POS	AMB	POS	NEG	NEG	POS	POS
Beaver D_Berlin_WT67A	NEG	NEG	NEG	NEG	NEG	POS	POS	POS	NEG	NEG	NEG	POS	AMB	NEG	POS	POS	AMB	POS	NEG	NEG	POS	POS
Beaver D_Berlin_WT67B	NEG	NEG	NEG	NEG	NEG	POS	POS	POS	NEG	NEG	NEG	POS	AMB	NEG	NEG	POS	NEG	POS	NEG	NEG	POS	POS
Beaver D_Berlin_WT68	NEG	NEG	NEG	NEG	NEG	POS	POS	POS	NEG	NEG	NEG	POS	AMB	NEG	POS	POS	AMB	POS	NEG	NEG	POS	POS
Beaver D_Berlin_WT69	NEG	NEG	NEG	NEG	NEG	POS	POS	POS	NEG	NEG	NEG	POS	AMB	NEG	NEG	POS	NEG	POS	NEG	NEG	POS	POS
Beaver F_Berlin_WT71	NEG	NEG	NEG	NEG	NEG	POS	POS	POS	NEG	NEG	NEG	POS	AMB	NEG	NEG	POS	AMB	POS	NEG	NEG	POS	POS
Beaver E_Berlin_WT70	NEG	NEG	NEG	NEG	NEG	POS	POS	POS	NEG	NEG	NEG	POS	AMB	NEG	NEG	POS	NEG	POS	NEG	NEG	POS	POS
Beaver G_Berlin_WT110	NEG	NEG	NEG	NEG	NEG	POS	POS	POS	NEG	NEG	NEG	POS	AMB	NEG	NEG	POS	NEG	POS	NEG	NEG	POS	POS
Beaver G_Berlin_WT111	NEG	NEG	NEG	NEG	NEG	POS	POS	POS	NEG	NEG	NEG	POS	AMB	NEG	NEG	POS	NEG	POS	NEG	NEG	POS	POS

STRAIN / ISOLATE	TYPE I RESTRICTION-MODIFICATION SYSTEM, SINGLE SEQUENCE SPECIFICITY PROTEIN													MISCELLANEOUS GENES							
	hsdS1	hsdS2				hsdS3					hsdSx							Q9RL82			
	hsdS1-RF122	hsdS2-ST5+ST8	hsdS2-MW2+476	hsdS2-RF122	hsdS2-MRSA252	hsdS3-AllOtherThan RF122+252	hsdS3-ST8+ST1+RF122	hsdS3-Mu50+N315	hsdS3-CC51+252	hsdS3-MRSA252	hsdSx-CC25	hsdSx-CC15	hsdSx-etd	ear2 = Q2FXC0	Q2YUB3	Q7A4X2	Q931R4 (CC5, CC15, CC30, CC97, CC188, ST1850)	Q9RL82 (consensus)	Q9RL82 (CC8)	Q9RL82-CC10/361	Q2G1R6-genomic island / cstB
	type I site-specific deoxyribo-nuclease subunit, 1st locus	type I site-specific deoxyribonuclease subunit, 2nd locus				type I site-specific deoxyribonuclease subunit, 3rd locus					type I site-specific deoxyribonuclease subunit, unknown locus			Putative protein	Multidrug resistance transporter	Putative protein	major facilitator superfamily transporter	Putative protein			
**>CC8-MSSA**																					
Strain NCTC8325: in silico predicted hybridisation pattern	NEG	POS	NEG	NEG	NEG	POS	POS	NEG	NEG	NEG	POS	NEG	NEG	POS	NEG	NEG	NEG	POS	POS	NEG	POS
Strain RN4220:VC40: in silico predicted hybridisation pattern	NEG	POS	NEG	NEG	NEG	POS	POS	NEG	NEG	NEG	POS	NEG	NEG	POS	NEG	NEG	NEG	POS	POS	NEG	POS
Strain Newman: in silico predicted hybridisation pattern	NEG	POS	NEG	NEG	NEG	POS	POS	NEG	NEG	NEG	POS	NEG	NEG	POS	NEG	NEG	NEG	POS	POS	NEG	POS
Beaver I_Austria_B2	NEG	POS	NEG	NEG	NEG	POS	POS	NEG	NEG	NEG	POS	NEG	NEG	POS	NEG	NEG					
Beaver J_Austria_B3	NEG	POS	NEG	NEG	NEG	POS	POS	NEG	NEG	NEG	POS	NEG	NEG	POS	NEG	NEG					
**>CC12-MSSA**																					
Strain KLT6: in silico predicted hybridisation pattern	NEG	POS	NEG	NEG	NEG	NEG	NEG	NEG	POS	NEG	POS	NEG	NEG	NEG	NEG	NEG	NEG	POS	POS	NEG	POS
Strain 21266: in silico predicted hybridisation pattern	NEG	POS	NEG	NEG	NEG	NEG	NEG	NEG	POS	NEG	NEG	NEG	NEG	NEG	NEG	NEG	NEG	POS	POS	NEG	POS
NP66 chromosome: in silico predicted hybridisation pattern	NEG	POS	NEG	NEG	NEG	NEG	NEG	NEG	POS	NEG	POS	NEG	NEG	NEG	NEG	NEG	NEG	POS	POS	NEG	POS
Beaver K_Austria_B4	NEG	POS	NEG	NEG	NEG	NEG	NEG	NEG	POS	NEG	POS	NEG	NEG	NEG	NEG	NEG					
**>CC49-MSSA**																					
Strain 21262: in silico predicted hybridisation pattern	NEG	NEG	NEG	NEG	NEG	NEG	NEG	NEG	POS	NEG	NEG	POS	POS	NEG	NEG	NEG	NEG	NEG	NEG	NEG	NEG
Strain Tager 104: in silico predicted hybridisation pattern	NEG	NEG	NEG	NEG	NEG	NEG	NEG	NEG	POS	NEG	NEG	POS	POS	NEG	NEG	NEG	NEG	NEG	NEG	NEG	NEG
**>CC49-MSSA (lukF-P83/lukM+)**																					
Strain 22_M61_07_10: in silico predicted hybridisation pattern	NEG	NEG	NEG	NEG	NEG	NEG	NEG	NEG	POS	NEG	NEG	POS	POS	NEG	NEG	NEG	NEG	NEG	NEG	NEG	NEG
Strain 22_M48_10_10: in silico predicted hybridisation pattern	NEG	NEG	NEG	NEG	NEG	NEG	NEG	NEG	POS	NEG	NEG	POS	POS	NEG	NEG	NEG	NEG	NEG	NEG	NEG	NEG
Strain 22_M47_10_10: in silico predicted hybridisation pattern	NEG	NEG	NEG	NEG	NEG	NEG	NEG	NEG	POS	NEG	NEG	POS	POS	NEG	NEG	NEG	NEG	NEG	NEG	NEG	NEG
**>CC49-MSSA (lukF-PV+/lukS-PV?)**																					
Beaver C_Bavaria_WT65: in silico predicted hybridisation pattern	NEG	NEG	NEG	NEG	NEG	NEG	NEG	NEG	POS	NEG	NEG	POS	POS	NEG	NEG	NEG	NEG	NEG	NEG	NEG	NEG
Beaver C_Bavaria_WT65	NEG	NEG	NEG	NEG	NEG	NEG	NEG	NEG	POS	NEG	POS	POS	POS	NEG	NEG	NEG	NEG	NEG	NEG	NEG	NEG
**>CC398-MSSA**																					
Strain 21331: in silico predicted hybridisation pattern	NEG	NEG	NEG	NEG	NEG	NEG	NEG	NEG	NEG	NEG	NEG	POS	NEG	NEG	NEG	NEG	NEG	NEG	NEG	NEG	NEG
Strain 71193: in silico predicted hybridisation pattern	NEG	NEG	NEG	NEG	NEG	NEG	NEG	NEG	NEG	NEG	NEG	POS	NEG	NEG	NEG	NEG	NEG	NEG	NEG	NEG	NEG
Strain S123: in silico predicted hybridisation pattern	NEG	NEG	NEG	NEG	NEG	NEG	NEG	NEG	NEG	NEG	NEG	POS	NEG	NEG	NEG	NEG	NEG	NEG	NEG	NEG	NEG
Beaver H_Austria_B1	NEG	NEG	NEG	NEG	NEG	NEG	NEG	NEG	NEG	NEG	NEG	POS	NEG	NEG	NEG	NEG					
**>CC1956-MSSA**																					
Strain C6589: in silico predicted hybridisation pattern	NEG	NEG	NEG	NEG	NEG	NEG	NEG	NEG	POS	NEG	NEG	POS	POS	NEG	NEG	NEG	POS	POS	POS	NEG	POS
Strain C6591: in silico predicted hybridisation pattern	NEG	NEG	NEG	NEG	NEG	NEG	NEG	NEG	POS	NEG	NEG	POS	POS	NEG	NEG	NEG	POS	POS	POS	NEG	POS
Strain C6597: in silico predicted hybridisation pattern	NEG	NEG	NEG	NEG	NEG	NEG	NEG	NEG	POS	NEG	NEG	POS	POS	NEG	NEG	NEG	POS	POS	POS	NEG	POS
Strain C6598: in silico predicted hybridisation pattern	NEG	NEG	NEG	NEG	NEG	NEG	NEG	NEG	POS	NEG	NEG	POS	POS	NEG	NEG	NEG	POS	POS	POS	NEG	POS
Strain C6601: in silico predicted hybridisation pattern	NEG	NEG	NEG	NEG	NEG	NEG	NEG	NEG	POS	NEG	NEG	POS	POS	NEG	NEG	NEG	POS	POS	POS	NEG	POS
**>CC1956-MSSA (lukF-PV+/lukS-PV?)**																					
Beaver A_Berlin_WT19: in silico predicted hybridisation pattern	NEG	NEG	NEG	NEG	NEG	NEG	NEG	NEG	POS	NEG	NEG	POS	POS	NEG	NEG	NEG	POS	POS	POS	NEG	POS
Beaver A_Berlin_WT19	NEG	NEG	NEG	NEG	NEG	POS	NEG	NEG	POS	NEG	NEG	POS	POS	NEG	NEG	NEG	POS	POS	POS	NEG	POS
Beaver B_Berlin_WT63	NEG	NEG	NEG	NEG	NEG	POS	NEG	NEG	POS	NEG	NEG	POS	POS	NEG	NEG	NEG	POS	POS	POS	NEG	POS
Beaver B_Berlin_WT64	NEG	NEG	NEG	NEG	NEG	POS	NEG	NEG	POS	NEG	NEG	POS	POS	NEG	NEG	NEG					
Beaver D_Berlin_WT66	NEG	NEG	NEG	NEG	NEG	POS	NEG	NEG	POS	NEG	NEG	POS	POS	NEG	NEG	NEG					
Beaver D_Berlin_WT67A	NEG	NEG	NEG	NEG	NEG	POS	NEG	NEG	POS	NEG	AMB	POS	POS	NEG	NEG	NEG					
Beaver D_Berlin_WT67B	NEG	NEG	NEG	NEG	NEG	POS	NEG	NEG	POS	NEG	NEG	POS	POS	NEG	NEG	NEG	POS	POS	POS	NEG	POS
Beaver D_Berlin_WT68	NEG	NEG	NEG	NEG	NEG	POS	NEG	NEG	POS	NEG	NEG	POS	POS	NEG	NEG	NEG	POS	POS	POS	NEG	POS
Beaver D_Berlin_WT69	NEG	NEG	NEG	NEG	NEG	POS	NEG	NEG	POS	NEG	NEG	POS	POS	NEG	NEG	NEG					
Beaver F_Berlin_WT71	NEG	NEG	NEG	NEG	NEG	POS	NEG	NEG	POS	NEG	NEG	POS	POS	NEG	NEG	NEG					
Beaver E_Berlin_WT70	NEG	NEG	NEG	NEG	NEG	POS	NEG	NEG	POS	NEG	NEG	POS	POS	NEG	NEG	NEG					
Beaver G_Berlin_WT110	NEG	NEG	NEG	NEG	NEG	NEG	NEG	NEG	POS	NEG	NEG	POS	POS	NEG	NEG	NEG					
Beaver G_Berlin_WT111	NEG	NEG	NEG	NEG	NEG	NEG	NEG	NEG	POS	NEG	NEG	POS	POS	NEG	NEG	NEG					

STRAIN / ISOLATE	MISCELLANEOUS GENES											HYALURONATE LYASE							
	sau				sau96I	G7ZRU6	ycjY	sagD	G7ZTC1		sdrM / tetEfflux	hysA1			hysA2				
	sau3AI	sauUSI	sauRF122	sauSO385					G7ZTC1	G7ZTC1-argenteus		hysA1 (MRSA252)	hysA1 (MRSA252+RF122) and/or hysA2 (cons)	hysA1 (MRSA252+RF122) and/or hysA2 (COL+USA300)	hysA2 (All Other Than MRSA252)	hysA2 (COL+USA300 +NCTC8325)	hysA2 (All Other Than COL+USA300+ NCTC8325)	hysA2 (All Other Than COL+USA300+ NCTC8325)	hysA2 (MRSA252)
	type II restriction-modification system endonuclease					acetyltransferase, GNAT family, "Argenteus/ST1850-like"	Marker for "Argenteus/ST1850-like", CC12, CC361, CC398	Putative bacteriocin biosynthesis associated protein	TetR family regulator		Multidrug resistance transporter	Hyaluronate lyase, variable first / second locus			Hyaluronate lyase, second locus				
**>CC8-MSSA**																			
Strain NCTC8325: in silico predicted hybridisation pattern	NEG	POS	NEG	NEG	NEG	NEG	NEG	NEG	NEG	NEG	POS	NEG	POS	NEG	POS	POS	NEG	NEG	NEG
Strain RN4220:VC40: in silico predicted hybridisation pattern	NEG	POS	NEG	NEG	NEG	NEG	NEG	NEG	NEG	NEG	POS	NEG	POS	NEG	POS	POS	NEG	NEG	NEG
Strain Newman: in silico predicted hybridisation pattern	NEG	POS	NEG	NEG	NEG	NEG	NEG	NEG	NEG	NEG	POS	NEG	POS	NEG	POS	POS	NEG	NEG	NEG
Beaver I_Austria_B2											POS	NEG	POS	POS	POS	POS	NEG	NEG	NEG
Beaver J_Austria_B3											POS	NEG	POS	POS	POS	POS	NEG	NEG	NEG
**>CC12-MSSA**																			
Strain KLT6: in silico predicted hybridisation pattern	NEG	POS	NEG	NEG	NEG	NEG	POS	NEG	POS	NEG	POS	NEG	POS	NEG	NEG	NEG	POS	AMB	NEG
Strain 21266: in silico predicted hybridisation pattern	NEG	POS	NEG	NEG	NEG	NEG	POS	NEG	POS	NEG	POS	NEG	POS	NEG	NEG	NEG	POS	AMB	NEG
NP66 chromosome: in silico predicted hybridisation pattern	NEG	POS	NEG	NEG	NEG	NEG	POS	NEG	POS	NEG	POS	NEG	POS	NEG	NEG	NEG	POS	AMB	NEG
Beaver K_Austria_B4											POS	NEG	POS	NEG	NEG	NEG	POS	POS	NEG
**>CC49-MSSA**																			
Strain 21262: in silico predicted hybridisation pattern	NEG	POS	NEG	NEG	NEG	NEG	NEG	NEG	NEG	NEG	POS	NEG	POS	NEG	NEG	NEG	NEG	NEG	NEG
Strain Tager 104: in silico predicted hybridisation pattern	NEG	POS	NEG	NEG	NEG	NEG	NEG	NEG	NEG	NEG	POS	POS	POS	NEG	NEG	NEG	AMB	POS	NEG
**>CC49-MSSA (lukF-P83/lukM+)**																			
Strain 22_M61_07_10: in silico predicted hybridisation pattern	NEG	POS	NEG	NEG	NEG	NEG	NEG	NEG	NEG	NEG	POS	POS	POS	NEG	NEG	NEG	AMB	POS	NEG
Strain 22_M48_10_10: in silico predicted hybridisation pattern	NEG	POS	NEG	NEG	NEG	NEG	NEG	NEG	NEG	NEG	POS	POS	POS	NEG	NEG	NEG	AMB	POS	NEG
Strain 22_M47_10_10: in silico predicted hybridisation pattern	NEG	POS	NEG	NEG	NEG	NEG	NEG	NEG	NEG	NEG	POS	POS	POS	NEG	NEG	NEG	AMB	POS	NEG
**>CC49-MSSA (lukF-PV+/lukS-PV?)**																			
Beaver C_Bavaria_WT65: in silico predicted hybridisation pattern	NEG	POS	NEG	NEG	NEG	NEG	NEG	NEG	NEG	NEG	POS	POS	POS	NEG	NEG	NEG	AMB	POS	NEG
Beaver C_Bavaria_WT65	NEG	POS	NEG	NEG	NEG	NEG	NEG	NEG	NEG	NEG	POS	POS	POS	NEG	NEG	NEG	POS	POS	NEG
**>CC398-MSSA**																			
Strain 21331: in silico predicted hybridisation pattern	NEG	NEG	NEG	POS	NEG	NEG	POS	NEG	POS	NEG	POS	NEG	POS	AMB	NEG	POS	NEG	NEG	POS
Strain 71193: in silico predicted hybridisation pattern	NEG	NEG	NEG	POS	NEG	NEG	POS	NEG	POS	NEG	POS	NEG	POS	AMB	NEG	POS	NEG	NEG	POS
Strain S123: in silico predicted hybridisation pattern	NEG	NEG	NEG	POS	NEG	NEG	POS	NEG	POS	NEG	POS	NEG	POS	AMB	NEG	POS	NEG	NEG	POS
Beaver H_Austria_B1											POS	NEG	POS	POS	NEG	POS	NEG	NEG	NEG
**>CC1956-MSSA**																			
Strain C6589: in silico predicted hybridisation pattern	NEG	POS	NEG	NEG	NEG	NEG	NEG	NEG	NEG	NEG	POS	POS	POS	AMB	NEG	POS	NEG	NEG	NEG
Strain C6591: in silico predicted hybridisation pattern	NEG	POS	NEG	NEG	NEG	NEG	NEG	NEG	NEG	NEG	POS	POS	POS	AMB	NEG	POS	NEG	NEG	NEG
Strain C6597: in silico predicted hybridisation pattern	NEG	POS	NEG	NEG	NEG	NEG	NEG	NEG	NEG	NEG	POS	POS	POS	AMB	NEG	POS	NEG	NEG	NEG
Strain C6598: in silico predicted hybridisation pattern	NEG	POS	NEG	NEG	NEG	NEG	NEG	NEG	NEG	NEG	POS	POS	POS	AMB	NEG	POS	NEG	NEG	NEG
Strain C6601: in silico predicted hybridisation pattern	NEG	POS	NEG	NEG	NEG	NEG	NEG	NEG	NEG	NEG	POS	POS	POS	AMB	NEG	POS	NEG	NEG	NEG
**>CC1956-MSSA (lukF-PV+/lukS-PV?)**																			
Beaver A_Berlin_WT19: in silico predicted hybridisation pattern	NEG	POS	NEG	NEG	NEG	NEG	NEG	NEG	NEG	NEG	POS	POS	POS	AMB	NEG	POS	NEG	NEG	NEG
Beaver A_Berlin_WT19	NEG	POS	NEG	NEG	NEG	NEG	NEG	NEG	NEG	NEG	POS	POS	POS	POS	NEG	POS	NEG	NEG	NEG
Beaver B_Berlin_WT63	NEG	POS	NEG	NEG	NEG	NEG	NEG	NEG	NEG	NEG	POS	POS	POS	POS	NEG	POS	NEG	NEG	NEG
Beaver B_Berlin_WT64											POS	POS	POS	POS	NEG	POS	NEG	NEG	NEG
Beaver D_Berlin_WT66											POS	POS	POS	POS	NEG	POS	NEG	NEG	NEG
Beaver D_Berlin_WT67A											POS	POS	POS	POS	NEG	POS	NEG	NEG	NEG
Beaver D_Berlin_WT67B	NEG	POS	NEG	NEG	NEG	NEG	NEG	NEG	NEG	NEG	POS	POS	POS	POS	NEG	POS	NEG	NEG	NEG
Beaver D_Berlin_WT68	NEG	POS	NEG	NEG	NEG	NEG	NEG	NEG	NEG	NEG	POS	POS	POS	POS	NEG	POS	NEG	NEG	NEG
Beaver D_Berlin_WT69											POS	POS	POS	POS	NEG	POS	NEG	NEG	NEG
Beaver F_Berlin_WT71											POS	POS	POS	POS	NEG	POS	NEG	NEG	NEG
Beaver E_Berlin_WT70											POS	POS	POS	POS	NEG	POS	NEG	NEG	NEG
Beaver G_Berlin_WT110											POS	POS	POS	POS	NEG	POS	NEG	NEG	NEG
Beaver G_Berlin_WT111											POS	POS	POS	POS	NEG	POS	NEG	NEG	NEG
