# Supplementary material for: Staphylococcus aureus isolates from Eurasian Beavers (Castor fiber) carry a novel phage-borne bicomponent leukocidin related to the Panton-Valentine leukocidin
Source: Sci Rep. 2021 Dec 22;11:24394. doi: 10.1038/s41598-021-03823-6 (PMC8695587; doi:10.1038/s41598-021-03823-6)
Supplement: Supplementary file 1 — Supplementary Information. [file 41598_2021_3823_MOESM1_ESM.zip › Supplemental File 3a_Results of the antimicrobial susceptibility testing.pdf]

Supplemental File 3a: Results of the antimicrobial susceptibility testing

1) Data for the CC49/CC1956 *S. aureus* isolates from Germany (animals A-G).

**Antimicrobial agent**	**MIC (in µg/mL)**																	
	**0.008**	**0.015**	**0.03**	**0.06**	**0.12**	**0.25**	**0.5**	**1**	**2**	**4**	**8**	**16**	**32**	**64**	**128**	**256**	**512**	**1024**
Oxacillin		2	1	-	1	4	5	-	-	-	-							
Penicillin		10	3	-	-	-	-	-	-	-	-	-	-					
Ampicillin			2	7	4	-	-	-	-	-	-	-	-	-				
Amoxicillin/ clavulanic acid[a]			1	1	6	5	-	-	-	-	-	-	-	-				
Imipenem		10	3	-	-	-	-	-	-	-	-	-	-					
Ceftiofur			-	-	-	-	2	11	-	-	-	-	-	-				
Cefquinom		-	-	-	-	2	6	5	-	-	-	-	-					
Cefalothin				2	2	8	1	-	-	-	-	-	-	-	-			
Cefotaxime		-	-	-	-	-	-	3	9	1	-	-	-					
Cefoperazone				-	-	1	-	2	9	1	-	-	-					
Erythromycin		-	-	-	-	8	5	-	-	-	-	-	-					
Tylosin tartrate				-	-	-	-	1	3	5	4	-	-	-	-			
Tulathromycin				-	-	-	1	7	3	2	-	-	-					
Tilmicosin				-	-	-	2	10	1	-	-	-	-	-	-			
Clindamycin			-	-	11	2	-	-	-	-	-	-	-	-				
Pirlimycin			-	-	-	4	2	7	-	-	-	-	-	-				
Tiamulin			-	-	-	-	6	7	-	-	-	-	-	-				
Ciprofloxacin	-	1	1	3	5	2	-	1	-	-	-	-						
Enrofloxacin	-	1	1	7	4	-	-	-	-	-	-	-						
Marbofloxacin	-	-	-	2	7	3	1	-	-	-	-	-						
Nalidixic acid				-	-	-	-	-	-	-	1	8	3	1	-			
Gentamicin					-	7	5	1	-	-	-	-	-	-	-	-		
Streptomicin						-	-	-	-	-	11	2	-	-	-	-	-	
Neomycin					-	3	8	2	-	-	-	-	-	-				
Tetracycline					1	9	2	1	-	-	-	-	-	-	-	-		
Doxycyclin				1	8	4	-	-	-	-	-	-	-	-	-			
Sulfamethoxazole/ trimethoprim[b]		-	5	8	-	-	-	-	-	-	-	-	-					
Florfenicol					-	-	-	-	2	8	3	-	-	-	-	-		
Linezolid			-	-	-	-	-	5	7	1	-	-	-	-				
Vancomycin		-	-	-	-	-	2	10	1	-	-	-	-					
Quinupristin/ Dalfopristin		-	-	-	-	4	9	-	-	-	-	-	-					

The grey-shaded areas represent concentrations not included in the test panels.

[a]The MIC values of amoxicillin/clavulanic acid (2:1) are expressed as the MIC values of amoxicillin.

[b]The MIC values of trimethoprim/sulfamethoxazole (1:19) are expressed as the MIC values of trimethoprim.

2) Data for the non-CC49/CC1956 *S. aureus* isolates from Austria (animals H-K).

Antimicrobial agent	MIC (in µg/mL)																	
	**0.008**	**0.015**	**0.03**	**0.06**	**0.12**	**0.25**	**0.5**	**1**	**2**	**4**	**8**	**16**	**32**	**64**	**128**	**256**	**512**	**1024**
Oxacillin		-	-	-	-	3	1	-	-	-	-	-						
Penicillin		-	1	-	-	-	-	-	3	-	-	-	-	-				
Ampicillin			-	-	1	-	-	1	2	-	-	-	-	-	-			
Amoxicillin/ clavulanic acid [a]			-	-	-	1	1	2	-	-	-	-	-	-	-			
Imipenem		3	1	-	-	-	-	-	-	-	-	-	-	-				
Ceftiofur			-	-	-	-	1	3	-	-	-	-	-	-	-			
Cefquinom		-	-	-	-	-	1	2	1	-	-	-	-	-				
Cefalothin				-	-	2	2	-	-	-	-	-	-	-	-	-		
Cefotaxime		-	-	-	-	-	-	1	1	2	-	-	-	-				
Cefoperazone				-	-	-	-	1	1	2	-	-	-	-				
Erythromycin		-	-	-	-	2	-	-	-	-	-	-	-	2				
Tylosin tartrate				-	-	-	-	-	3	-	-	1	-	-	-	-		
Tulathromycin				-	-	-	-	-	-	2	1	-	-	1				
Tilmicosin				-	-	-	-	3	-	-	-	-	1	-	-	-		
Clindamycin			-	-	3	-	-	-	-	-	-	-	-	-	1			
Pirlimycin			-	-	-	-	1	2	-	-	-	-	-	-	1			
Tiamulin			-	-	-	2	1	-	1	-	-	-	-	-	-			
Ciprofloxacin	-	-	-	-	1	1	2	-	-	-	-	-	-					
Enrofloxacin	-	-	-	-	4	-	-	-	-	-	-	-	-					
Marbofloxacin	-	-	-	-	-	3	1	-	-	-	-	-	-					
Nalidixic acid				-	-	-	-	-	-	-	-	-	4	-	-	-		
Gentamicin					-	1	3	-	-	-	-	-	-	-	-	-	-	
Streptomicin						-	-	-	-	-	3	-	1	-	-	-	-	-
Neomycin					-	-	4	-	-	-	-	-	-	-	-			
Tetracycline					-	3	-	-	-	-	-	-	-	1	-	-	-	
Doxycyclin				-	1	1	-	1	-	-	-	1	-	-	-	-		
Sulfamethoxazole/ trimethoprim[b]		-	-	3	-	-	1	-	-	-	-	-	-	-				
Florfenicol					-	-	-	-	-	4	-	-	-	-	-	-	-	
Linezolid			-	-	-	-	-	1	3	-	-	-	-	-	-			
Vancomycin		-	-	-	-	-	-	4	-	-	-	-	-	-				
Quinupristin/ Dalfopristin		-	-	-	-	1	2	1	-	-	-	-	-	-				

The grey-shaded areas represent concentrations not included in the test panels.

[a]The MIC values of amoxicillin/clavulanic acid (2:1) are expressed as the MIC values of amoxicillin.

[b]The MIC values of trimethoprim/sulfamethoxazole (1:19) are expressed as the MIC values of trimethoprim.
