# Supplementary material for: Staphylococcus aureus isolates from Eurasian Beavers (Castor fiber) carry a novel phage-borne bicomponent leukocidin related to the Panton-Valentine leukocidin
Source: Sci Rep. 2021 Dec 22;11:24394. doi: 10.1038/s41598-021-03823-6 (PMC8695587; doi:10.1038/s41598-021-03823-6)

Supplemental File 3b: Results of the biocide susceptibility testing

1) Data for the CC49/CC1956 *S. aureus* isolates from Germany (animals A-G).

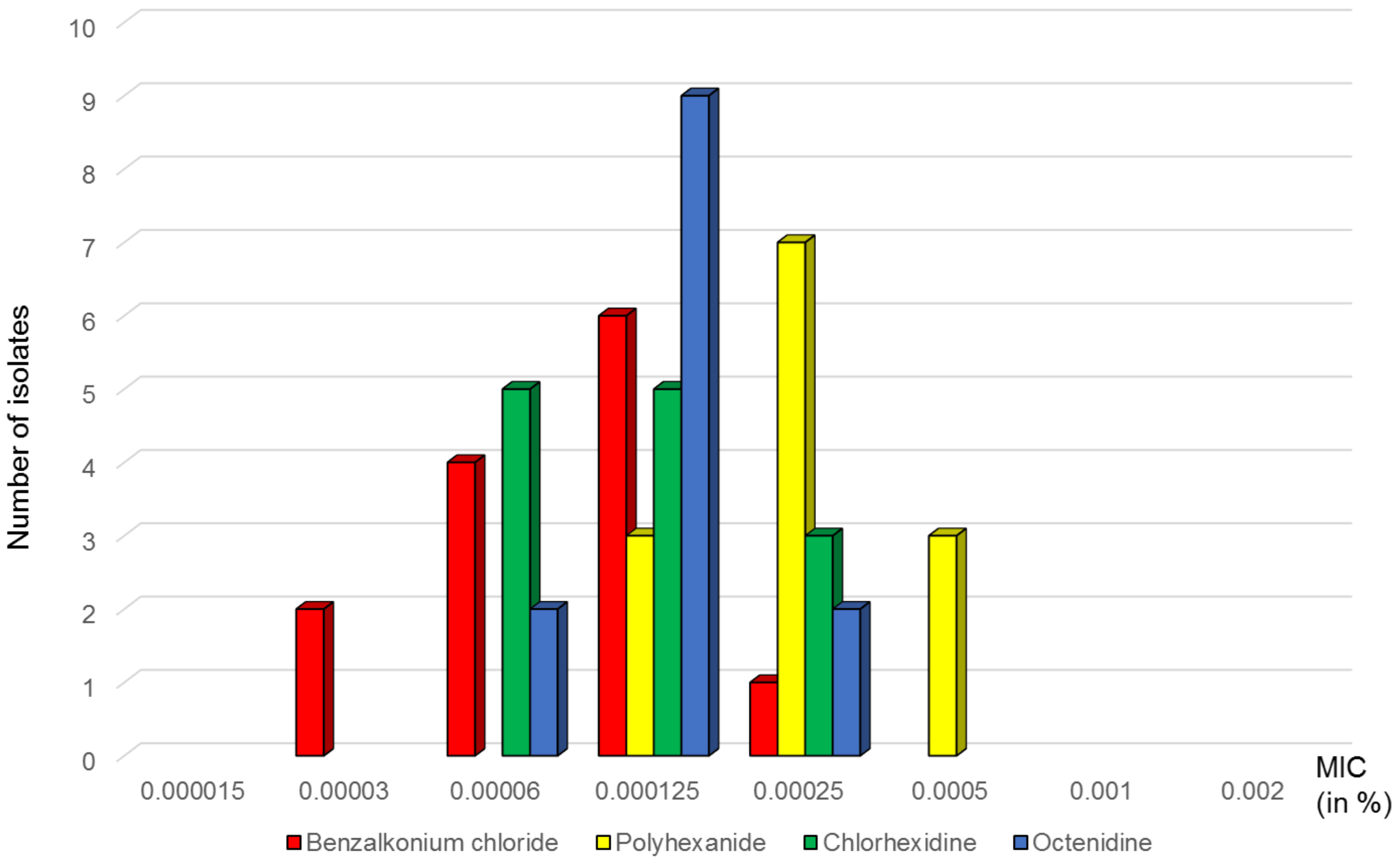

2) Data for the non-CC49/CC1956 *S. aureus* isolates from Austria (animals H-K).

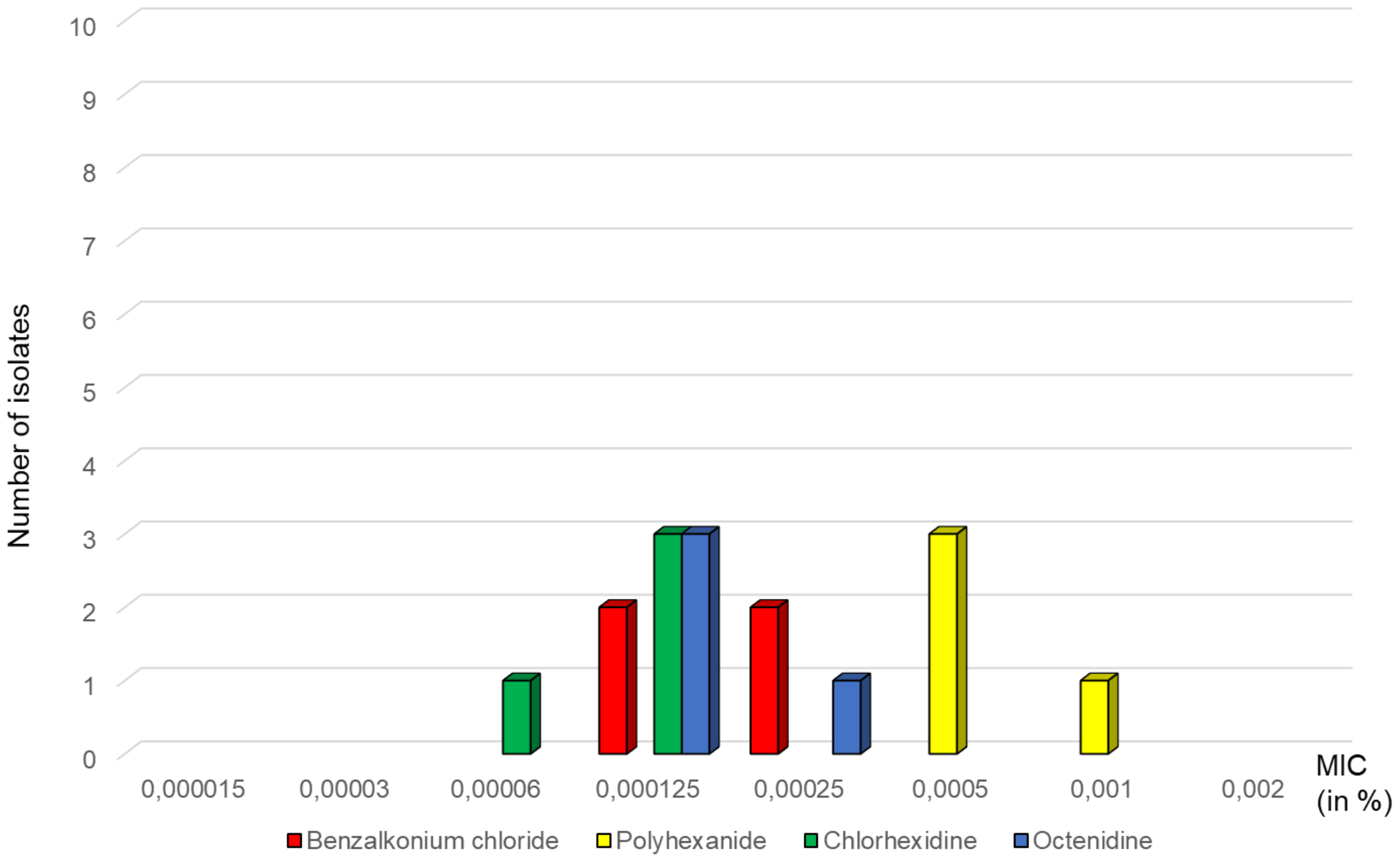

Supplement: Supplementary file 1 — Supplementary Information. [file 41598_2021_3823_MOESM1_ESM.zip › Supplemental File 3b_Biocide MICs.pdf]
