# Supplementary material for: Staphylococcus aureus isolates from Eurasian Beavers (Castor fiber) carry a novel phage-borne bicomponent leukocidin related to the Panton-Valentine leukocidin
Source: Sci Rep. 2021 Dec 22;11:24394. doi: 10.1038/s41598-021-03823-6 (PMC8695587; doi:10.1038/s41598-021-03823-6)
Supplement: Supplementary file 1 — Supplementary Information. [file 41598_2021_3823_MOESM1_ESM.zip › Supplemental File 4_Homologies of lukFS genes and their gene products.pdf]

Supplemental File 4a: Percentages of homology between nucleotide sequences of the *lukF* genes and between the amino acid sequences of their gene products of the *S. aureus* and *S. pseudintermedius* strains shown in the alignments in Figure 2.

lukF genes, DNA sequences

lukF-BV Beaver-WT19	lukF-BV Beaver-WT65	lukF-PV ATCC25923, CP009361.1	lukF-PV MW2, BA000033.2	lukF-PV TCH1516, CP000730.1	lukF-PV83 RF122, AJ938182.1	lukF-PV83 ED133, CP001996.1	lukF-PV83-2, JXHY01000064.1	lukQ equine phage, LT671578.1	lukF-int B62, X79188.1	lukF-int HKU-10-03, CP002439.1	
100,0	100,0	96,2	96,3	96,2	79,2	79,4	77,7	78,0	74,4	74,4	lukF-BV Beaver-WT19
	100,0	96,2	96,3	96,2	79,2	79,4	77,7	78,0	74,4	74,4	lukF-BV Beaver-WT65
		100,0	99,9	99,8	78,2	78,4	77,3	77,5	74,2	74,2	lukF-PV ATCC25923, CP009361.1
			100,0	99,9	78,3	78,5	77,2	77,4	74,1	74,1	lukF-PV MW2, BA000033.2
				100,0	78,3	78,5	77,2	77,4	74,0	74,0	lukF-PV TCH1516, CP000730.1
					100,0	99,3	87,4	81,0	72,1	72,2	lukF-PV83 RF122, AJ938182.1
						100,0	87,9	81,5	72,3	72,4	lukF-PV83 ED133, CP001996.1
							100,0	93,4	73,9	73,8	lukF-PV83-2, JXHY01000064.1
								100,0	74,3	74,2	lukQ equine phage, LT671578.1
									100,0	99,6	lukF-int B62, X79188.1
										100,0	lukF-int HKU-10-03, CP002439.1

lukF gene products, amino acid sequences

lukF-BV Beaver-WT19	lukF-BV Beaver-WT65	lukF-PV ATCC25923, CP009361.1	lukF-PV MW2, BA000033.2	lukF-PV TCH1516, CP000730.1	lukF-PV83 RF122, AJ938182.1	lukF-PV83 ED133, CP001996.1	lukF-PV83-2, JXHY01000064.1	lukQ equine phage, LT671578.1	lukF-int B62, X79188.1	lukF-int HKU-10-03, CP002439.1	
100,0	100,0	96,0	96,0	96,0	77,4	78,4	78,0	78,4	74,1	74,4	lukF-BV Beaver-WT19
	100,0	96,0	96,0	96,0	77,4	78,4	78,0	78,4	74,1	74,4	lukF-BV Beaver-WT65
		100,0	100,0	100,0	77,4	78,4	77,4	77,1	72,9	72,9	lukF-PV ATCC25923, CP009361.1
			100,0	100,0	77,4	78,4	77,4	77,1	72,9	73,2	lukF-PV MW2, BA000033.2
				100,0	77,4	78,4	77,4	77,1	72,9	73,2	lukF-PV TCH1516, CP000730.1
					100,0	98,5	89,3	81,4	72,3	72,6	lukF-PV83 RF122, AJ938182.1
						100,0	90,2	82,3	72,9	73,2	lukF-PV83 ED133, CP001996.1
							100,0	91,5	73,5	73,8	lukF-PV83-2, JXHY01000064.1
								100,0	74,4	74,7	lukQ equine phage, LT671578.1
									100,0	99,4	lukF-int B62, X79188.1
										100,0	lukF-int HKU-10-03, CP002439.1

Supplemental File 4b: Percentages of homology between nucleotide sequences of the *lukS* genes and between the amino acid sequences of their gene products of the *S. aureus* and S. *pseudintermedius* strains shown in the alignments in Figure 3.

lukS genes, DNA sequences

lukS-BV Beaver-WT19	lukS-BV Beaver-WT65	lukS-PV ATCC25923, CP009361.1	lukS-PV MW2, BA000033.2	lukS-PV TCH1516, CP000730.1	lukM-PV83 RF122, AJ938182.1	lukM-PV83 ED133, CP001996.1	lukM-PV83-2, JXHY01000064.1	lukP equine phage, LT671578.1	lukS-int B62, X79188.1	lukS-int HKU-10-03, CP002439.1	
100,0	100,0	94,7	94,5	94,5	72,1	72,1	73,2	73,3	67,0	67,1	lukS-BV Beaver-WT19
	100,0	94,7	94,5	94,5	72,1	72,1	73,2	73,3	67,0	67,1	lukS-BV Beaver-WT65
		100,0	99,8	99,8	69,9	69,7	71,2	71,3	66,9	66,9	lukS-PV ATCC25923, CP009361.1
			100,0	100,0	69,8	69,6	71,1	71,2	66,7	66,8	lukS-PV MW2, BA000033.2
				100,0	69,8	69,6	71,1	71,2	66,7	66,8	lukS-PV TCH1516, CP000730.1
					100,0	99,5	76,7	76,6	69,9	70,1	lukM-PV83 RF122, AJ938182.1
						100,0	76,7	76,6	69,8	70,0	lukM-PV83 ED133, CP001996.1
							100,0	99,9	72,1	72,2	lukM-PV83-2, JXHY01000064.1
								100,0	72,1	72,2	lukP equine phage, LT671578.1
									100,0	99,4	lukS-int B62, X79188.1
										100,0	lukS-int HKU-10-03, CP002439.1

lukS gene products, amino acid sequences

lukS-BV Beaver-WT19	lukS-BV Beaver-WT65	lukS-PV ATCC25923, CP009361.1	lukS-PV MW2, BA000033.2	lukS-PV TCH1516, CP000730.1	lukM-PV83 RF122, AJ938182.1	lukM-PV83 ED133, CP001996.1	lukM-PV83-2, JXHY01000064.1	lukP equine phage, LT671578.1	lukS-int B62, X79188.1	lukS-int HKU-10-03, CP002439.1	
100,0	100,0	91,5	91,2	91,2	69,8	69,5	68,6	68,6	64,8	64,8	lukS-BV Beaver-WT19
	100,0	91,5	91,2	91,2	69,8	69,5	68,6	68,6	64,8	64,8	lukS-BV Beaver-WT65
		100,0	99,7	99,7	68,2	67,9	67,3	67,3	65,1	65,1	lukS-PV ATCC25923, CP009361.1
			100,0	100,0	68,2	67,9	67,3	67,3	65,1	65,1	lukS-PV MW2, BA000033.2
				100,0	68,2	67,9	67,3	67,3	65,1	65,1	lukS-PV TCH1516, CP000730.1
					100,0	99,4	73,3	73,3	68,9	69,2	lukM-PV83 RF122, AJ938182.1
						100,0	73,0	73,0	68,6	68,9	lukM-PV83 ED133 CP001996.1
							100,0	99,7	75,2	75,8	lukM-PV83-2, JXHY01000064.1
								100,0	75,2	75,8	lukP equine phage, LT671578.1
									100,0	98,7	lukS-int B62, X79188.1
										100,0	lukS-int HKU-10-03, CP002439.1
