# Supplementary material for: Staphylococcus aureus isolates from Eurasian Beavers (Castor fiber) carry a novel phage-borne bicomponent leukocidin related to the Panton-Valentine leukocidin
Source: Sci Rep. 2021 Dec 22;11:24394. doi: 10.1038/s41598-021-03823-6 (PMC8695587; doi:10.1038/s41598-021-03823-6)
Supplement: Supplementary file 1 — Supplementary Information. [file 41598_2021_3823_MOESM1_ESM.zip › Supplemental File 5_agrA alignments.pdf]

Supplemental File 5: Nucleotide (a) and amino acid sequences (b) of *agrA* of WT19 and WT110 as well as of the BVL- and haemolysis-negative isolate WT111.

An asterisk indicates a stop codon.

a)

```
                           10        20        30        40        50        60        70        80        90       100       110       120
agrA Beaver-WT19   ATGAAAATTTTCATTTGCGAAGACGATCCAAAACAAAGAGAAAACATGGTTACCATTATTAAAAATTATATAATGATAGAAGAAAAGCCTATGGAAATTGCCCTCGCAACTGATAATCCT
agrA Beaver-WT110  ........................................................................................................................
agrA Beaver-WT111  ........................................................................................................................

                          130       140       150       160       170       180       190       200       210       220       230       240
agrA Beaver-WT19   TATGAGGTGCTTGAGCAAGCTAAAAATATGAATGACATAGGCTGTTACTTTTTAGATATTCAACTTTCAACTGATATTAATGGTATCAAATTAGGCAGTGAAATTCGTAAGCATGATCCA
agrA Beaver-WT110  ........................................................................................................................
agrA Beaver-WT111  ........................................................................................................................

                          250       260       270       280       290       300       310       320       330       340       350       360
agrA Beaver-WT19   GTTGGTAACATTATATTTGTTACGAGTCACAGTGAACTTACCTATTTAACGTTTGTCTACAAAGTTGCAGCGATGGATTTTATTTTTAAAGATGATCCAGCTGAATTAAGAACTCGAATT
agrA Beaver-WT110  ........................................................................................................................
agrA Beaver-WT111  ........................................................................................................................

                          370       380       390       400       410       420       430       440       450       460       470       480
agrA Beaver-WT19   ATAGACTGTTTAGAAACTGCACATACACGCTTACAATTGTTGTCTAAAGATAATAGCGTTGAAACGATTGAATTAAAACGTGGCAGTAATTCAGTGTATGTTCAATATGATGATATTATG
agrA Beaver-WT110  ........................................................................................................................
agrA Beaver-WT111  ........................................................................................................................

                          490       500       510       520       530       540       550       560       570       580       590       600
agrA Beaver-WT19   TTTTTTGAATCATCAACAAAATCTCACAGACTCATTGCCCATTTAGATAACCGTCAAATTGAATTTTATGGTAATTTAAAAGAACTGAGTCAATTAGATGATCGTTTCTTTAGATGTCAT
agrA Beaver-WT110  ........................................................................................................................
agrA Beaver-WT111  ........................................................................................................................

                          610       620       630       640       650       660       670       680       690       700       710
agrA Beaver-WT19   AATAGCTTTGTCGTCAATCGCCATAACATTGAATCTATAGATTCGAAAGAGCGAATTGTCTATTTTAAAAATAAAGAACACTGCTATGCATCGGTGAGAAACGTTAAAAAAATATAA
agrA Beaver-WT110  .....................................................................................................................
agrA Beaver-WT111  .........................................................................................................T...........
```

b)

```
                           10        20        30        40        50        60        70        80        90       100       110       120
agrA Beaver-WT19   MKIFICEDDPKQRENMVTIIKNYIMIEEKPMEIALATDNPYEVLEQAKNMNDIGCYFLDIQLSTDINGIKLGSEIRKHDPVGNIIFVTSHSELTYLTFVYKVAAMDFIFKDDPAELRTRI
agrA Beaver-WT110  ........................................................................................................................
agrA Beaver-WT111  ........................................................................................................................

                          130       140       150       160       170       180       190       200       210       220       230
agrA Beaver-WT19   IDCLETAHTRLQLLSKDNSVETIELKRGSNSVYVQYDDIMFFESSTKSHRLIAHLDNRQIEFYGNLKELSQLDDRFFRCHNSFVVNRHNIESIDSKERIVYFKNKEHCYASVRNVKKI*
agrA Beaver-WT110  ......................................................................................................................*
agrA Beaver-WT111  ...................................................................................................................*..*
```
