# Supplementary material for: Staphylococcus aureus isolates from Eurasian Beavers (Castor fiber) carry a novel phage-borne bicomponent leukocidin related to the Panton-Valentine leukocidin
Source: Sci Rep. 2021 Dec 22;11:24394. doi: 10.1038/s41598-021-03823-6 (PMC8695587; doi:10.1038/s41598-021-03823-6)
Supplement: Supplementary file 1 — Supplementary Information. [file 41598_2021_3823_MOESM1_ESM.zip › Supplemental File 7_Genes located in the lukFS-BV prophages of WT19 and WT65.pdf]

Supplemental file 5: Genes located in the *lukF/S*-BV prophages of WT19 and WT65.

ID	DESCRIPTION	ORIEN-TATION	START in WT19	END in WT19	LENGTH in WT19	START in WT65	END in WT65	LENGTH in WT65
***int*-1**	site-specific integrase, lysogeny module of *Siphoviridae*	REVERSE	322629	323834	1206	311401	312606	1206
N/A	hypothetic phage protein	REVERSE	324306	324536	231	*Not present*	*Not present*	*Not present*
N/A	hypothetic phage protein	REVERSE	324589	324930	342	*Not present*	*Not present*	*Not present*
N/A	ATP-dependent helicase	REVERSE	324936	325868	933	*Not present*	*Not present*	*Not present*
N/A	ImmA/IrrE family metallo-endopeptidase	REVERSE	325887	326345	459	*Not present*	*Not present*	*Not present*
N/A	helix-turn-helix domain-containing protein	REVERSE	326357	326692	336	*Not present*	*Not present*	*Not present*
N/A	helix-turn-helix transcriptional regulator	FORWARD	326856	327067	212	*Not present*	*Not present*	*Not present*
**DUF2829**	DUF2829 domain-containing protein	FORWARD	327095	327310	216	*Not present*	*Not present*	*Not present*
N/A	hypothetical protein	REVERSE	327299	327628	330	*Not present*	*Not present*	*Not present*
N/A	KilA-N domain-containing protein	REVERSE	*Not present*	*Not present*	*Not present*	312665	313495	831
N/A	hypothetical protein	REVERSE	*Not present*	*Not present*	*Not present*	313598	313942	345
**SAXN108_0309**	hypothetic protein, SAXN108_0309	REVERSE	*Not present*	*Not present*	*Not present*	313990	314175	186
N/A	hypothetical protein	REVERSE	*Not present*	*Not present*	*Not present*	314172	314318	147
N/A	XRE family transcriptional regulator	REVERSE	*Not present*	*Not present*	*Not present*	314330	315049	720
cro/hth	helix-turn-helix transcriptional Cro regulator, lysogeny module of *Siphoviridae*	FORWARD	*Not present*	*Not present*	*Not present*	315191	315409	219
N/A	hypothetical protein	FORWARD	*Not present*	*Not present*	*Not present*	315425	315676	252
N/A	hypothetical protein	FORWARD	*Not present*	*Not present*	*Not present*	315634	315777	144
N/A	hypothetical protein	REVERSE	*Not present*	*Not present*	*Not present*	315787	316389	603
N/A	phage antirepressor KilAC domain-containing protein	FORWARD	*Not present*	*Not present*	*Not present*	316446	317195	750
N/A	hypothetical protein	FORWARD	*Not present*	*Not present*	*Not present*	317210	317443	234
N/A	hypothetical protein	REVERSE	*Not present*	*Not present*	*Not present*	317406	317876	471
"rha"	Rha family transcriptional regulator	FORWARD	327679	328455	777	317933	318733	801
N/A	pathogenicity island protein	FORWARD	328469	328684	216	318747	318962	216
N/A	hypothetical protein	FORWARD	328737	328931	195	319013	319207	195

ID	DESCRIPTION	ORIEN-TATION	START in WT19	END in WT19	LENGTH in WT19	START in WT65	END in WT65	LENGTH in WT65
**DUF2513**	DUF2513 domain-containing protein	REVERSE	328926	329282	357	319202	319558	357
N/A	hypothetical protein	FORWARD	*Not present*	*Not present*	*Not present*	319619	319927	309
dbp	DNA-binding protein from *Sipho-/Phietavirus*/conserved hypothetical protein, SACOL0333	FORWARD	329337	329600	264	319957	320220	264
**DUF1270**	DUF1270 family protein, replication or lysogeny module of *Siphoviridae*, overlapping with hypothet phage protein	FORWARD	329613	329774	162	320233	320400	168
N/A	hypothet phage protein from *Sipho-/Phietavirus*, overlapping with “DUF1270”	REVERSE	329633	329743	111	320253	320363	111
N/A	hypothetical protein	FORWARD	*Not present*	*Not present*	*Not present*	320401	320721	321
**DUF1108**	lysogeny-assoc. DUF1108 family protein from *Siphoviridae*	FORWARD	*Not present*	*Not present*	*Not present*	320814	321074	261
N/A	siphovirus Gp157 family protein	FORWARD	*Not present*	*Not present*	*Not present*	321088	321567	480
N/A	ERF family protein	FORWARD	*Not present*	*Not present*	*Not present*	321567	322205	639
N/A	hypothetical protein	FORWARD	*Not present*	*Not present*	*Not present*	322205	322648	444
N/A	NUMOD4 motif-containing HNH endonuclease	FORWARD	*Not present*	*Not present*	*Not present*	322661	323202	542
N/A	hypothetical protein	FORWARD	*Not present*	*Not present*	*Not present*	323208	323876	669
N/A	hypothetic phage protein from *Sipho-/Phietavirus*	FORWARD	*Not present*	*Not present*	*Not present*	323878	324207	330
N/A	hypothetic phage protein from *Sipho-/Phietavirus*	REVERSE	*Not present*	*Not present*	*Not present*	324062	324472	411
**Q4ZAK4-rep**	putative bacteriophagal protein/replisome-organizer, replication module of *Siphoviridae*	FORWARD	*Not present*	*Not present*	*Not present*	324534	325295	762
**DNA-*rep***	DNA-replication protein from *Sipho-/Phietavirus*	FORWARD	*Not present*	*Not present*	*Not present*	325308	326093	786
sri	staphylococcal replication inhibitor	FORWARD	*Not present*	*Not present*	*Not present*	326090	326248	159
**DUF3269**	DUF3269 family protein, SACOL0345, replication module of *Siphoviridae*	FORWARD	*Not present*	*Not present*	*Not present*	326261	326482	222
**DUF1064**	DUF1064 domain-containing protein, replication module of *Siphoviridae*	FORWARD	*Not present*	*Not present*	*Not present*	326493	326897	405
N/A	hypothetical protein	FORWARD	329877	330128	252	*Not present*	*Not present*	*Not present*
N/A	MBL fold metallo-hydrolase	FORWARD	330156	330767	612	*Not present*	*Not present*	*Not present*

ID	DESCRIPTION	ORIEN-TATION	START in WT19	END in WT19	LENGTH in WT19	START in WT65	END in WT65	LENGTH in WT65
trmB	TrmB family transcriptional regulator	FORWARD	330782	331105	324	*Not present*	*Not present*	*Not present*
**DUF1351**	DUF1351 domain-containing protein	FORWARD	331116	331874	759	*Not present*	*Not present*	*Not present*
N/A	hypothetical protein	FORWARD	331895	332530	636	*Not present*	*Not present*	*Not present*
N/A	hypothetical protein	FORWARD	332551	334284	1734	*Not present*	*Not present*	*Not present*
N/A	DNA primase	FORWARD	334305	336161	1857	*Not present*	*Not present*	*Not present*
N/A	hypothetical protein	FORWARD	336158	336358	201	*Not present*	*Not present*	*Not present*
N/A	helix-turn-helix domain-containing protein	FORWARD	336355	336750	396	*Not present*	*Not present*	*Not present*
**DUF3113**	DUF3113 family protein, replication module of *Siphoviridae*	FORWARD	337228	337413	186	326902	327087	186
N/A	conserved hypothetical protein, SACOL0350	FORWARD	337414	337770	357	327088	327444	357
**ORF-51_phi-PVL**	phi PVL orf 51-like protein	FORWARD	337774	338016	243	327448	327690	243
N/A	hypothetical protein	FORWARD	338030	338440	411	*Not present*	*Not present*	*Not present*
**DUF1024**	DUF1024 family protein, replication module of *Siphoviridae*	FORWARD	338437	338685	249	327705	327953	249
**dut-phi**	dUTP pyrophosphatase	FORWARD	338678	339085	408	327946	328455	510
N/A	hypothetical protein	FORWARD	339176	339436	261	328516	328776	261
N/A	hypothetical protein	FORWARD	339453	339698	246	328814	328999	186
**DUF1381**	DUF1381 domain-containing protein, replication module of *Siphoviridae*	FORWARD	339900	340106	207	328996	329202	207
N/A	conserved hypothetical phage protein, SAAV_2037	FORWARD	340103	340345	243	329199	329441	243
**DUF1514**		FORWARD	340345	340545	201	329441	329641	201
**rinA**	RinA family transcriptional activator, replication module of *Siphoviridae*	FORWARD	340573	340989	417	329669	330085	417
**nuc-HNH**	NCTC13712_00795, HNH endonuclease	FORWARD	341221	341520	300	330317	330616	300
**SAXN108_0348**	phage protein, SAXN108_0348	FORWARD	341652	341996	345	330747	331091	345
**terL**	bacteriophagal terminase from *Sipho-/Biseptimavirus*	FORWARD	341993	343654	1662	331088	332749	1662
**portal-1**	phage portal protein	FORWARD	343670	344857	1188	332765	333952	1188
***clpP*-Q6GF91**	Clp protease ClpP	FORWARD	344841	345578	738	333936	334673	738

ID	DESCRIPTION	ORIEN-TATION	START in WT19	END in WT19	LENGTH in WT19	START in WT65	END in WT65	LENGTH in WT65
***mcp*-A0EWZ3**	bacteriophage major capsid protein	FORWARD	345602	346747	1146	334698	335843	1146
N/A	hypothetical protein	FORWARD	346767	347051	285	335863	336147	285
htcp1	hypothetical protein/phage head-tail adapter protein	FORWARD	347041	347325	285	336137	336430	294
htcp2	head-tail adaptor protein	FORWARD	347309	347671	363	336405	336767	363
**SAXN108_0356**	phage protein, SAXN108_0356, HK97 gp10 family phage protein	FORWARD	347668	348072	405	336764	337168	405
**SAXN108_0357**	phage protein, SAXN108_0357	FORWARD	348069	348476	408	337165	337572	408
**tail-L**	phage tailfiber/tail protein	FORWARD	348477	349118	642	337573	338214	642
N/A	Ig-like domain-containing protein	FORWARD	349079	349384	306	338175	338480	306
**Q7A4M4_phiNM3**	NCTC13712_00807, Uncharacterised protein	FORWARD	349435	349785	351	338531	338881	351
**SAXN108_0361**	phage protein, SAXN108_0361	FORWARD	349836	349973	138	338932	339069	138
***tmpM*-1**	phage tail tape measure protein from *Sipho-/Biseptimavirus*	FORWARD	350030	354559	4530	339126	343637	4512
**Q8SDK3-tail**	putative bacteriophagal protein from *Sipho-/Biseptimavirus*	FORWARD	354556	356040	1485	343634	345118	1485
N/A	hypothetical protein	FORWARD	356056	359736	3681	345134	348814	3681
N/A	hypothetical protein	FORWARD	359723	359875	153	348801	348953	153
N/A	hypothetical protein	FORWARD	359922	360209	288	349000	349287	288
N/A	hypothetical protein	FORWARD	360304	360672	369	349382	349750	369
hltx	putative holin-like toxin	FORWARD	360855	361002	148	349933	350080	148
sprFG	small pathogenicity island RNA F and G		361003	361193	191	350081	350280	200
holA-2	holin, lysis module of *Siphoviridae*	FORWARD	361202	361456	255	350289	350543	255
N/A	CHAP domain-containing protein	FORWARD	361468	362223	756	350555	351310	756
sprX	noncoding small regulatory RNA		362586	362735	150	351673	351822	150
N/A	SH3 domain-containing protein	FORWARD	362999	363330	332	352086	352418	333
***lukS*-BV**	Beaver-specific leukocidin, subunit S	FORWARD	363719	364657	939	352807	353745	939
***lukF*-BV**	Beaver-specific leukocidin, subunit F	FORWARD	364659	365636	978	353747	354724	978
